# Huntingtin: A Protein with a Peculiar Solvent Accessible Surface

**DOI:** 10.3390/ijms22062878

**Published:** 2021-03-12

**Authors:** Giulia Babbi, Castrense Savojardo, Pier Luigi Martelli, Rita Casadio

**Affiliations:** 1 Biocomputing Group, University of Bologna, Via San Giacomo 9/2, 40126 Bologna, Italy; giulia.babbi3@unibo.it (G.B.); castrense.savojardo2@unibo.it (C.S.); rita.casadio@unibo.it (R.C.); 2 Institute of Biomembranes, Bioenergetics and Molecular Biotechnologies, National Research Council, Via Giovanni Amendola 122/O, 70126 Bari, Italy

**Keywords:** huntingtin, protein surface annotation, protein–protein interaction, protein–membrane interactions, calcium ion–binding site

## Abstract

Taking advantage of the last cryogenic electron microscopy structure of human huntingtin, we explored with computational methods its physicochemical properties, focusing on the solvent accessible surface of the protein and highlighting a quite interesting mix of hydrophobic and hydrophilic patterns, with the prevalence of the latter ones. We then evaluated the probability of exposed residues to be in contact with other proteins, discovering that they tend to cluster in specific regions of the protein. We then found that the remaining portions of the protein surface can contain calcium-binding sites that we propose here as putative mediators for the protein to interact with membranes. Our findings are justified in relation to the present knowledge of huntingtin functional annotation.

## 1. Introduction

Huntingtin is an essential protein for early embryonic development in many species, encoded by *HTT*, a gene whose major characteristic, when mutated, is to be associated to the Huntington disease (HD) (UniProt accession code P42858 [1]). Huntingtin is ubiquitously expressed in adult animals, with the highest level in the brain [2,3]. It is mainly localized in the cytoplasm, in endosomes [4,5] and in the nuclear matrix [3].

In the past 30 years, huntingtin has been the focus of many studies given its association to HD. As a result, several different functions for the wild-type protein emerged (for review see [6]). Among other things and relevant to our study, huntingtin seems to play a role in microtubule-mediated transport [7,8] and in vesicle formation and transport [9,10].

The human huntingtin comprises 3142 residues: the CAG repetition at the 5′ end of the coding sequence of the *HTT* gene is strongly associated with the Huntington disease, generating a protein variant with an increasing number of glutamine residues (Gln, Q) at the N-terminal domain. It is believed that the disease is caused by the gain of function of the mutant [11]; other studies report several competing mechanisms for disease insurgence [12], summing direct effects from exon 1 of a mutated *HTT* (*mHTT*), with the propensity of *mHTT* to form abnormal aggregates and its indirect effects on cellular proteostasis. Indeed, huntingtin interacts with a huge number of proteins, although molecular details of these interactions are still lacking [6]. It is documented that huntingtin undergoes different post-translational modifications (PTM), including phosphorylation, acetylation, palmitoylation, ubiquitylation and SUMOylation, and, confirming that, it can also take part in different biological processes [6].

Huntingtin interacts with membranes [6]. Lipid overlay experiments indicate that huntingtin binds phosphatidylinositol phosphates and a set of anionic phospholipids via possible electrostatic interactions [13]. Mutant huntingtin with an elongated polyQ region is also capable of disrupting lipid bilayers [14], altering the interaction with phospholipids [15]. Two membrane-binding regions have been identified in huntingtin, both within the N-terminal domain: an exon 1 fragment (1–88) which may form an amphipathic helix [13,16] and a putative larger region at residues 168–366 [13]. The presence of additional membrane-binding regions distal to the N-terminus was also suggested [17].

Recently, cryogenic electron microscopy (cryoEM) helped to obtain and release a high-resolution and high-coverage three-dimensional (3D) structure of huntingtin in complex with *HTT*-associated protein 40 (*HAP40*) (release date: 17 June 2020, PDB ID: 6X9O, https://www.rcsb.org/structure/6X9O (accessed on 28 December 2020)) [18,19]. The protein’s atomic coordinates, although still with some missing segments, including the HD-linked N-terminus and a central portion, offer the possibility of studying the biophysical properties of a large fraction of the huntingtin protein surface and of characterizing regions involved in protein–protein and protein–membrane interactions at the residue level with consolidated computational approaches.

Indeed, thanks to advanced technologies, including machine and deep learning-based methods, it is possible to infer properties at the level of a single residue to highlight unexplored molecular features of the protein and seek for the tendency of the protein to participate in possible protein–protein or protein–membrane interactions [20].

Here, we focus on the protein solvent accessible surface for a better understanding of the huntingtin interactions with other proteins and with membranes by adopting ISPRED4 [21] for detecting protein–protein interaction sites and FEATURE for detecting calcium ion-binding sites [22], respectively.

Our results indicate that a large fraction of the protein solvent accessible surface is polar and that at least 192 residues are involved in protein–protein interactions. Furthermore, we found that some 81 residues are likely to be involved in calcium ion binding, and that they are mainly clustered in the regions where protein–protein interactions do not occur. Considering that we are possibly working on a specific conformation of the protein due to the stabilizing interaction with *HAP40*, we suggest that polar regions where calcium ion binding occurs are likely to interact with membranes, possibly in a calcium ion-regulated mode.

## 2. Results

### 2.1. Analysis of 3D Structures of Huntingtin

The first structure of human huntingtin obtained with cryoEM has been released in 2018. Huntingtin is in complex with *HTT*-associated protein 40 (*HAP40*), clamping one of the many different conformations that the protein may assume [23]. This cryoEM structure has an overall resolution of 4Å (PDB ID: 6EZ8, https://www.rcsb.org/structure/6EZ8 (accessed on 28 December 2020)), [24,25]. This structure covers about 75% of the total 3142 huntingtin protein sequence residues (UniProt code: P42858, region 91–3138) [1]; 2353 residues are present in 6EZ8, with the N-terminal segment missing (1–91) and with 20 gaps in the protein backbone (the largest located in position 403–660). This first full-length huntingtin structure is largely α-helical, consisting of three major domains: the N-terminal domain, the bridge domain and the C-terminal domain. The N- and the C-terminal domains fold mainly in HEAT repeats, modules of alpha helix pairs connected by short loops [25]. Arrays of HEAT repeats form rod-like helical structures and appear to function as protein–protein interaction surfaces (https://www.ebi.ac.uk/interpro/entry/InterPro/IPR000357 (accessed on 28 December 2020)) [26]. Apparently, the many HEAT repeats in the protein can be accommodated by two major domains, named N-HEAT and C-HEAT and connected by a shorter central BRIDGE domain (for details see [25]). In the same paper, regions of particular interest are discussed: two membrane-binding regions previously found, both within the N-HEAT domain. However, the first one, encoded by the N-terminal exon 1, is not present, while the second one is a large region at residues 168–366 which contains a functionally important palmitoylation site at cysteine 208 [13].

Recently, a new cryoEM structure of huntingtin in complex with the same *HAP40* protein has been released. It is characterized by a higher resolution (2.6 Å) and a higher coverage (77%) (PDB ID 6X9O, https://www.rcsb.org/structure/6X9O (accessed on 28 December 2020)) [18,19]. In sequence region 97–3139 including 3042 residues, only 2425 residues are solved in the 6X9O PDB file due to 18 gaps, the longest being in position 407–665.

The superimposition of 6EZ8 and 6X9O gives a root mean square deviation (RMSD) of 1.70 Å at the level of the protein backbone, computed over 2343 equivalent positions sharing 97.54% identity. N-HEAT, C-HEAT and BRIDGE domains and the large membrane-interacting region are conserved.

In both structures, no density was observed for the huntingtin fragment encoded by exon 1 (included in residues 1–90 in 6EZ8 and 1–96 in 6X9O, respectively). Region 1–64 of huntingtin was separately crystallized: it consists of an amino-terminal alpha helix, poly17Q region, adopting multiple conformations (including alpha helix, random coil and extended loop), and a polyproline helix formed by the proline-rich region (for the complete list of PDB files see the UniProt protein file, P42858) [1]. This supports the notion that the N-terminal region of the protein is extremely flexible [25,27] and that the length of the polyglutamine chain may have limited influence on the overall architecture of the *HTT–HAP40* complex [25].

Unfortunately, the available structures for huntingtin do not cover the gap between the first fragment 1–64 and the largest structures starting at residue 90; a full-coverage structure is therefore still missing. 

For this study, we adopted 6X9O as the reference structure, considering that this folding with the highest resolution and largest coverage is presently the only available for inspecting a large fraction of the protein solvent accessible surface.

### 2.2. Characterization of the Huntingtin Protein Solvent Accessible Surface

**Figure 1:**
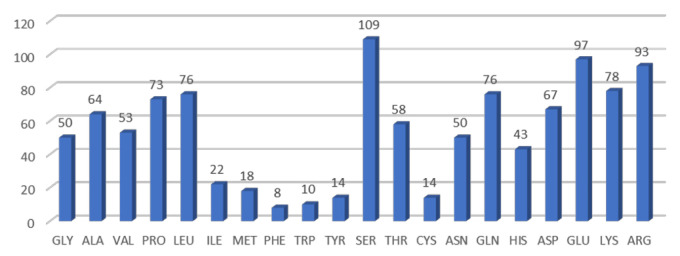
Occurrence of residue types on the protein solvent accessible surface. Surface includes residues having RSA ≥ 20% ([30], see text for details); figures over the columns indicate the absolute number of each residue type on the protein surface, labelled following the amino acids three letters code.

First, we analyzed the huntingtin protein surface with the Define Secondary Structure Program (DSSP) [28,29], which computes the solvent accessible surface area (SASA). Relative solvent accessibility (RSA) is computed by normalizing to the maximal accessible area of each residue [30]. The protein surface is the one including all residues with RSA ≥ 20% [30] and it is equal to 100,939 Å. Out of the 2425 solved residues in the protein structure (6X9O), 1073 have RSA ≥ 20%. The occurrence of the different residue types on the protein surface is reported in Figure 1: 64% of the residues are polar, including charged residues (31% of the total) and 36% are non-polar. The most frequent are serine (SER), followed by glutamic acid (GLU), arginine (ARG), lysine (LYS), leucine (LEU) and glutamine (GLN). Interestingly, GLU and LYS, followed by aspartate (ASP), histidine (HIS), tyrosine (TYR) and cysteine (CYS) (Figure 1) are also endowed with pKa values, as experimentally determined in 157 other proteins (listed in PKAD, a database of ionizable groups in proteins [31]), and this supports the observation that huntingtin surface is highly polar.

We computed the hydrophilicity/hydrophobicity of each exposed residue adopting the Kyte and Doolittle scale (KD) [32] and averaging each residue value over a 6Å radius region centered at the exposed residue. The KD values of exposed residues vary from −4.5 to +4.2, and the average KD value of the whole protein surface is –1.04. With this in mind, the protein-exposed surface structure can be colored according to the average value of each exposed residue. 

**Figure 2:**
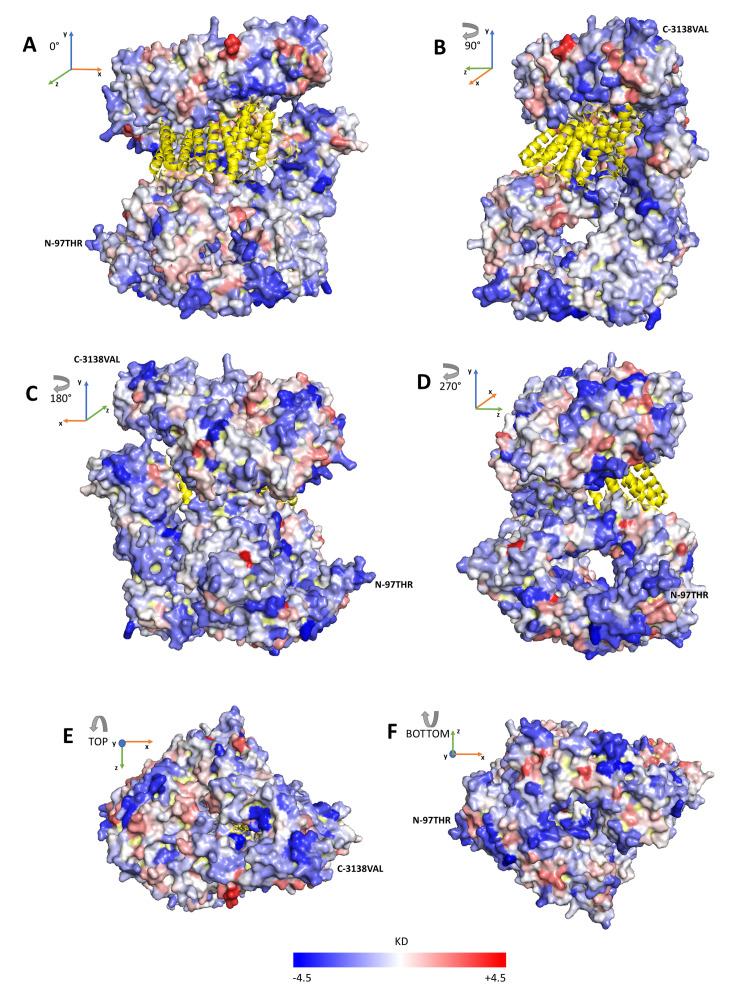
Solvent accessible surface of huntingtin. The Figure depicts the protein structure of huntingtin in complex with *HTT*-associated protein 40 *(HAP40*, colored in yellow) [19] and captured by six different views: with a clockwise rotation of the protein around the vertical axis with 90-degree steps (**A**–**D**) and the top and bottom views with respect to the vertical axis (**E**,**F**). The huntingtin surface is colored according to the average KD value obtained for each residue by averaging KD values over the 6 Å-radius neighborhood centered at the residue (KD, Kyte and Doolittle scale) [32]. The KD coloring scheme follows the blue–white–red palette (blue KD = −4.5, white KD = 0, red KD = +4.5). N- and C-termini of the protein are highlighted.

In Figure 2, we show six different views of the protein surface, rotating clockwise around the vertical axis with 90-degree steps (A–D) and around the horizontal axis (top and bottom views (E,F)). We find that hydrophobic and hydrophilic regions are scattered and intermixed over the six protein views. We conclude that huntingtin solvent accessible surface is largely polar and accordingly that the protein can have a high tendency towards electrostatic stabilizing interactions with water dipoles, other proteins and eventually anionic polar heads of membrane lipids [13].

### 2.3. Characterization of Huntingtin Protein–Protein Interaction Sites

**Figure 3:**
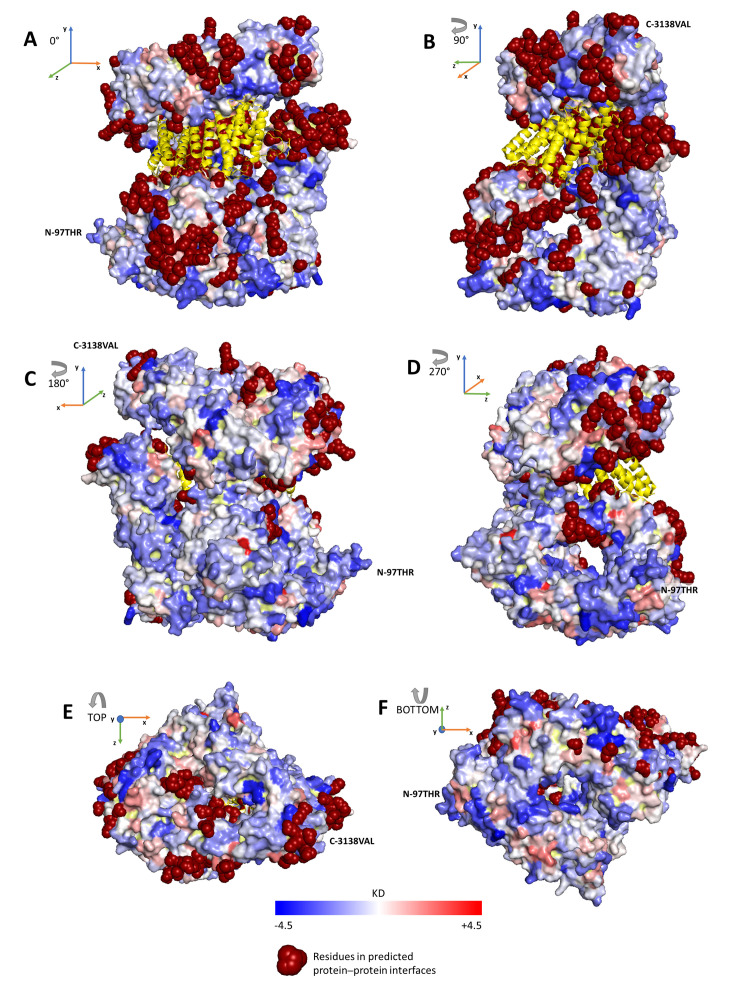
Surface residues labeled as putative interaction sites. The Figure depicts the protein structure of huntingtin in complex with *HTT*-associated protein 40 *(HAP40*, colored in yellow) [19] and captured by six different views: with a clockwise rotation of the protein around the vertical axis with 90-degree steps (**A**–**D**) and the top and bottom views with respect to the vertical axis (**E**,**F**). Dark-red spheres represent surface residues labeled as interacting sites by ISPRED4 [21] (see Materials and Methods, Table S1).

**Figure 4:**
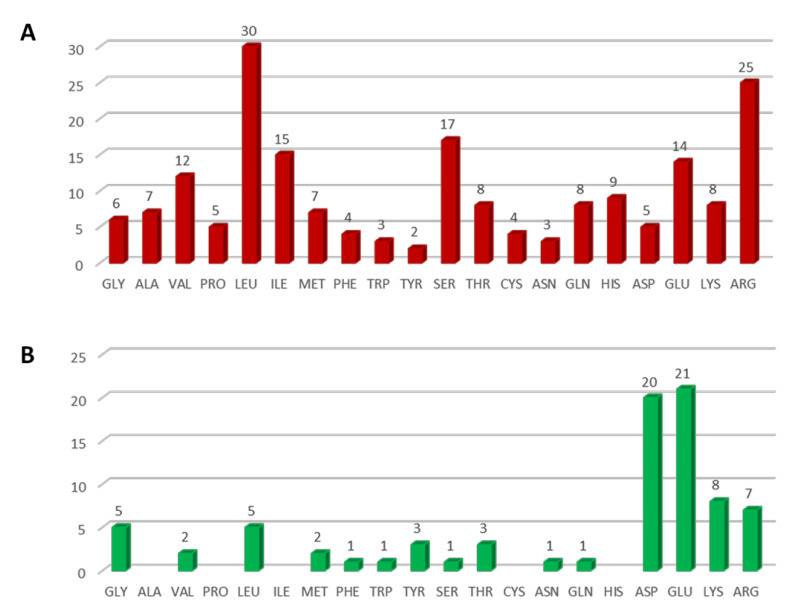
(**A**) Occurrence of surface residue types labeled as interacting by ISPRED4 [21]. The total number of residues predicted as interacting is 192 (Table S1). (**B**) Frequency of occurrence of surface residue types in the 3.5 Å-radius neighborhood of the 34 calcium-binding sites predicted with a 95% precision by FEATURE [22] (Table S1). Numbers over the columns indicate the absolute number, column labels follow the amino acids three letters code.

ISPRED4 [21] is a tool recently updated in our lab (https://ispred4.biocomp.unibo.it/ispred/ (accessed on 28 December 2020)), which is suited for computing the likelihood of a surface residue to be or not to be an interaction site. The tool is based on machine-learning approaches and it performs at the state-of-the-art level [21]. We discovered that 192 residues of the protein surface are likely to be interaction sites, therefore, they could be part of protein–protein interfaces (see Table S1 for details). In Figure 3, we show how interaction sites are distributed on the protein surface. A striking feature is that residues predicted in putative protein–protein interfaces are not homogeneously distributed and mainly cluster on sides A, B and E of the protein (Figure 3). Interestingly, 53% of the interacting residues are polar and 47% of them are non-polar (Figure 4A).

The high number of putative interaction sites well agrees with the high number of possible interactors that can be retrieved from such databases as IntAct [33,34] and BioGRID [35,36]. Considering only physical interactions filtered out by quality in IntAct, as reported by UniProt with the curated annotation [1], we could collect 98 unique genes, 84 of which are endowed with UniProt-curated subcellular locations (see Supplementary Table S2). The most frequent terms of annotation of subcellular locations are “nucleus” (44/84), “membrane” (32/84), “cytosol or cytoplasm” (55/84), “cytoskeleton” (20/84). Interestingly, 8/84 are annotated to be localized in “endosomes”, 8/84 are annotated to be localized in “Golgi”; among these, three are annotated to be localized in “Golgi and endosome”, supporting the likely cell scenario where huntingtin can interact with other proteins.

We can take into consideration the notion of “interaction patch” introduced previously [21], which groups residues predicted as interaction sites and having a Cα–Cα distance lower than 6Å. We find that the huntingtin surface has 87 interaction patches, quite in agreement with the number of high-quality and well-curated interactors (98) described above. The interaction patches (not shown) cluster again in views A, B and E of the protein (Figure 3).

Our analysis supports the notion that huntingtin serves as a protein–protein interaction hub [6,37] and that it is involved in interactions with proteins localized in the cytoplasm, in endosomes and other membrane systems [4,5].

### 2.4. Characterization of Possible Lipid Membrane-Binding Regions of Huntingtin

**Figure 5:**
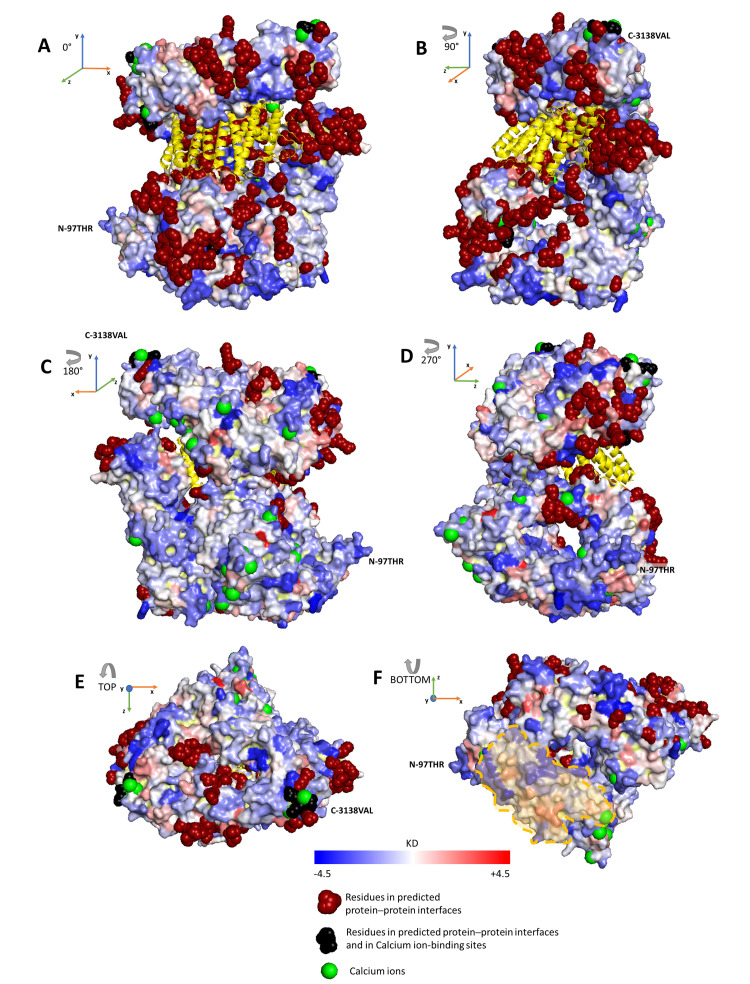
The computed calcium-binding sites of the huntingtin protein surface. The Figure depicts the protein structure of huntingtin in complex with *HTT*-associated protein 40 *(HAP40*, colored in yellow) [19] and captured by six different views: with a clockwise rotation of the protein around the vertical axis with 90-degree steps (**A**–**D**) and the top and bottom views with respect to the vertical axis (**E**,**F**). Calcium ions are showed as green spheres with a 2.0 Å radius and computed with FEATURE [22]. Thirty-four calcium binding sites were predicted with a 95% precision. Dark-red spheres are atoms of residues predicted as interacting by ISPRED4 [21]; black spheres are atoms of interacting residues found in the 3.5 Å-radius neighborhood of the calcium ions (Table S1). We highlighted a light orange region, 166–443, described as a possible membrane-binding region [13].

Given the high percentage of hydrophilic regions on its surface, huntingtin can indeed interact with membrane lipids, as previously suggested [6]. In literature, it is well-established that a protein can interact with the membrane surface in the calcium ion-regulated manner [38]. For huntingtin, this evidence is lacking in spite of the protein being involved in Ca ion homeostasis in mitochondria [39]. Here, we computed the probability of finding Ca ion-binding sites over the huntingtin surface. We adopted FEATURE [22], a machine learning automated tool for examining biophysical and biochemical features of protein structures. We adopted the FEATURE Calcium Model for computing putative calcium-binding sites on the protein surface. Huntingtin has 34 sites computed with a 95% precision for calcium binding and these are distributed predominantly on side C of the protein (Figure 5C), where 24 calcium-binding sites are represented with green spheres.

When considering a neighborhood within 3.5 Å centered at the putative Ca ions, 81 residues shown in Figure 4B are found (Table S1). The pattern of occurrence is similar to previous distributions described for calcium-binding sites [40], with a high percentage of charged glutamic (GLU) and aspartic (ASP) acids, as expected (accounting for the 26% and 25% of the involved residues, respectively; see Figure 4B). Seventy-seven percent of residues around the computed calcium ion-binding sites are polar. Only eight residues (ARG984, ASP2737, GLU 2738, ASP2758, LYS 2759, GLU 3106, GLU30107, GLU 3108) out of the 192 annotated as interaction sites in our analysis are also involved in calcium binding (black, as opposed to the red color in Figure 5).

We can conclude that the protein sides containing residues with very low tendency to be involved in interaction sites (Figure 5C), in turn, cluster Ca ion-binding sites that may eventually regulate protein binding to anionic phospholipids. A possible membrane system with which the protein interacts is indeed the early endosome [41].

**Figure 6:**
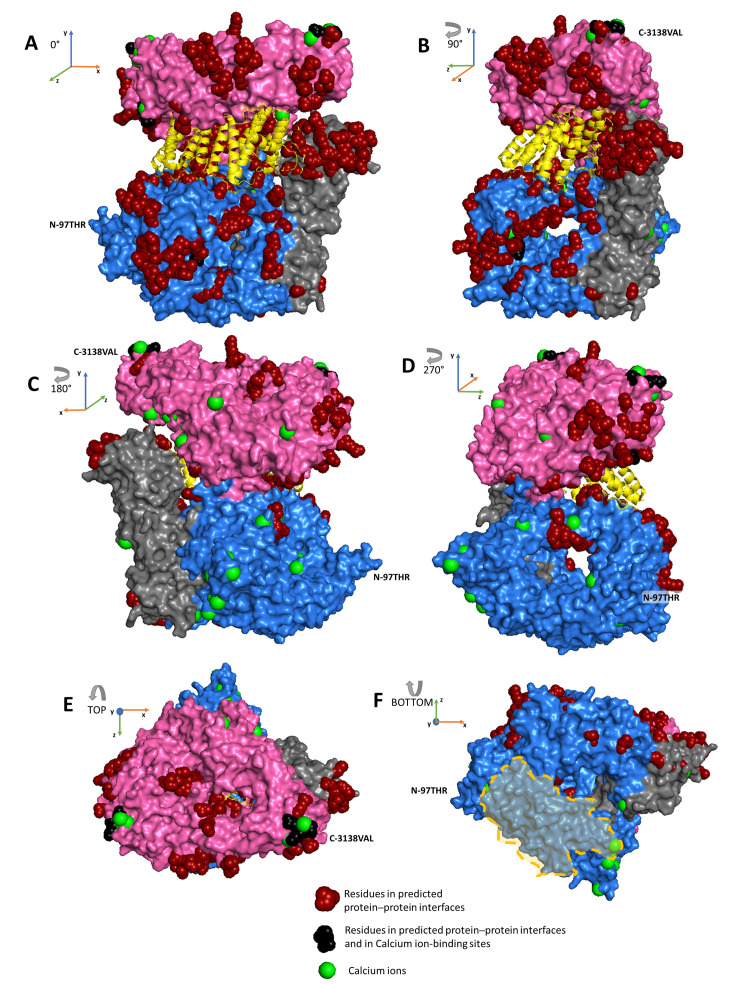
Interacting and calcium-biding sites over N-HEAT, C HEAT and BRIDGE huntingtin protein domains. The computed calcium-binding sites of the huntingtin protein surface. The Figure depicts the protein structure of huntingtin in complex with *HTT*-associated protein 40 *(HAP40*, colored in yellow) [19] and captured by six different views: with a clockwise rotation of the protein around the vertical axis with 90-degree steps (**A**–**D**) and the top and bottom views with respect to the vertical axis (**E**,**F**). For domain description, see text and Guo et al., 2018 [25]. N-HEAT (pink color), C-HEAT (blue color) and BRIDGE (gray color) domains are shown according to the six different views of the protein (see Figure 2). Interacting surface residues and calcium-binding sites are color-coded as in Figure 5. The light orange area in view F indicates the membrane-binding region described by Kegel et al., 2005 [13].

In Figure 6, we show how interaction sites and calcium-binding sites are distributed according to the N-HEAT (pink color), C-HEAT (blue color) and BRIDGE (grey color) domains previously described [25]. Evidently, the predicted interaction sites and calcium-binding sites are not limited to HEAT domains, suggesting that protein interactions may occur in different regions of the protein. The previously described region of the protein–membrane interaction located at residues 166–443 does not contain interaction sites, and calcium-binding sites are at the border (Figure 6, view F). In this region, direct electrostatic interactions may dominate protein–membrane interactions [13]. Very recently, another ion-binding site predictor, BION-2, became available [42], and by adopting the calcium ion modality, we could confirm that calcium-binding sites are not limited to HEAT domains and that two calcium-binding sites are located at the border of the 166–443 region (data not shown).

## 3. Discussion

In this paper, we characterize the solvent accessible surface of a recently released high-resolution and high-coverage huntingtin structure in complex with huntingtin-binding *HTT*-associated protein 40 (*HAP40*). The structure is solved for sequence region 97–3139, including 3042 residues. However, only 2425 residues are described in our 6X9O reference PDB file due to 18 gaps, the longest being in position 407–665. In spite of this, we can observe that the protein in this stabilized conformation, due to the presence of *HAP40*, is mainly polar (64% of the total 1073 exposed residues, including some 36% of the charged residues). Polar and non-polar regions are intermixed on the protein surface and exposed residues can be labeled taking advantage of their likelihood of being in interacting sites participating in protein–protein interactions. This is possible thanks to the development of machine learning-based tools such as ISPRED4 [21] that have been extensively benchmarked to validate their performance in inferring the property after training on internationally adopted training sets.

It is known and determined in experiments of interactomics that huntingtin can physically interact with a large number of different proteins. This is mainly due to the fact that the protein is ubiquitous and that it can participate in relevant biological processes such as vesicular transport, cell division, ciliogenesis, endocytosis, endosomal trafficking, autophagy, calcium homeostasis in mitochondria and proteostasis [6]. We found that the number of computed interacting sites well agrees with the number of the high-quality interactors listed in the UniProt sequence file (P42858). Interestingly, all the interaction sites cluster in specific zones of the protein, including but not limited to the N-HEAT, C-HEAT and BRIDGE domains (Figure 5) [25]. All the predicted interacting sites when variated may hamper functional interactions of huntingtin with other genes important for many biological processes to which it contributes and elicit different disorders. By exploring with eDGAR [42] (https://edgar.biocomp.unibo.it (accessed on 28 December 2020)) the complex space of gene–disease relations and taking advantage of the 98 gene interactors, we were able to associate huntingtin to some 43 more diseases different from HD (data not shown).

Our effort here was mainly devoted to exploring the protein surface (of possibly one of the many conformations that the protein may have in a solution) to understand whether predicted interaction sites are consistent with the huge number of interactors that the protein seems to have in interactomics experiments. It is possible that this number may change depending on the different conformations that the protein most likely acquires in a solution. Unfortunately, these data are not yet available. Neither is the complete structure of the protein with the polyQ tail, and this hampers any speculation on the specific role of the tail in affecting protein–protein interactions. Interestingly, when we explored the protein surface with a predictor suited to locate protein–protein interaction sites, we found that there was a large fraction of residues that were left behind.

Prompted by previous observations on huntingtin–membrane interaction, we found that in the surface patches not containing interacting residues, we were able to allocate calcium ion-binding sites with a precision higher than 95% [43] (Figure 6). Some of these binding sites are lateral to a previously characterized membrane binding region of the protein (Figure 6F) [13]. Results are confirmed by adopting another predictor, BION-2 [42]. Considering that calcium ions can mediate protein–membrane interactions [38], we would like to propose in this paper that huntingtin–membrane interactions can also be mediated by calcium as a cofactor. Hopefully, future experiments will shine a light on this aspect.

## 4. Materials and Methods

### 4.1. Protein Structure Characterization

We used two huntingtin structures obtained via cryogenic electron microscopy: PDB ID 6EZ8 [24,25] and PDB ID [18,19], both in complex with *HTT*-associated protein 40 (HAP40).

We computed the superimposition of the two structures, 6EZ8 and 6X9O, using the online version of FATCAT 2.0 [44,45], obtaining an RMSD of 1.70 Å at the level of the protein backbone, computed over 2343 equivalent positions, sharing an identity of 97.54%.

We defined the protein surface computing the relative solvent accessibility (RSA) of protein residues using the DSSP program [28,29], considering exposed only the residues with RSA greater or equal 20%. We computed the hydrophilicity/hydrophobicity of each exposed residue with the Kyte and Doolittle scale [32], and then averaging the value over a 6 Å radius surface centered at the exposed residue.

### 4.2. Protein Structure Visualization

PyMOL is a molecular visualization tool on an open-source foundation, [46]. We use it for representing the protein structure of huntingtin in complex with the *HTT*-associated protein 40 (*HAP40*) [18,19]. The protein surface is visualized considering a solvent radius of 1.6 Å. The coloring scheme KD for averaged hydrophilicity/hydrophobicity is based on the Kyte and Doolittle scale [32] and the KD value of each residue was obtained by averaging over the 6 Å-radius range on the protein surface centered at the exposed residue.

### 4.3. Computational Methods

ISPRED4 [21] is a machine learning-based tool to infer the presence of protein–protein interaction sites on monomer surfaces. It relies on a cascade of support vector machines and grammatical-restrained hidden conditional random fields that analyze a set of 46 different descriptors extracted from the monomer sequence, the multiple sequence alignments and the monomer 3D structure. It has been trained on 151 protein complexes and, when evaluated in cross-validation, it performs at the-state-of-the-art level, reaching a Matthews correlation coefficient of 0.48 per residue and an overall accuracy of 0.85.

FEATURE [43] is a framework for modelling and recognizing functional sites in protein structures based on the analysis of local surface environments characterized with 480 different physicochemical descriptors, including electrostatics, hydrophobicity, density, and type of atoms. Weighting of the different descriptors is performed with a naïve Bayes model trained on a dataset of known examples. A specific model trained on 312 different protein structures to recognize possible binding sites for calcium ions has been released [22]. It can discriminate calcium-binding sites with more than 98% precision and a recall higher than 93% within a distance of 3.5 Å.

BION-2 [42] is a recently released predictor that utilizes the Gaussian-based treatment of ions within the framework of the modified Poisson–Boltzmann equation.

## Data Availability

No ethical issue is raised by the paper that only uses available molecular data without reference to patients.

## Supplementary Materials

The following are available online at https://www.mdpi.com/1422-0067/22/6/2878/s1, Table S1: Annotation of huntingtin residues as interacting and calcium-binding sites; Table S2: Subcellular location of huntingtin interactors.
