# Supplementary material for: Huntingtin: A Protein with a Peculiar Solvent Accessible Surface
Source: Int J Mol Sci. 2021 Mar 12;22(6):2878. doi: 10.3390/ijms22062878 (PMC8001614; doi:10.3390/ijms22062878)
Supplement: Supplementary file 1 [file ijms-22-02878-s001.zip › Table S1.pdf]

Table S1. Annotation of huntingtin residues as interacting and Calcium binding sites.

#	Residue	ACC	RSA	Surface	Interface	KD	KD mean	Helix number	Helix label	Calcium ion binding site
97	T	174	1	yes	no	-0.7	-2.3	-	-	no
98	K	89	0.434	yes	no	-3.9	-3.3	1	AA1	no
99	K	168	0.82	yes	no	-3.9	-2.32	1	AA1	no
100	D	110	0.675	yes	no	-3.5	-2.61	1	AA1	no
101	R	95	0.383	yes	no	-4.5	-2.61	1	AA1	no
102	V	34	0.239	yes	no	4.2	-1.51	1	AA1	no
103	N	84	0.535	yes	no	-3.5	-1.06	1	AA1	no
104	H	88	0.478	yes	no	-3.2	-1.06	1	AA1	no
105	C	2	-	no	-	-	-	1	AA1	no
106	L	49	0.299	yes	no	3.8	0.85	1	AA1	no
107	T	48	0.338	yes	no	-0.7	-0.43	1	AA1	no
108	I	20	0.118	no	-	4.5	0.12	1	AA1	no
109	C	0	-	no	-	-	-	1	AA1	no
110	E	107	0.552	yes	no	-3.5	-0.8	1	AA1	no
111	N	81	0.516	yes	no	-3.5	0.7	1	AA1	no
112	I	0	-	no	-	-	-	1	AA1	no
113	V	36	0.254	yes	no	4.2	0.83	-	-	no
114	A	74	0.698	yes	no	1.8	-0.25	-	-	no
115	Q	59	0.298	yes	no	-3.5	0.43	-	-	no
116	S	115	0.885	yes	no	-0.8	-1.15	-	-	no
117	V	81	0.57	yes	no	4.2	-1.15	-	-	no
118	R	73	0.294	yes	no	-4.5	-1.08	-	-	no
119	N	145	0.924	yes	no	-3.5	-2.93	-	-	no
120	S	27	0.208	yes	no	-0.8	-2.14	2	AA2	no
121	P	106	0.779	yes	no	1.6	-1.55	2	AA2	no
122	E	155	0.799	yes	no	-3.5	-0.9	2	AA2	no
123	F	1	-	no	-	-	-	2	AA2	no
124	Q	60	0.303	yes	no	-3.5	-0.5	2	AA2	no
125	K	135	0.659	yes	no	-3.9	-1	2	AA2	no
126	L	56	0.341	yes	no	3.8	0.1	2	AA2	no
127	L	0	-	no	-	-	-	2	AA2	no
128	G	11	0.131	no	-	-0.4	1	2	AA2	no
129	I	98	0.58	yes	no	4.5	1.64	2	AA2	no

#	Residue	ACC	RSA	Surface	Interface	KD	KD mean	Helix number	Helix label	Calcium ion binding site
130	A	0	-	no	-	-	-	2	AA2	no
131	M	2	-	no	-	-	-	2	AA2	no
132	E	61	0.314	yes	no	-3.5	1.6	2	AA2	yes
133	L	37	0.226	yes	no	3.8	2.15	2	AA2	no
134	F	0	-	no	-	-	-	2	AA2	no
135	L	1	-	no	-	-	-	2	AA2	no
136	L	109	0.665	yes	no	3.8	1.37	3	AA3	no
137	C	2	-	no	-	-	-	3	AA3	no
138	S	5	-	no	-	-	-	3	AA3	no
139	D	58	0.356	yes	no	-3.5	-1.7	3	AA3	no
140	D	27	0.166	no	-	-3.5	-2.17	3	AA3	no
141	A	105	0.991	yes	no	1.8	-1.73	-	-	no
142	E	76	0.392	yes	no	-3.5	-1.5	-	-	no
143	S	72	0.554	yes	no	-0.8	-3.08	4	AA4	no
144	D	103	0.632	yes	no	-3.5	-1.73	4	AA4	no
145	V	3	-	no	-	-	-	4	AA4	no
146	R	49	0.198	no	-	-4.5	-0.54	4	AA4	no
147	M	101	0.537	yes	no	1.9	-1.08	4	AA4	no
148	V	30	0.211	yes	no	4.2	-1.08	4	AA4	no
149	A	0	-	no	-	-	-	4	AA4	no
150	D	45	0.276	yes	no	-3.5	-0.22	4	AA4	no
151	E	101	0.521	yes	no	-3.5	-0.42	4	AA4	no
152	C	0	-	no	-	-	-	4	AA4	no
153	L	1	-	no	-	-	-	4	AA4	no
154	N	14	0.089	no	-	-3.5	-1.88	4	AA4	no
155	K	58	0.283	yes	no	-3.9	-1.53	4	AA4	no
156	V	0	-	no	-	-	-	4	AA4	no
157	I	0	-	no	-	-	-	4	AA4	no
158	K	22	0.107	no	-	-3.9	-1.52	4	AA4	yes
159	A	55	0.519	yes	no	1.8	-1.02	4	AA4	no
160	L	0	-	no	-	-	-	5	AA5	no
161	M	30	0.16	no	-	1.9	-0.6	5	AA5	no
162	D	75	0.46	yes	no	-3.5	-0.42	5	AA5	no

#	Residue	ACC	RSA	Surface	Interface	KD	KD mean	Helix number	Helix label	Calcium ion binding site
163	S	43	0.331	yes	no	-0.8	-1.48	5	AA5	no
164	N	23	0.146	no	-	-3.5	-0.71	5	AA5	no
165	L	17	0.104	no	-	3.8	-0.7	5	AA5	no
166	P	73	0.537	yes	no	1.6	-0.65	5	AA5	no
167	R	50	0.202	yes	no	-4.5	0.24	5	AA5	no
168	L	0	-	no	-	-	-	5	AA5	no
169	Q	1	-	no	-	-	-	5	AA5	no
170	L	73	0.445	yes	no	3.8	-0.67	5	AA5	no
171	E	3	-	no	-	-	-	5	AA5	yes
172	L	1	-	no	-	-	-	5	AA5	no
173	Y	16	0.072	no	-	-1.3	-1.23	5	AA5	no
174	K	63	0.307	yes	no	-3.9	-3.15	5	AA5	no
175	E	29	0.149	no	-	-3.5	-3.3	5	AA5	no
176	I	5	-	no	-	-	-	5	AA5	no
177	K	98	0.478	yes	no	-3.9	-3.74	5	AA5	no
178	K	133	0.649	yes	no	-3.9	-3.04	5	AA5	no
179	N	66	0.42	yes	no	-3.5	-2.92	-	-	no
180	G	33	0.393	yes	no	-0.4	-1.5	-	-	no
181	A	34	0.321	yes	no	1.8	-0.46	6	AA6	no
182	P	37	0.272	yes	no	1.6	-0.47	6	AA6	no
183	R	102	0.411	yes	no	-4.5	-1.65	6	AA6	no
184	S	10	0.077	no	-	-0.8	-1.65	6	AA6	no
185	L	2	-	no	-	-	-	6	AA6	no
186	R	87	0.351	yes	no	-4.5	-2.28	6	AA6	no
187	A	5	-	no	-	-	-	6	AA6	no
188	A	0	-	no	-	-	-	6	AA6	no
189	L	1	-	no	-	-	-	6	AA6	no
190	W	82	0.361	yes	no	-0.9	-2.7	6	AA6	no
191	R	4	-	no	-	-	-	6	AA6	no
192	F	0	-	no	-	-	-	6	AA6	no
193	A	3	-	no	-	-	-	6	AA6	no
194	E	10	0.052	no	-	-3.5	-3.5	6	AA6	yes
195	L	0	-	no	-	-	-	7	AA7	no

#	Residue	ACC	RSA	Surface	Interface	KD	KD mean	Helix number	Helix label	Calcium ion binding site
196	A	0	-	no	-	-	-	7	AA7	no
197	H	31	0.168	no	-	-3.2	-0.07	7	AA7	no
198	L	42	0.256	yes	no	3.8	-0.03	7	AA7	no
199	V	5	-	no	-	-	-	7	AA7	no
200	R	80	0.323	yes	no	-4.5	-2.13	-	-	no
201	P	26	0.191	no	-	1.6	-2.58	-	-	no
202	Q	58	0.293	yes	no	-3.5	-2.58	-	-	no
203	K	89	0.434	yes	no	-3.9	-1.67	8	AA8	no
204	C	2	-	no	-	-	-	8	AA8	no
205	R	99	0.399	yes	no	-4.5	-1.06	8	AA8	no
206	P	67	0.493	yes	no	1.6	-0.78	8	AA8	no
207	Y	14	0.063	no	-	-1.3	-1.23	8	AA8	no
208	L	1	-	no	-	-	-	8	AA8	no
209	V	61	0.43	yes	no	4.2	0.96	8	AA8	no
210	N	68	0.433	yes	no	-3.5	0.8	8	AA8	no
211	L	0	-	no	-	-	-	8	AA8	no
212	L	20	0.122	no	-	3.8	1.53	8	AA8	no
213	P	49	0.36	yes	no	1.6	1.57	8	AA8	no
214	C	5	-	no	-	-	-	8	AA8	no
215	L	1	-	no	-	-	-	8	AA8	no
216	T	20	0.141	no	-	-0.7	-0.45	8	AA8	no
217	R	110	0.444	yes	no	-4.5	-3.03	8	AA8	no
218	T	2	-	no	-	-	-	8	AA8	no
219	S	1	-	no	-	-	-	8	AA8	no
220	K	151	0.737	yes	no	-3.9	-4.3	8	AA8	no
221	R	39	0.157	no	-	-4.5	-2.58	-	-	no
222	P	99	0.728	yes	no	1.6	-2.13	-	-	no
223	E	86	0.443	yes	no	-3.5	-2.14	9	AA9	no
224	E	107	0.552	yes	no	-3.5	-2.6	9	AA9	no
225	S	39	0.3	yes	no	-0.8	-2.83	9	AA9	no
226	V	1	-	no	-	-	-	9	AA9	no
227	Q	1	-	no	-	-	-	9	AA9	no
228	E	94	0.485	yes	no	-3.5	-0.8	9	AA9	no

#	Residue	ACC	RSA	Surface	Interface	KD	KD mean	Helix number	Helix label	Calcium ion binding site
229	T	10	0.07	no	-	-0.7	-1.02	9	AA9	no
230	L	0	-	no	-	-	-	9	AA9	no
231	A	30	0.283	yes	no	1.8	-0.15	9	AA9	no
232	A	54	0.509	yes	no	1.8	0.97	9	AA9	no
233	A	2	-	no	-	-	-	9	AA9	no
234	V	0	-	no	-	-	-	9	AA9	no
235	P	45	0.331	yes	no	1.6	-0.32	9	AA9	no
236	K	70	0.341	yes	no	-3.9	-0.17	9	AA9	no
237	I	0	-	no	-	-	-	9	AA9	no
238	M	3	-	no	-	-	-	9	AA9	no
239	A	43	0.406	yes	no	1.8	-0.9	9	AA9	no
240	S	11	0.085	no	-	-0.8	-0.73	9	AA9	no
241	F	0	-	no	-	-	-	9	AA9	no
242	G	1	-	no	-	-	-	9	AA9	no
243	N	51	0.325	yes	no	-3.5	-1.38	10	AB1	no
244	F	15	0.076	no	-	2.8	-0.85	10	AB1	no
245	A	6	0.057	no	-	1.8	-0.6	10	AB1	no
246	N	74	0.471	yes	no	-3.5	-2.44	11	AB2	no
247	D	38	0.233	yes	no	-3.5	-3.5	11	AB2	no
248	N	117	0.745	yes	no	-3.5	-3.58	11	AB2	no
249	E	27	0.139	no	-	-3.5	-2.28	11	AB2	no
250	I	2	-	no	-	-	-	11	AB2	no
251	K	38	0.185	no	-	-3.9	-1.67	11	AB2	no
252	V	74	0.521	yes	no	4.2	-1.77	11	AB2	no
253	L	1	-	no	-	-	-	11	AB2	no
254	L	0	-	no	-	-	-	11	AB2	no
255	K	120	0.585	yes	no	-3.9	1.65	11	AB2	no
256	A	19	0.179	no	-	1.8	0.43	11	AB2	no
257	F	0	-	no	-	-	-	11	AB2	no
258	I	18	0.107	no	-	4.5	0.14	11	AB2	no
259	A	51	0.481	yes	no	1.8	0.93	11	AB2	no
260	N	24	0.153	no	-	-3.5	-1.07	12	AB3	no
261	L	0	-	no	-	-	-	12	AB3	no

#	Residue	ACC	RSA	Surface	Interface	KD	KD mean	Helix number	Helix label	Calcium ion binding site
262	K	132	0.644	yes	no	-3.9	-0.12	12	AB3	no
263	S	24	0.185	no	-	-0.8	-1.96	12	AB3	no
264	S	97	0.746	yes	no	-0.8	-0.8	-	-	no
265	S	20	0.154	no	-	-0.8	0.5	13	AB4	no
266	P	73	0.537	yes	no	1.6	0.02	13	AB4	no
267	T	27	0.19	no	-	-0.7	-0.73	13	AB4	no
268	I	20	0.118	no	-	4.5	-0.73	13	AB4	no
269	R	27	0.109	no	-	-4.5	-1.18	13	AB4	no
270	R	109	0.44	yes	no	-4.5	-1.3	13	AB4	no
271	T	1	-	no	-	-	-	13	AB4	no
272	A	0	-	no	-	-	-	13	AB4	no
273	A	0	-	no	-	-	-	13	AB4	no
274	G	25	0.298	yes	no	-0.4	1	13	AB4	no
275	S	0	-	no	-	-	-	13	AB4	no
276	A	0	-	no	-	-	-	13	AB4	no
277	V	9	0.063	no	-	4.2	1	13	AB4	no
278	S	9	0.069	no	-	-0.8	0.22	13	AB4	no
279	I	0	-	no	-	-	-	13	AB4	no
280	C	0	-	no	-	-	-	13	AB4	no
281	Q	66	0.333	yes	no	-3.5	-2.05	13	AB4	no
282	H	53	0.288	yes	no	-3.2	-1.63	13	AB4	no
283	S	5	-	no	-	-	-	-	-	no
284	R	105	0.423	yes	no	-4.5	-3.3	-	-	no
285	R	115	0.464	yes	no	-4.5	-2.9	14	AB5	no
286	T	49	0.345	yes	no	-0.7	-3	14	AB5	no
287	Q	51	0.258	yes	no	-3.5	-2.16	14	AB5	no
288	Y	43	0.194	no	-	-1.3	-1.06	14	AB5	no
289	F	0	-	no	-	-	-	14	AB5	no
290	Y	11	-	no	-	-	-	14	AB5	no
291	S	14	0.108	no	-	-0.8	-0.95	14	AB5	no
292	W	55	0.242	yes	no	-0.9	-1.73	14	AB5	no
293	L	0	-	no	-	-	-	14	AB5	no
294	L	1	-	no	-	-	-	14	AB5	no

#	Residue	ACC	RSA	Surface	Interface	KD	KD mean	Helix number	Helix label	Calcium ion binding site
295	N	94	0.599	yes	no	-3.5	0.64	14	AB5	no
296	V	37	0.261	yes	no	4.2	1.02	14	AB5	no
297	L	0	-	no	-	-	-	14	AB5	no
298	L	21	0.128	no	-	3.8	2.02	14	AB5	no
299	G	41	0.488	yes	no	-0.4	2.02	14	AB5	no
300	L	52	0.317	yes	no	3.8	2.4	14	AB5	no
301	L	5	-	no	-	-	-	-	-	no
302	V	105	0.739	yes	no	4.2	2.3	-	-	no
303	P	48	0.353	yes	no	1.6	0.6	-	-	no
304	V	49	0.345	yes	no	4.2	-0.3	-	-	no
305	E	152	0.784	yes	no	-3.5	-0.3	-	-	no
306	D	88	0.54	yes	no	-3.5	-0.94	-	-	no
307	E	180	0.928	yes	no	-3.5	-2.75	-	-	no
308	H	31	0.168	no	-	-3.2	-2.5	-	-	no
309	S	71	0.546	yes	no	-0.8	-0.1	-	-	no
310	T	63	0.444	yes	no	-0.7	1.52	15	AB6	no
311	L	58	0.354	yes	no	3.8	1	15	AB6	no
312	L	21	0.128	no	-	3.8	1	15	AB6	no
313	I	5	-	no	-	-	-	15	AB6	no
314	L	29	0.177	no	-	3.8	3.8	15	AB6	no
315	G	0	-	no	-	-	-	15	AB6	no
316	V	0	-	no	-	-	-	15	AB6	no
317	L	0	-	no	-	-	-	15	AB6	no
318	L	25	0.152	no	-	3.8	-0.35	15	AB6	no
319	T	0	-	no	-	-	-	15	AB6	no
320	L	1	-	no	-	-	-	15	AB6	no
321	R	59	0.238	yes	no	-4.5	-0.67	15	AB6	no
322	Y	76	0.342	yes	no	-1.3	-2.9	15	AB6	no
323	L	1	-	no	-	-	-	15	AB6	no
324	V	3	-	no	-	-	-	15	AB6	no
325	P	43	0.316	yes	no	1.6	1.57	16	AB7	no
326	L	48	0.293	yes	no	3.8	0.63	16	AB7	no
327	L	7	-	no	-	-	-	16	AB7	no

#	Residue	ACC	RSA	Surface	Interface	KD	KD mean	Helix number	Helix label	Calcium ion binding site
328	Q	97	0.49	yes	no	-3.5	-1.07	-	-	no
329	Q	182	0.919	yes	no	-3.5	-3.5	-	-	no
349	V	131	0.923	yes	no	4.2	0.7	-	-	no
350	S	72	0.554	yes	no	-0.8	-0.44	-	-	no
351	P	5	-	no	-	-	-	-	-	no
352	S	56	0.431	yes	no	-0.8	-1.5	17	AB8	no
353	A	26	0.245	yes	no	1.8	-0.88	17	AB8	no
354	E	94	0.485	yes	no	-3.5	-0.36	17	AB8	no
355	Q	15	0.076	no	-	-3.5	-0.88	17	AB8	no
356	L	3	-	no	-	-	-	17	AB8	no
357	V	8	0.056	no	-	4.2	-0.9	17	AB8	no
358	Q	80	0.404	yes	no	-3.5	-1.57	17	AB8	no
359	V	1	-	no	-	-	-	17	AB8	no
360	Y	0	-	no	-	-	-	17	AB8	no
361	E	53	0.273	yes	no	-3.5	-1.07	17	AB8	no
362	L	5	-	no	-	-	-	17	AB8	no
363	T	0	-	no	-	-	-	17	AB8	no
364	L	9	0.055	no	-	3.8	-0.97	17	AB8	no
365	H	73	0.397	yes	no	-3.2	-0.97	17	AB8	no
366	H	5	-	no	-	-	-	17	AB8	no
367	T	1	-	no	-	-	-	17	AB8	no
368	Q	74	0.374	yes	no	-3.5	-3.3	-	-	no
369	H	31	0.168	no	-	-3.2	-1.9	-	-	no
370	Q	168	0.848	yes	no	-3.5	-3.4	-	-	no
371	D	45	0.276	yes	no	-3.5	-3.38	18	AB9	no
372	H	67	0.364	yes	no	-3.2	-1.34	18	AB9	no
373	N	79	0.503	yes	no	-3.5	-1.34	18	AB9	no
374	V	0	-	no	-	-	-	18	AB9	no
375	V	10	0.07	no	-	4.2	-1.13	18	AB9	no
376	T	28	0.197	no	-	-0.7	-0.72	18	AB9	no
377	G	9	0.107	no	-	-0.4	-0.1	18	AB9	no
378	A	0	-	no	-	-	-	18	AB9	no
379	L	0	-	no	-	-	-	18	AB9	no

#	Residue	ACC	RSA	Surface	Interface	KD	KD mean	Helix number	Helix label	Calcium ion binding site
380	E	47	0.242	yes	no	-3.5	-2.47	18	AB9	no
381	L	0	-	no	-	-	-	18	AB9	no
382	L	0	-	no	-	-	-	18	AB9	no
383	Q	30	0.152	no	-	-3.5	-3.5	18	AB9	no
384	Q	55	0.278	yes	no	-3.5	-3.05	18	AB9	no
385	L	2	-	no	-	-	-	18	AB9	no
386	F	5	-	no	-	-	-	18	AB9	no
387	R	153	0.617	yes	no	-4.5	-2.9	18	AB9	no
388	T	39	0.275	yes	no	-0.7	-1.1	18	AB9	no
389	P	20	0.147	no	-	1.6	1.57	-	-	no
390	P	0	-	no	-	-	-	19	AC1	no
391	P	31	0.228	yes	no	1.6	0.63	19	AC1	no
392	E	51	0.263	yes	no	-3.5	0.04	19	AC1	no
393	L	0	-	no	-	-	-	19	AC1	no
394	L	12	0.073	no	-	3.8	-0.12	19	AC1	no
395	Q	114	0.576	yes	no	-3.5	-0.47	19	AC1	no
396	T	17	0.12	no	-	-0.7	0.14	19	AC1	no
397	L	0	-	no	-	-	-	19	AC1	no
398	T	12	0.085	no	-	-0.7	-0.77	19	AC1	no
399	A	33	0.311	yes	no	1.8	0.12	19	AC1	no
400	V	88	0.62	yes	no	4.2	1.2	-	-	yes
401	G	9	0.107	no	-	-0.4	-0.3	20	AC2	yes
402	G	1	-	no	-	-	-	20	AC2	no
403	I	13	0.077	no	-	4.5	0.74	20	AC2	no
404	G	35	0.417	yes	no	-0.4	-0.03	20	AC2	no
405	Q	139	0.702	yes	no	-3.5	0.8	20	AC2	no
406	L	117	0.713	yes	no	3.8	1.1	20	AC2	no
666	C	66	0.489	yes	no	2.5	-1	-	-	no
667	R	209	0.843	yes	no	-4.5	-0.35	-	-	no
668	I	38	0.225	yes	no	4.5	-1.07	-	-	no
669	K	103	0.502	yes	no	-3.9	-1.07	-	-	no
670	G	34	0.405	yes	no	-0.4	0.1	-	-	no
671	D	74	0.454	yes	no	-3.5	1.2	-	-	no

#	Residue	ACC	RSA	Surface	Interface	KD	KD mean	Helix number	Helix label	Calcium ion binding site
672	I	11	0.065	no	-	4.5	-0.97	-	-	no
673	G	2	-	no	-	-	-	-	-	no
674	Q	109	0.551	yes	no	-3.5	-2.12	-	-	no
675	S	7	0.054	no	-	-0.8	-2.34	-	-	no
676	T	76	0.535	yes	no	-0.7	-0.73	-	-	no
677	D	53	0.325	yes	no	-3.5	-2.07	-	-	yes
678	D	100	0.613	yes	no	-3.5	-2.34	-	-	yes
679	D	134	0.822	yes	no	-3.5	-2.6	-	-	no
680	S	33	0.254	yes	no	-0.8	-0.9	-	-	yes
681	A	3	-	no	-	-	-	21	AC3	no
682	P	2	-	no	-	-	-	21	AC3	no
683	L	0	-	no	-	-	-	21	AC3	no
684	V	8	0.056	no	-	4.2	0.5	21	AC3	no
685	H	16	0.087	no	-	-3.2	-0.1	21	AC3	no
686	C	0	-	no	-	-	-	21	AC3	no
687	V	0	-	no	-	-	-	21	AC3	no
688	R	21	0.085	no	-	-4.5	-1.3	21	AC3	no
689	L	15	0.091	no	-	3.8	-1.3	21	AC3	no
690	L	0	-	no	-	-	-	21	AC3	no
691	S	0	-	no	-	-	-	21	AC3	no
692	A	1	-	no	-	-	-	21	AC3	no
693	S	0	-	no	-	-	-	21	AC3	no
694	F	4	-	no	-	-	-	21	AC3	no
695	L	0	-	no	-	-	-	21	AC3	no
696	L	2	-	no	-	-	-	-	-	no
697	T	31	0.218	yes	no	-0.7	-0.55	-	-	no
698	G	3	-	no	-	-	-	-	-	yes
699	G	9	0.107	no	-	-0.4	-1.67	-	-	yes
700	K	110	0.537	yes	no	-3.9	-0.9	-	-	yes
701	N	84	0.535	yes	no	-3.5	-1.07	-	-	no
702	V	73	0.514	yes	no	4.2	0.15	-	-	no
703	L	47	0.287	yes	no	3.8	4	-	-	no
704	V	6	-	no	-	-	-	-	-	no

#	Residue	ACC	RSA	Surface	Interface	KD	KD mean	Helix number	Helix label	Calcium ion binding site
705	P	39	0.287	yes	no	1.6	-2.48	-	-	no
706	D	76	0.466	yes	no	-3.5	-1.7	-	-	no
707	R	221	0.891	yes	no	-4.5	-1.14	-	-	no
708	D	90	0.552	yes	no	-3.5	-1.14	-	-	no
709	V	12	0.085	no	-	4.2	-2.36	-	-	no
710	R	109	0.44	yes	no	-4.5	-0.08	22	AC4	no
711	V	45	0.317	yes	no	4.2	0.18	22	AC4	no
712	S	58	0.446	yes	no	-0.8	0.18	22	AC4	no
713	V	3	-	no	-	-	-	22	AC4	no
714	K	0	-	no	-	-	-	22	AC4	no
715	A	13	0.123	no	-	1.8	2.25	22	AC4	no
716	L	24	0.146	no	-	3.8	1.6	22	AC4	no
717	A	0	-	no	-	-	-	22	AC4	no
718	L	0	-	no	-	-	-	22	AC4	no
719	S	23	0.177	no	-	-0.8	1.1	22	AC4	no
720	C	0	-	no	-	-	-	22	AC4	no
721	V	0	-	no	-	-	-	22	AC4	no
722	G	5	0.06	no	-	-0.4	0.2	22	AC4	no
723	A	14	0.132	no	-	1.8	1.07	22	AC4	no
724	A	0	-	no	-	-	-	22	AC4	no
725	V	0	-	no	-	-	-	22	AC4	no
726	A	47	0.443	yes	no	1.8	0.45	22	AC4	no
727	L	30	0.183	no	-	3.8	0.8	22	AC4	no
728	H	19	0.103	no	-	-3.2	-0.28	22	AC4	no
729	P	0	-	no	-	-	-	23	AC5	no
730	E	35	0.18	no	-	-3.5	-3.35	23	AC5	no
731	S	0	-	no	-	-	-	23	AC5	no
732	F	0	-	no	-	-	-	23	AC5	no
733	F	1	-	no	-	-	-	23	AC5	no
734	S	11	0.085	no	-	-0.8	-1.8	23	AC5	no
735	K	55	0.268	yes	no	-3.9	-2	-	-	yes
736	L	0	-	no	-	-	-	-	-	no
737	Y	2	-	no	-	-	-	-	-	yes

#	Residue	ACC	RSA	Surface	Interface	KD	KD mean	Helix number	Helix label	Calcium ion binding site
738	K	66	0.322	yes	no	-3.9	1.6	-	-	yes
739	V	93	0.655	yes	no	4.2	-0.4	-	-	no
740	P	56	0.412	yes	no	1.6	1.43	-	-	no
741	L	16	0.098	no	-	3.8	0.16	-	-	yes
742	D	100	0.613	yes	no	-3.5	-0.28	-	-	yes
743	T	98	0.69	yes	no	-0.7	-0.61	-	-	no
744	T	139	0.979	yes	no	-0.7	-2.1	-	-	no
745	E	116	0.598	yes	no	-3.5	-0.92	-	-	yes
746	Y	42	0.189	no	-	-1.3	-1.48	-	-	yes
747	P	96	0.706	yes	no	1.6	-1.48	-	-	no
748	E	90	0.464	yes	no	-3.5	-1.68	-	-	yes
749	E	54	0.278	yes	no	-3.5	-3.5	-	-	yes
750	Q	1	-	no	-	-	-	-	-	no
751	Y	36	0.162	no	-	-1.3	-2.38	24	AC6	no
752	V	0	-	no	-	-	-	24	AC6	no
753	S	10	0.077	no	-	-0.8	-0.45	24	AC6	no
754	D	26	0.16	no	-	-3.5	-1.06	24	AC6	yes
755	I	0	-	no	-	-	-	24	AC6	no
756	L	14	0.085	no	-	3.8	0.1	24	AC6	no
757	N	55	0.35	yes	no	-3.5	0.32	24	AC6	no
758	Y	0	-	no	-	-	-	24	AC6	no
759	I	10	0.059	no	-	4.5	-1.07	24	AC6	no
760	D	110	0.675	yes	no	-3.5	-0.65	24	AC6	no
761	H	44	0.239	yes	no	-3.2	-0.65	24	AC6	no
762	G	61	0.726	yes	no	-0.4	-2.65	-	-	no
763	D	40	0.245	yes	no	-3.5	-0.77	-	-	no
764	P	47	0.346	yes	no	1.6	-2.48	-	-	no
765	Q	95	0.48	yes	no	-3.5	-1.15	-	-	no
766	V	0	-	no	-	-	-	25	AC7	no
767	R	14	0.056	no	-	-4.5	-0.02	25	AC7	yes
768	G	0	-	no	-	-	-	25	AC7	no
769	A	9	0.085	no	-	1.8	-0.5	25	AC7	no
770	T	0	-	no	-	-	-	25	AC7	no

#	Residue	ACC	RSA	Surface	Interface	KD	KD mean	Helix number	Helix label	Calcium ion binding site
771	A	0	-	no	-	-	-	25	AC7	no
772	I	38	0.225	yes	no	4.5	3.15	25	AC7	no
773	L	1	-	no	-	-	-	25	AC7	no
774	C	0	-	no	-	-	-	25	AC7	no
775	G	0	-	no	-	-	-	25	AC7	no
776	T	24	0.169	no	-	-0.7	0.7	25	AC7	no
777	L	0	-	no	-	-	-	25	AC7	no
778	I	0	-	no	-	-	-	25	AC7	no
779	C	42	0.311	yes	no	2.5	0.05	25	AC7	no
780	S	12	0.092	no	-	-0.8	-0.42	25	AC7	no
781	I	0	-	no	-	-	-	25	AC7	no
782	L	1	-	no	-	-	-	25	AC7	no
783	S	47	0.362	yes	no	-0.8	-2.28	25	AC7	no
784	R	73	0.294	yes	no	-4.5	-1.72	25	AC7	no
785	S	10	0.077	no	-	-0.8	-1.7	25	AC7	no
786	R	30	0.121	no	-	-4.5	-1.3	-	-	no
787	F	30	0.152	no	-	2.8	-1.43	-	-	no
788	H	33	0.179	no	-	-3.2	-1.6	26	AC8	no
789	V	2	-	no	-	-	-	26	AC8	no
790	G	56	0.667	yes	no	-0.4	-1.22	26	AC8	no
791	D	120	0.736	yes	no	-3.5	-1.08	26	AC8	no
792	W	37	0.163	no	-	-0.9	-0.69	26	AC8	no
793	M	25	0.133	no	-	1.9	-0.5	26	AC8	no
794	G	33	0.393	yes	no	-0.4	-0.51	26	AC8	no
795	T	86	0.606	yes	no	-0.7	-0.11	26	AC8	no
796	I	10	0.059	no	-	4.5	0.4	26	AC8	no
797	R	138	0.556	yes	no	-4.5	-0.07	26	AC8	no
798	T	121	0.852	yes	no	-0.7	0.19	26	AC8	no
799	L	131	0.799	yes	no	3.8	0.33	26	AC8	no
800	T	30	0.211	yes	no	-0.7	0.17	26	AC8	no
801	G	40	0.476	yes	no	-0.4	-1	-	-	no
802	N	10	0.064	no	-	-3.5	-1.26	-	-	no
803	T	100	0.704	yes	no	-0.7	-0.47	-	-	no

#	Residue	ACC	RSA	Surface	Interface	KD	KD mean	Helix number	Helix label	Calcium ion binding site
804	F	30	0.152	no	-	2.8	-0.55	-	-	no
805	S	45	0.346	yes	no	-0.8	0.07	27	AC9	no
806	L	1	-	no	-	-	-	27	AC9	no
807	A	32	0.302	yes	no	1.8	-0.83	27	AC9	no
808	D	72	0.442	yes	no	-3.5	-0.22	27	AC9	no
809	C	0	-	no	-	-	-	28	AD1	no
810	I	4	-	no	-	-	-	28	AD1	no
811	P	70	0.515	yes	no	1.6	-0.65	28	AD1	no
812	L	29	0.177	no	-	3.8	-0.75	28	AD1	no
813	L	0	-	no	-	-	-	28	AD1	no
814	R	80	0.323	yes	no	-4.5	0.16	28	AD1	no
815	K	93	0.454	yes	no	-3.9	-0.94	28	AD1	no
816	T	1	-	no	-	-	-	28	AD1	no
817	L	9	0.055	no	-	3.8	-2.4	28	AD1	no
818	K	143	0.698	yes	no	-3.9	-1.88	28	AD1	no
819	D	41	0.252	yes	no	-3.5	-1.58	-	-	yes
820	E	176	0.907	yes	no	-3.5	-2.6	-	-	yes
821	S	28	0.215	yes	no	-0.8	-0.88	29	AD2	no
822	S	44	0.338	yes	no	-0.8	0.87	29	AD2	no
823	V	60	0.423	yes	no	4.2	-0.32	29	AD2	no
824	T	0	-	no	-	-	-	29	AD2	no
825	C	6	-	no	-	-	-	29	AD2	no
826	K	50	0.244	yes	no	-3.9	1.37	29	AD2	no
827	L	26	0.159	no	-	3.8	-0.27	29	AD2	no
828	A	0	-	no	-	-	-	29	AD2	no
829	C	0	-	no	-	-	-	29	AD2	no
830	T	28	0.197	no	-	-0.7	-0.47	29	AD2	no
831	A	0	-	no	-	-	-	29	AD2	no
832	V	0	-	no	-	-	-	29	AD2	no
833	R	70	0.282	yes	no	-4.5	-2.9	29	AD2	no
834	N	70	0.446	yes	no	-3.5	-4	29	AD2	no
835	C	0	-	no	-	-	-	29	AD2	no
836	V	1	-	no	-	-	-	29	AD2	no

#	Residue	ACC	RSA	Surface	Interface	KD	KD mean	Helix number	Helix label	Calcium ion binding site
837	M	40	0.213	yes	no	1.9	0.55	29	AD2	no
838	S	22	0.169	no	-	-0.8	0.7	29	AD2	no
839	L	0	-	no	-	-	-	29	AD2	no
840	C	0	-	no	-	-	-	29	AD2	no
841	S	37	0.285	yes	no	-0.8	0.1	29	AD2	no
842	S	0	-	no	-	-	-	-	-	no
843	S	25	0.192	no	-	-0.8	-0.15	-	-	no
844	Y	51	0.23	yes	no	-1.3	-0.45	30	AD3	no
845	S	0	-	no	-	-	-	30	AD3	no
846	E	36	0.186	no	-	-3.5	0.7	30	AD3	no
847	L	37	0.226	yes	no	3.8	-1.12	30	AD3	no
848	G	0	-	no	-	-	-	30	AD3	no
849	L	1	-	no	-	-	-	30	AD3	no
850	Q	38	0.192	no	-	-3.5	0.15	30	AD3	no
851	L	0	-	no	-	-	-	30	AD3	no
852	I	0	-	no	-	-	-	30	AD3	no
853	I	8	-	no	-	-	-	30	AD3	no
854	D	23	0.141	no	-	-3.5	-1.95	30	AD3	no
855	V	0	-	no	-	-	-	30	AD3	no
856	L	1	-	no	-	-	-	30	AD3	no
857	T	66	0.465	yes	no	-0.7	-0.47	31	AD4	no
858	L	9	0.055	no	-	3.8	-1.14	31	AD4	no
859	R	22	0.089	no	-	-4.5	-1.7	31	AD4	no
860	N	70	0.446	yes	no	-3.5	-1.25	31	AD4	no
861	S	23	0.177	no	-	-0.8	-1.18	31	AD4	no
862	S	122	0.938	yes	yes	-0.8	-0.97	-	-	no
863	Y	78	0.351	yes	yes	-1.3	0	32	AD5	no
864	W	77	0.339	yes	yes	-0.9	-0.73	32	AD5	no
865	L	47	0.287	yes	yes	3.8	-1.28	32	AD5	no
866	V	0	-	no	-	-	-	32	AD5	no
867	R	28	0.113	no	-	-4.5	-1.92	32	AD5	yes
868	T	4	-	no	-	-	-	32	AD5	no
869	E	11	0.057	no	-	-3.5	-1.93	32	AD5	no

#	Residue	ACC	RSA	Surface	Interface	KD	KD mean	Helix number	Helix label	Calcium ion binding site
870	L	0	-	no	-	-	-	32	AD5	no
871	L	0	-	no	-	-	-	32	AD5	no
872	E	53	0.273	yes	yes	-3.5	-3.5	32	AD5	no
873	T	0	-	no	-	-	-	32	AD5	no
874	L	0	-	no	-	-	-	32	AD5	no
875	A	1	-	no	-	-	-	32	AD5	no
876	E	61	0.314	yes	no	-3.5	-3.5	32	AD5	no
877	I	0	-	no	-	-	-	-	-	no
878	D	21	0.129	no	-	-3.5	-3.97	33	AD6	no
879	F	1	-	no	-	-	-	33	AD6	no
880	R	32	0.129	no	-	-4.5	-0.88	33	AD6	no
881	L	7	-	no	-	-	-	33	AD6	no
882	V	0	-	no	-	-	-	33	AD6	no
883	S	19	0.146	no	-	-0.8	-2.93	33	AD6	no
884	F	5	-	no	-	-	-	33	AD6	no
885	L	0	-	no	-	-	-	33	AD6	no
886	E	24	0.124	no	-	-3.5	-0.92	33	AD6	no
887	A	80	0.755	yes	no	1.8	-0.95	33	AD6	no
888	K	85	0.415	yes	no	-3.9	-0.95	33	AD6	no
889	A	7	0.066	no	-	1.8	-0.58	33	AD6	no
890	E	167	0.861	yes	yes	-3.5	-0.35	-	-	no
891	N	130	0.828	yes	no	-3.5	-2.18	-	-	no
892	L	46	0.28	yes	no	3.8	-1.36	-	-	no
893	H	17	0.092	no	-	-3.2	-0.67	-	-	no
894	R	34	0.137	no	-	-4.5	-1.76	-	-	no
895	G	35	0.417	yes	no	-0.4	-0.5	34	AD7	no
896	A	77	0.726	yes	no	1.8	-1.25	34	AD7	no
897	H	38	0.207	yes	no	-3.2	-1.48	34	AD7	no
898	H	44	0.239	yes	yes	-3.2	-2.08	34	AD7	no
899	Y	28	0.126	no	-	-1.3	-2.58	34	AD7	no
900	T	9	0.183	no	-	-0.7	1.26	-	-	no
901	G	15	0.536	yes	yes	-0.4	0.9	-	-	no
902	L	59	0.616	yes	yes	3.8	-0.93	-	-	no

#	Residue	ACC	RSA	Surface	Interface	KD	KD mean	Helix number	Helix label	Calcium ion binding site
903	L	0	-	no	-	-	-	-	-	no
904	K	70	0.507	yes	no	-3.9	-1.78	35	AD8	no
905	L	0	-	no	-	-	-	35	AD8	no
906	Q	18	0.091	no	-	-3.5	-3.63	35	AD8	no
907	E	101	0.521	yes	no	-3.5	-3.6	35	AD8	no
908	R	5	-	no	-	-	-	35	AD8	no
909	V	0	-	no	-	-	-	35	AD8	no
910	L	3	-	no	-	-	-	35	AD8	no
911	N	71	0.452	yes	no	-3.5	-3.5	35	AD8	no
912	N	62	0.395	yes	no	-3.5	-3.4	35	AD8	no
913	V	1	-	no	-	-	-	35	AD8	no
914	V	0	-	no	-	-	-	-	-	no
915	I	2	-	no	-	-	-	36	AD9	no
916	H	95	0.516	yes	no	-3.2	-2.37	36	AD9	no
917	L	1	-	no	-	-	-	36	AD9	no
918	L	0	-	no	-	-	-	36	AD9	no
919	G	20	0.238	yes	no	-0.4	-0.83	36	AD9	no
920	D	21	0.129	no	-	-3.5	-3.08	36	AD9	yes
921	E	138	0.711	yes	no	-3.5	-3.5	-	-	no
922	D	28	0.172	no	-	-3.5	-2.68	37	AE1	yes
923	P	75	0.551	yes	no	1.6	-2.73	37	AE1	no
924	R	122	0.492	yes	yes	-4.5	-2.82	37	AE1	no
925	V	0	-	no	-	-	-	37	AE1	no
926	R	26	0.105	no	-	-4.5	-1.65	37	AE1	no
927	H	98	0.533	yes	yes	-3.2	-2	37	AE1	no
928	V	22	0.155	no	-	4.2	-0.43	37	AE1	no
929	A	0	-	no	-	-	-	37	AE1	no
930	A	0	-	no	-	-	-	37	AE1	no
931	A	38	0.358	yes	no	1.8	3.5	37	AE1	no
932	S	2	-	no	-	-	-	37	AE1	no
933	L	1	-	no	-	-	-	37	AE1	no
934	I	34	0.201	yes	no	4.5	0.6	37	AE1	no
935	R	120	0.484	yes	no	-4.5	0	37	AE1	no

#	Residue	ACC	RSA	Surface	Interface	KD	KD mean	Helix number	Helix label	Calcium ion binding site
936	L	1	-	no	-	-	-	37	AE1	no
937	V	0	-	no	-	-	-	37	AE1	no
938	P	45	0.331	yes	no	1.6	-1.15	38	AE2	no
939	K	28	0.137	no	-	-3.9	-1.93	38	AE2	no
940	L	1	-	no	-	-	-	38	AE2	no
941	F	14	0.071	no	-	2.8	-1	-	-	yes
942	Y	29	0.144	no	-	-1.3	-0.8	-	-	yes
943	K	104	0.629	yes	no	-3.9	-1.55	-	-	no
944	C	51	0.844	yes	yes	2.5	-0.78	-	-	no
945	D	9	0.055	no	-	-3.5	-1.32	-	-	yes
946	Q	91	0.652	yes	no	-3.5	-3.5	-	-	no
947	G	4	-	no	-	-	-	-	-	no
948	Q	5	-	no	-	-	-	-	-	yes
949	A	45	0.425	yes	no	1.8	-0.68	-	-	no
950	D	31	0.19	no	-	-3.5	-0.03	39	AE3	no
951	P	17	0.125	no	-	1.6	-2.13	39	AE3	no
952	V	3	-	no	-	-	-	39	AE3	no
953	V	3	-	no	-	-	-	39	AE3	no
954	A	0	-	no	-	-	-	39	AE3	no
955	V	5	-	no	-	-	-	39	AE3	no
956	A	0	-	no	-	-	-	39	AE3	no
957	R	44	0.177	no	-	-4.5	-2.62	39	AE3	no
958	D	65	0.399	yes	no	-3.5	-2.62	39	AE3	no
959	Q	30	0.152	no	-	-3.5	-2.62	39	AE3	no
960	S	7	0.054	no	-	-0.8	-0.8	39	AE3	no
961	S	74	0.569	yes	no	-0.8	-1.12	39	AE3	no
962	V	51	0.359	yes	no	4.2	0.2	39	AE3	no
963	Y	34	0.153	no	-	-1.3	-0.25	39	AE3	no
964	L	25	0.152	no	-	3.8	0.2	39	AE3	no
965	K	119	0.58	yes	no	-3.9	0.97	-	-	no
966	L	68	0.415	yes	no	3.8	1.4	-	-	no
967	L	24	0.146	no	-	3.8	3.17	-	-	no
968	M	58	0.309	yes	no	1.9	1.57	-	-	no

#	Residue	ACC	RSA	Surface	Interface	KD	KD mean	Helix number	Helix label	Calcium ion binding site
969	H	39	0.212	yes	no	-3.2	-1.6	-	-	no
970	E	170	0.876	yes	no	-3.5	-3.35	-	-	no
983	T	142	1	yes	yes	-0.7	-2.6	-	-	no
984	R	138	0.556	yes	yes	-4.5	-0.23	-	-	yes
985	I	79	0.467	yes	yes	4.5	-0.43	-	-	no
986	Y	26	0.117	no	-	-1.3	-0.43	-	-	no
987	R	105	0.423	yes	yes	-4.5	-2.9	-	-	no
988	G	4	-	no	-	-	-	-	-	no
989	Y	10	-	no	-	-	-	-	-	no
990	N	27	0.172	no	-	-3.5	1.65	-	-	no
991	L	69	0.421	yes	yes	3.8	1.42	-	-	no
992	L	123	0.75	yes	yes	3.8	0.98	-	-	no
993	P	121	0.89	yes	yes	1.6	2.28	-	-	no
994	S	35	0.269	yes	yes	-0.8	2.15	-	-	no
995	I	172	1	yes	yes	4.5	1.15	-	-	no
996	T	40	0.282	yes	yes	-0.7	-0.13	-	-	no
997	D	50	0.307	yes	no	-3.5	0.47	40	AE4	no
998	V	53	0.373	yes	yes	4.2	-0.22	40	AE4	no
999	T	5	-	no	-	-	-	40	AE4	no
1000	M	45	0.239	yes	yes	1.9	-0.88	40	AE4	no
1001	E	9	0.052	no	-	-3.5	-0.22	40	AE4	no
1002	N	10	0.268	yes	no	-3.5	-1.48	40	AE4	no
1003	N	3	-	no	-	-	-	40	AE4	no
1004	L	0	-	no	-	-	-	40	AE4	no
1005	S	3	0.385	yes	yes	-0.8	-2.93	40	AE4	no
1006	R	19	0.403	yes	no	-4.5	-1.17	40	AE4	no
1007	V	0	-	no	-	-	-	40	AE4	no
1008	I	5	-	no	-	-	-	40	AE4	no
1009	A	14	0.566	yes	yes	1.8	-0.42	40	AE4	no
1010	A	21	0.198	no	-	1.8	-0.1	40	AE4	no
1011	V	0	-	no	-	-	-	40	AE4	no
1012	S	10	0.085	no	-	-0.8	-0.02	40	AE4	no
1013	H	47	0.587	yes	yes	-3.2	0.43	40	AE4	no

#	Residue	ACC	RSA	Surface	Interface	KD	KD mean	Helix number	Helix label	Calcium ion binding site
1014	E	66	0.34	yes	yes	-3.5	-0.1	40	AE4	no
1015	L	11	0.067	no	-	3.8	-0.1	40	AE4	no
1016	I	23	0.645	yes	yes	4.5	0.02	40	AE4	no
1017	T	45	0.493	yes	yes	-0.7	0.66	40	AE4	no
1018	S	13	0.185	no	-	-0.8	0.27	-	-	no
1019	T	73	0.965	yes	yes	-0.7	-0.73	-	-	no
1020	T	54	0.387	yes	no	-0.7	-0.58	41	AE5	no
1021	R	104	0.589	yes	no	-4.5	-0.47	41	AE5	no
1022	A	3	-	no	-	-	-	41	AE5	no
1023	L	19	0.116	no	-	3.8	0.2	41	AE5	no
1024	T	6	-	no	-	-	-	41	AE5	no
1025	F	20	0.102	no	-	2.8	3.3	41	AE5	no
1026	G	0	-	no	-	-	-	41	AE5	no
1027	C	0	-	no	-	-	-	41	AE5	no
1028	C	0	-	no	-	-	-	41	AE5	no
1029	E	36	0.186	no	-	-3.5	-3.5	41	AE5	no
1030	A	0	-	no	-	-	-	41	AE5	no
1031	L	0	-	no	-	-	-	41	AE5	no
1032	C	4	-	no	-	-	-	41	AE5	no
1033	L	36	0.22	yes	yes	3.8	1.55	41	AE5	no
1034	L	0	-	no	-	-	-	41	AE5	no
1035	S	3	-	no	-	-	-	41	AE5	no
1036	T	93	0.655	yes	no	-0.7	1.86	41	AE5	no
1037	A	49	0.462	yes	yes	1.8	1.38	41	AE5	no
1038	F	17	0.086	no	-	2.8	1.38	41	AE5	no
1039	P	35	0.257	yes	yes	1.6	1.66	-	-	no
1040	V	3	-	no	-	-	-	-	-	no
1041	C	10	0.074	no	-	2.5	0.74	-	-	no
1042	I	64	0.379	yes	yes	4.5	1.02	-	-	no
1043	W	34	0.15	no	-	-0.9	1.78	-	-	no
1044	S	21	0.162	no	-	-0.8	1.93	-	-	no
1045	L	83	0.72	yes	no	3.8	-0.28	-	-	no
1046	G	1	-	no	-	-	-	-	-	no

#	Residue	ACC	RSA	Surface	Interface	KD	KD mean	Helix number	Helix label	Calcium ion binding site
1047	W	0	-	no	-	-	-	-	-	no
1048	H	35	0.793	yes	no	-3.2	1.03	-	-	no
1049	C	51	0.526	yes	yes	2.5	0.6	-	-	no
1064	C	31	0.23	yes	no	2.5	1.5	-	-	no
1065	T	5	-	no	-	-	-	-	-	no
1066	V	52	0.415	yes	no	4.2	1.9	42	AE6	no
1067	G	8	0.524	yes	yes	-0.4	1.25	42	AE6	no
1068	M	14	0.096	no	-	1.9	0.68	42	AE6	no
1069	A	0	-	no	-	-	-	42	AE6	no
1070	T	36	0.423	yes	yes	-0.7	0.67	42	AE6	no
1071	M	15	0.234	yes	yes	1.9	0.6	42	AE6	no
1072	I	0	-	no	-	-	-	42	AE6	no
1073	L	2	-	no	-	-	-	42	AE6	no
1074	T	16	0.634	yes	yes	-0.7	1.05	42	AE6	no
1075	L	24	0.146	no	-	3.8	0.37	42	AE6	no
1076	L	6	-	no	-	-	-	42	AE6	no
1077	S	43	0.608	yes	yes	-0.8	0.38	42	AE6	no
1078	S	21	0.185	no	-	-0.8	0.62	-	-	no
1079	A	14	0.585	yes	no	1.8	0.03	-	-	no
1080	W	45	0.379	yes	no	-0.9	1.1	43	AE7	no
1081	F	0	-	no	-	-	-	43	AE7	no
1082	P	14	0.103	no	-	1.6	0.25	43	AE7	no
1083	L	49	0.299	yes	no	3.8	0.96	43	AE7	no
1084	D	49	0.301	yes	no	-3.5	0.98	44	AE8	no
1085	L	9	0.055	no	-	3.8	0.82	44	AE8	no
1086	S	42	0.323	yes	no	-0.8	-1	44	AE8	no
1087	A	3	-	no	-	-	-	44	AE8	no
1088	H	0	-	no	-	-	-	44	AE8	no
1089	Q	31	0.157	no	-	-3.5	-2.15	44	AE8	no
1090	D	24	0.147	no	-	-3.5	-1.5	44	AE8	no
1091	A	0	-	no	-	-	-	44	AE8	no
1092	L	0	-	no	-	-	-	44	AE8	no
1093	I	23	0.136	no	-	4.5	0.5	44	AE8	no

#	Residue	ACC	RSA	Surface	Interface	KD	KD mean	Helix number	Helix label	Calcium ion binding site
1094	L	0	-	no	-	-	-	44	AE8	no
1095	A	0	-	no	-	-	-	44	AE8	no
1096	G	0	-	no	-	-	-	44	AE8	no
1097	N	10	0.064	no	-	-3.5	-3.5	44	AE8	no
1098	L	0	-	no	-	-	-	44	AE8	no
1099	L	1	-	no	-	-	-	44	AE8	no
1100	A	0	-	no	-	-	-	44	AE8	no
1101	A	6	0.057	no	-	1.8	2.27	44	AE8	no
1102	S	6	-	no	-	-	-	45	AE9	no
1103	A	0	-	no	-	-	-	45	AE9	no
1104	P	55	0.404	yes	no	1.6	0.17	45	AE9	no
1105	K	160	0.78	yes	no	-3.9	-1.03	45	AE9	no
1106	S	7	0.054	no	-	-0.8	-0.56	45	AE9	no
1107	L	23	0.14	no	-	3.8	-0.02	-	-	no
1108	R	95	0.383	yes	no	-4.5	-0.02	-	-	no
1109	S	122	0.938	yes	no	-0.8	-1.5	-	-	no
1126	E	147	0.758	yes	no	-3.5	0.77	-	-	no
1127	V	69	0.486	yes	no	4.2	0.77	-	-	no
1128	W	8	-	no	-	-	-	-	-	no
1129	P	35	0.257	yes	no	1.6	1.03	-	-	no
1130	A	8	0.075	no	-	1.8	0.9	-	-	no
1131	L	1	-	no	-	-	-	-	-	no
1132	G	27	0.321	yes	no	-0.4	-1.82	-	-	no
1133	D	68	0.417	yes	no	-3.5	-1.48	-	-	no
1134	R	84	0.339	yes	no	-4.5	-1.65	-	-	no
1135	A	56	0.528	yes	no	1.8	-2.07	-	-	no
1136	L	3	-	no	-	-	-	46	AF1	no
1137	V	6	-	no	-	-	-	46	AF1	no
1138	P	75	0.551	yes	no	1.6	-0.88	46	AF1	no
1139	M	13	0.069	no	-	1.9	-0.88	46	AF1	no
1140	V	2	-	no	-	-	-	46	AF1	no
1141	E	81	0.418	yes	no	-3.5	-0.88	46	AF1	no
1142	Q	61	0.348	yes	no	-3.5	-0.86	46	AF1	no

#	Residue	ACC	RSA	Surface	Interface	KD	KD mean	Helix number	Helix label	Calcium ion binding site
1143	L	0	-	no	-	-	-	46	AF1	no
1144	F	9	-	no	-	-	-	46	AF1	no
1145	S	38	0.292	yes	no	-0.8	-2.15	46	AF1	no
1146	H	9	-	no	-	-	-	46	AF1	no
1147	L	0	-	no	-	-	-	46	AF1	no
1148	L	1	-	no	-	-	-	46	AF1	no
1149	K	26	0.127	no	-	-3.9	-2.73	46	AF1	no
1150	V	0	-	no	-	-	-	46	AF1	no
1151	I	3	-	no	-	-	-	46	AF1	no
1152	N	11	0.07	no	-	-3.5	-1.87	46	AF1	no
1153	I	0	-	no	-	-	-	46	AF1	no
1154	C	0	-	no	-	-	-	46	AF1	no
1155	A	7	0.066	no	-	1.8	-0.31	46	AF1	no
1156	H	24	0.13	no	-	-3.2	0.54	46	AF1	no
1157	V	8	0.056	no	-	4.2	-0.61	46	AF1	no
1158	L	24	0.146	no	-	3.8	-0.07	46	AF1	no
1159	D	81	0.497	yes	no	-3.5	0.54	46	AF1	no
1160	D	123	0.755	yes	no	-3.5	0.33	-	-	no
1161	V	77	0.542	yes	no	4.2	-0.84	-	-	no
1162	A	78	0.736	yes	no	1.8	2.53	-	-	no
1163	P	31	0.228	yes	no	1.6	0.42	-	-	no
1164	G	92	1	yes	yes	-0.4	-0.03	-	-	no
1228	S	143	1	yes	yes	-0.8	0.73	-	-	no
1229	L	69	0.421	yes	yes	3.8	0.73	-	-	no
1230	G	0	-	no	-	-	-	-	-	no
1231	S	37	0.285	yes	yes	-0.8	-0.42	-	-	no
1232	F	0	-	no	-	-	-	-	-	no
1233	Y	92	0.414	yes	yes	-1.3	0.1	-	-	no
1234	H	145	0.788	yes	yes	-3.2	-0.23	-	-	no
1235	L	45	0.274	yes	no	3.8	-0.2	47	AF2	no
1236	P	90	0.662	yes	yes	1.6	1.42	47	AF2	no
1237	S	36	0.431	yes	yes	-0.8	0.53	47	AF2	no
1238	Y	38	0.171	no	-	-1.3	0.53	47	AF2	no

#	Residue	ACC	RSA	Surface	Interface	KD	KD mean	Helix number	Helix label	Calcium ion binding site
1239	L	57	0.348	yes	no	3.8	-0.12	47	AF2	no
1240	K	93	0.454	yes	no	-3.9	-1.14	47	AF2	no
1241	L	2	-	no	-	-	-	47	AF2	no
1242	H	7	-	no	-	-	-	47	AF2	no
1243	D	100	0.613	yes	no	-3.5	-1.77	47	AF2	no
1244	V	44	0.31	yes	no	4.2	-0.35	47	AF2	no
1245	L	0	-	no	-	-	-	47	AF2	no
1246	K	122	0.595	yes	no	-3.9	0.09	47	AF2	no
1247	A	64	0.604	yes	no	1.8	0	47	AF2	no
1248	T	40	0.282	yes	no	-0.7	-1.28	47	AF2	no
1249	H	17	0.092	no	-	-3.2	-1.29	47	AF2	no
1250	A	52	0.491	yes	no	1.8	-1.61	47	AF2	no
1251	N	116	0.739	yes	no	-3.5	-0.94	47	AF2	no
1252	Y	46	0.207	yes	no	-1.3	-0.94	47	AF2	no
1253	K	60	0.293	yes	no	-3.9	-1.04	47	AF2	no
1254	V	29	0.204	yes	no	4.2	-2.01	-	-	no
1255	T	46	0.324	yes	no	-0.7	-1.04	-	-	no
1256	L	1	-	no	-	-	-	-	-	no
1257	D	74	0.454	yes	no	-3.5	-1.68	-	-	yes
1258	L	37	0.226	yes	no	3.8	-1.68	-	-	no
1259	Q	121	0.611	yes	no	-3.5	-1.5	-	-	no
1260	N	67	0.427	yes	no	-3.5	-1.37	-	-	no
1261	S	92	0.708	yes	no	-0.8	-2.4	-	-	no
1262	T	114	0.803	yes	no	-0.7	-2.12	-	-	no
1263	E	30	0.155	no	-	-3.5	-2.23	48	AF3	yes
1264	K	99	0.483	yes	no	-3.9	-3.7	48	AF3	no
1265	F	1	-	no	-	-	-	48	AF3	no
1266	G	0	-	no	-	-	-	48	AF3	no
1267	G	3	-	no	-	-	-	48	AF3	no
1268	F	0	-	no	-	-	-	48	AF3	no
1269	L	0	-	no	-	-	-	48	AF3	no
1270	R	91	0.367	yes	no	-4.5	-2.65	48	AF3	no
1271	S	13	0.1	no	-	-0.8	-3.08	48	AF3	no

#	Residue	ACC	RSA	Surface	Interface	KD	KD mean	Helix number	Helix label	Calcium ion binding site
1272	A	0	-	no	-	-	-	48	AF3	no
1273	L	0	-	no	-	-	-	48	AF3	no
1274	D	33	0.202	yes	no	-3.5	-2.15	48	AF3	no
1275	V	0	-	no	-	-	-	48	AF3	no
1276	L	2	-	no	-	-	-	48	AF3	no
1277	S	0	-	no	-	-	-	48	AF3	no
1278	Q	0	-	no	-	-	-	48	AF3	no
1279	I	0	-	no	-	-	-	48	AF3	no
1280	L	0	-	no	-	-	-	48	AF3	no
1281	E	2	-	no	-	-	-	49	AF4	yes
1282	L	2	-	no	-	-	-	49	AF4	no
1283	A	0	-	no	-	-	-	49	AF4	no
1284	T	24	0.169	no	-	-0.7	-0.98	50	AF5	no
1285	L	65	0.396	yes	no	3.8	-0.08	50	AF5	no
1286	Q	144	0.727	yes	no	-3.5	-0.86	50	AF5	no
1287	D	23	0.141	no	-	-3.5	-0.98	50	AF5	no
1288	I	0	-	no	-	-	-	50	AF5	no
1289	G	16	0.19	no	-	-0.4	-1	50	AF5	no
1290	K	175	0.854	yes	yes	-3.9	-2.15	-	-	no
1291	C	2	-	no	-	-	-	51	AF6	no
1292	V	0	-	no	-	-	-	51	AF6	no
1293	E	85	0.438	yes	no	-3.5	-3.5	51	AF6	no
1294	E	29	0.149	no	-	-3.5	-2.47	51	AF6	no
1295	I	2	-	no	-	-	-	51	AF6	no
1296	L	7	-	no	-	-	-	51	AF6	no
1297	G	42	0.5	yes	no	-0.4	-2.27	51	AF6	no
1298	Y	30	0.135	no	-	-1.3	-0.92	51	AF6	no
1299	L	1	-	no	-	-	-	51	AF6	no
1300	K	90	0.439	yes	no	-3.9	-1.6	51	AF6	no
1301	S	7	0.054	no	-	-0.8	-1.05	51	AF6	no
1302	C	0	-	no	-	-	-	51	AF6	no
1303	F	9	-	no	-	-	-	51	AF6	no
1304	S	87	0.669	yes	no	-0.8	-1.8	-	-	no

#	Residue	ACC	RSA	Surface	Interface	KD	KD mean	Helix number	Helix label	Calcium ion binding site
1305	R	31	0.125	no	-	-4.5	-2.93	-	-	no
1306	E	20	0.103	no	-	-3.5	-0.57	52	AF7	no
1307	P	23	0.169	no	-	1.6	0.22	52	AF7	no
1308	M	63	0.335	yes	no	1.9	0.48	52	AF7	no
1309	M	12	0.064	no	-	1.9	1.22	52	AF7	no
1310	A	1	-	no	-	-	-	52	AF7	no
1311	T	2	-	no	-	-	-	52	AF7	no
1312	V	12	0.085	no	-	4.2	0.87	52	AF7	no
1313	C	0	-	no	-	-	-	52	AF7	no
1314	V	5	-	no	-	-	-	52	AF7	no
1315	Q	15	0.076	no	-	-3.5	0.35	52	AF7	no
1316	Q	9	-	no	-	-	-	52	AF7	no
1317	L	0	-	no	-	-	-	52	AF7	no
1318	L	0	-	no	-	-	-	52	AF7	no
1319	K	15	0.073	no	-	-3.9	0.32	52	AF7	yes
1320	T	1	-	no	-	-	-	52	AF7	no
1321	L	22	0.134	no	-	3.8	1.22	52	AF7	no
1322	F	81	0.411	yes	yes	2.8	0.32	52	AF7	no
1323	G	28	0.333	yes	yes	-0.4	0.13	-	-	no
1324	T	34	0.239	yes	yes	-0.7	-0.55	-	-	no
1325	N	3	-	no	-	-	-	-	-	no
1326	L	9	0.055	no	-	3.8	0.32	-	-	no
1327	A	9	0.085	no	-	1.8	0.98	-	-	no
1328	S	42	0.323	yes	no	-0.8	0.62	-	-	no
1329	Q	118	0.596	yes	yes	-3.5	0.82	-	-	no
1330	F	51	0.259	yes	yes	2.8	-0.67	-	-	no
1331	D	168	1	yes	no	-3.5	-0.5	-	-	no
1353	R	272	1	yes	yes	-4.5	-1.45	-	-	no
1354	P	37	0.272	yes	no	1.6	-1.62	-	-	no
1355	G	9	0.107	no	-	-0.4	-0.82	-	-	no
1356	L	3	-	no	-	-	-	-	-	no
1357	Y	23	0.104	no	-	-1.3	-0.86	-	-	no
1358	H	77	0.418	yes	yes	-3.2	-0.13	-	-	no

#	Residue	ACC	RSA	Surface	Interface	KD	KD mean	Helix number	Helix label	Calcium ion binding site
1359	Y	40	0.18	no	-	-1.3	-1	-	-	no
1360	C	6	-	no	-	-	-	-	-	no
1361	F	6	-	no	-	-	-	53	AF8	no
1362	M	73	0.388	yes	yes	1.9	-0.17	53	AF8	no
1363	A	22	0.208	yes	no	1.8	-0.17	53	AF8	no
1364	P	22	0.162	no	-	1.6	0.02	53	AF8	no
1365	Y	22	0.099	no	-	-1.3	0.41	53	AF8	no
1366	T	40	0.282	yes	no	-0.7	-0.14	53	AF8	no
1367	H	100	0.543	yes	no	-3.2	-0.71	53	AF8	no
1368	F	22	0.112	no	-	2.8	-0.69	53	AF8	no
1369	T	32	0.225	yes	no	-0.7	-0.24	53	AF8	no
1370	Q	106	0.535	yes	yes	-3.5	-0.24	53	AF8	no
1371	A	14	0.132	no	-	1.8	-0.22	53	AF8	no
1372	L	6	-	no	-	-	-	53	AF8	no
1373	A	66	0.623	yes	no	1.8	-0.82	53	AF8	no
1374	D	108	0.663	yes	yes	-3.5	0.62	53	AF8	no
1375	A	3	-	no	-	-	-	53	AF8	no
1376	S	84	0.646	yes	yes	-0.8	-0.17	54	AF9	no
1377	L	185	1	yes	yes	3.8	-0.17	54	AF9	no
1423	H	126	0.685	yes	yes	-3.2	0.15	55	AG1	no
1424	I	53	0.314	yes	yes	4.5	0.15	55	AG1	no
1425	R	214	0.863	yes	yes	-4.5	-0.58	55	AG1	no
1426	L	66	0.402	yes	yes	3.8	-0.58	55	AG1	no
1427	F	1	-	no	-	-	-	56	AG2	no
1428	E	83	0.428	yes	yes	-3.5	0.24	56	AG2	no
1429	P	65	0.478	yes	yes	1.6	1.6	56	AG2	no
1430	L	11	0.067	no	-	3.8	0.5	56	AG2	no
1431	V	3	-	no	-	-	-	56	AG2	no
1432	I	109	0.645	yes	yes	4.5	1.96	56	AG2	no
1433	K	68	0.332	yes	yes	-3.9	0.86	56	AG2	no
1434	A	2	-	no	-	-	-	56	AG2	no
1435	L	14	0.085	no	-	3.8	-0.6	56	AG2	no
1436	K	115	0.561	yes	yes	-3.9	-1.64	56	AG2	no

#	Residue	ACC	RSA	Surface	Interface	KD	KD mean	Helix number	Helix label	Calcium ion binding site
1437	Q	25	0.126	no	-	-3.5	-0.95	56	AG2	no
1438	Y	0	-	no	-	-	-	56	AG2	no
1439	T	28	0.197	no	-	-0.7	-2.2	56	AG2	no
1440	T	33	0.232	yes	no	-0.7	-1.4	56	AG2	no
1441	T	17	0.12	no	-	-0.7	-0.62	56	AG2	no
1442	T	20	0.141	no	-	-0.7	1.23	-	-	no
1443	C	63	0.467	yes	no	2.5	0.36	57	AG3	no
1444	V	11	0.077	no	-	4.2	-0.17	57	AG3	no
1445	Q	85	0.429	yes	no	-3.5	-0.17	57	AG3	no
1446	L	1	-	no	-	-	-	57	AG3	no
1447	Q	3	-	no	-	-	-	57	AG3	no
1448	K	71	0.346	yes	no	-3.9	-1.68	57	AG3	no
1449	Q	36	0.182	no	-	-3.5	-3.63	57	AG3	no
1450	V	0	-	no	-	-	-	57	AG3	no
1451	L	1	-	no	-	-	-	57	AG3	no
1452	D	37	0.227	yes	no	-3.5	-3.5	57	AG3	no
1453	L	2	-	no	-	-	-	57	AG3	no
1454	L	1	-	no	-	-	-	57	AG3	no
1455	A	4	-	no	-	-	-	57	AG3	no
1456	Q	23	0.116	no	-	-3.5	-0.93	57	AG3	no
1457	L	2	-	no	-	-	-	57	AG3	no
1458	V	12	0.085	no	-	4.2	0.12	57	AG3	no
1459	Q	88	0.444	yes	yes	-3.5	-0.7	57	AG3	no
1460	L	35	0.213	yes	yes	3.8	0.84	57	AG3	no
1461	R	220	0.887	yes	yes	-4.5	0.84	-	-	no
1462	V	17	0.12	no	-	4.2	0.84	-	-	no
1463	N	100	0.637	yes	yes	-3.5	1.14	58	AG4	no
1464	Y	12	0.054	no	-	-1.3	0.3	58	AG4	no
1465	C	70	0.519	yes	yes	2.5	0.14	58	AG4	no
1466	L	88	0.537	yes	yes	3.8	1.06	58	AG4	no
1467	L	37	0.226	yes	no	3.8	1.06	58	AG4	no
1468	D	16	0.098	no	-	-3.5	-0.9	58	AG4	no
1469	S	99	0.762	yes	no	-0.8	-1.76	-	-	no

#	Residue	ACC	RSA	Surface	Interface	KD	KD mean	Helix number	Helix label	Calcium ion binding site
1470	D	124	0.761	yes	no	-3.5	-1.42	-	-	no
1471	Q	59	0.298	yes	no	-3.5	-0.43	59	AG5	no
1472	V	82	0.577	yes	no	4.2	0.26	59	AG5	no
1473	F	1	-	no	-	-	-	59	AG5	no
1474	I	20	0.118	no	-	4.5	1.9	59	AG5	no
1475	G	36	0.429	yes	no	-0.4	2.98	59	AG5	no
1476	F	39	0.198	no	-	2.8	1.36	59	AG5	no
1477	V	0	-	no	-	-	-	59	AG5	no
1478	L	48	0.293	yes	no	3.8	1.36	59	AG5	no
1479	K	93	0.454	yes	no	-3.9	-0.2	59	AG5	no
1480	Q	6	-	no	-	-	-	59	AG5	no
1481	F	3	-	no	-	-	-	59	AG5	no
1482	E	99	0.51	yes	no	-3.5	-3.05	59	AG5	no
1483	Y	41	0.185	no	-	-1.3	-0.74	59	AG5	no
1484	I	8	-	no	-	-	-	59	AG5	no
1485	E	60	0.309	yes	no	-3.5	-0.82	59	AG5	no
1486	V	71	0.5	yes	no	4.2	-0.9	59	AG5	no
1487	G	13	0.155	no	-	-0.4	-0.28	59	AG5	no
1488	Q	41	0.207	yes	no	-3.5	-0.89	-	-	no
1489	F	13	0.066	no	-	2.8	-1.73	-	-	no
1490	R	95	0.383	yes	no	-4.5	-2.17	-	-	no
1491	E	119	0.613	yes	no	-3.5	-2.17	-	-	no
1492	S	2	-	no	-	-	-	-	-	no
1493	E	89	0.459	yes	no	-3.5	-1.82	-	-	no
1494	A	22	0.208	yes	no	1.8	-0.85	-	-	no
1495	I	0	-	no	-	-	-	60	AG6	no
1496	I	0	-	no	-	-	-	60	AG6	no
1497	P	36	0.265	yes	no	1.6	-0.1	60	AG6	no
1498	N	17	0.108	no	-	-3.5	0.3	60	AG6	no
1499	I	0	-	no	-	-	-	60	AG6	no
1500	F	0	-	no	-	-	-	60	AG6	no
1501	F	37	0.188	no	-	2.8	0.3	60	AG6	no
1502	F	0	-	no	-	-	-	60	AG6	no

#	Residue	ACC	RSA	Surface	Interface	KD	KD mean	Helix number	Helix label	Calcium ion binding site
1503	L	0	-	no	-	-	-	60	AG6	no
1504	V	3	-	no	-	-	-	60	AG6	no
1505	L	22	0.134	no	-	3.8	1.25	60	AG6	no
1506	L	0	-	no	-	-	-	60	AG6	no
1507	S	0	-	no	-	-	-	60	AG6	no
1508	Y	90	0.405	yes	no	-1.3	-0.33	60	AG6	no
1509	E	35	0.18	no	-	-3.5	-3.1	60	AG6	no
1510	R	125	0.504	yes	no	-4.5	-2.96	-	-	no
1511	Y	107	0.482	yes	no	-1.3	-1.53	-	-	no
1512	H	160	0.87	yes	no	-3.2	-2.74	-	-	no
1513	S	100	0.769	yes	no	-0.8	-2.74	-	-	no
1514	K	137	0.668	yes	no	-3.9	-2.87	-	-	no
1515	Q	75	0.379	yes	no	-3.5	-1.85	-	-	no
1516	I	26	0.154	no	-	4.5	1.05	-	-	no
1517	I	31	0.183	no	-	4.5	2.87	-	-	no
1518	G	6	0.071	no	-	-0.4	1.26	61	AG7	no
1519	I	9	0.053	no	-	4.5	0.45	61	AG7	no
1520	P	54	0.397	yes	no	1.6	0.45	61	AG7	no
1521	K	85	0.415	yes	no	-3.9	-0.34	61	AG7	no
1522	I	0	-	no	-	-	-	61	AG7	no
1523	I	8	-	no	-	-	-	61	AG7	no
1524	Q	115	0.581	yes	no	-3.5	-1.5	61	AG7	no
1525	L	11	0.067	no	-	3.8	-0.9	61	AG7	no
1526	C	0	-	no	-	-	-	61	AG7	no
1527	D	48	0.294	yes	no	-3.5	0.02	61	AG7	no
1528	G	22	0.262	yes	no	-0.4	-0.1	61	AG7	no
1529	I	7	-	no	-	-	-	61	AG7	no
1530	M	25	0.133	no	-	1.9	-0.2	61	AG7	no
1531	A	77	0.726	yes	no	1.8	-0.2	61	AG7	no
1532	S	74	0.569	yes	no	-0.8	0.42	61	AG7	no
1533	G	67	0.798	yes	no	-0.4	-2.3	-	-	no
1534	R	140	0.565	yes	no	-4.5	-2.93	-	-	no
1535	K	143	0.698	yes	no	-3.9	-0.62	-	-	no

#	Residue	ACC	RSA	Surface	Interface	KD	KD mean	Helix number	Helix label	Calcium ion binding site
1536	A	10	0.094	no	-	1.8	-0.36	-	-	no
1537	V	57	0.401	yes	no	4.2	-0.36	-	-	no
1538	T	25	0.176	no	-	-0.7	-0.88	-	-	no
1539	H	11	0.06	no	-	-3.2	0.03	62	AG8	no
1540	A	2	-	no	-	-	-	62	AG8	no
1541	I	1	-	no	-	-	-	62	AG8	no
1542	P	18	0.132	no	-	1.6	-0.88	62	AG8	no
1543	A	0	-	no	-	-	-	62	AG8	no
1544	L	0	-	no	-	-	-	62	AG8	no
1545	Q	82	0.414	yes	yes	-3.5	-0.1	62	AG8	no
1546	P	15	0.11	no	-	1.6	-1.16	62	AG8	no
1547	I	3	-	no	-	-	-	62	AG8	no
1548	V	2	-	no	-	-	-	62	AG8	no
1549	H	77	0.418	yes	no	-3.2	0.38	62	AG8	no
1550	D	10	0.061	no	-	-3.5	0.58	62	AG8	no
1551	L	2	-	no	-	-	-	62	AG8	no
1552	F	12	0.061	no	-	2.8	0.62	62	AG8	no
1553	V	51	0.359	yes	yes	4.2	-0.07	62	AG8	no
1554	L	100	0.61	yes	no	3.8	0.56	-	-	no
1555	R	81	0.327	yes	yes	-4.5	1.57	-	-	no
1563	G	107	1	yes	no	-0.4	-0.17	-	-	no
1564	K	181	0.883	yes	no	-3.9	-1.5	63	AG9	no
1565	E	120	0.619	yes	no	-3.5	-1.56	63	AG9	no
1566	L	36	0.22	yes	no	3.8	-1.67	63	AG9	no
1567	E	67	0.345	yes	no	-3.5	-1.88	63	AG9	no
1568	T	62	0.437	yes	no	-0.7	-1.82	63	AG9	no
1569	Q	21	0.106	no	-	-3.5	-0.53	63	AG9	no
1570	K	9	-	no	-	-	-	63	AG9	no
1571	E	108	0.557	yes	no	-3.5	0.14	63	AG9	no
1572	V	56	0.394	yes	no	4.2	0.12	63	AG9	no
1573	V	0	-	no	-	-	-	63	AG9	no
1574	V	12	0.085	no	-	4.2	1.03	63	AG9	no
1575	S	55	0.423	yes	yes	-0.8	2.85	63	AG9	no

#	Residue	ACC	RSA	Surface	Interface	KD	KD mean	Helix number	Helix label	Calcium ion binding site
1576	M	3	-	no	-	-	-	63	AG9	no
1577	L	0	-	no	-	-	-	63	AG9	no
1578	L	27	0.165	no	-	3.8	0.57	63	AG9	no
1579	R	130	0.524	yes	no	-4.5	1.03	63	AG9	no
1580	L	13	0.079	no	-	3.8	-0.1	63	AG9	no
1581	I	1	-	no	-	-	-	-	-	no
1582	Q	47	0.237	yes	no	-3.5	-1.05	-	-	no
1583	Y	44	0.198	no	-	-1.3	-2.94	64	AH1	no
1584	H	38	0.207	yes	no	-3.2	-2.67	64	AH1	no
1585	Q	40	0.202	yes	no	-3.5	-2.88	64	AH1	no
1586	V	0	-	no	-	-	-	64	AH1	no
1587	L	1	-	no	-	-	-	64	AH1	no
1588	E	66	0.34	yes	no	-3.5	-1.7	64	AH1	no
1589	M	10	0.053	no	-	1.9	0.73	64	AH1	no
1590	F	0	-	no	-	-	-	64	AH1	no
1591	I	1	-	no	-	-	-	64	AH1	no
1592	L	51	0.311	yes	yes	3.8	0.73	64	AH1	no
1593	V	0	-	no	-	-	-	64	AH1	no
1594	L	0	-	no	-	-	-	64	AH1	no
1595	Q	84	0.424	yes	no	-3.5	-0.78	64	AH1	no
1596	Q	47	0.237	yes	no	-3.5	-2.32	64	AH1	no
1597	C	11	0.081	no	-	2.5	-2.44	64	AH1	no
1598	H	65	0.353	yes	no	-3.2	-2.56	64	AH1	no
1599	K	158	0.771	yes	no	-3.9	-2.52	64	AH1	no
1600	E	127	0.655	yes	no	-3.5	-2.32	64	AH1	no
1601	N	82	0.522	yes	no	-3.5	-2.81	64	AH1	no
1602	E	105	0.541	yes	no	-3.5	-3.08	-	-	no
1603	D	84	0.515	yes	no	-3.5	-3.2	65	AH2	no
1604	K	54	0.263	yes	no	-3.9	-3.39	65	AH2	no
1605	W	30	0.132	no	-	-0.9	-2.61	65	AH2	no
1606	K	78	0.38	yes	no	-3.9	-3.34	65	AH2	no
1607	R	153	0.617	yes	no	-4.5	-3.54	65	AH2	no
1608	L	7	-	no	-	-	-	65	AH2	no

#	Residue	ACC	RSA	Surface	Interface	KD	KD mean	Helix number	Helix label	Calcium ion binding site
1609	S	0	-	no	-	-	-	65	AH2	no
1610	R	92	0.371	yes	no	-4.5	-4.17	65	AH2	no
1611	Q	93	0.47	yes	no	-3.5	-3.83	65	AH2	no
1612	I	0	-	no	-	-	-	65	AH2	no
1613	A	0	-	no	-	-	-	65	AH2	no
1614	D	87	0.534	yes	yes	-3.5	-0.83	65	AH2	no
1615	I	55	0.325	yes	no	4.5	0.5	65	AH2	no
1616	I	1	-	no	-	-	-	65	AH2	no
1617	L	3	-	no	-	-	-	65	AH2	no
1618	P	58	0.426	yes	no	1.6	1.77	65	AH2	no
1619	M	27	0.144	no	-	1.9	-0.93	65	AH2	no
1620	L	6	-	no	-	-	-	65	AH2	no
1621	A	21	0.198	no	-	1.8	-0.42	65	AH2	no
1622	K	142	0.693	yes	no	-3.9	-1.44	65	AH2	no
1623	Q	29	0.146	no	-	-3.5	-0.57	65	AH2	no
1624	Q	94	0.475	yes	no	-3.5	-2.25	-	-	no
1625	M	2	-	no	-	-	-	-	-	no
1626	H	88	0.478	yes	no	-3.2	-2.88	-	-	no
1627	I	3	-	no	-	-	-	-	-	no
1628	D	34	0.209	yes	no	-3.5	-0.93	-	-	no
1629	S	26	0.2	yes	no	-0.8	-2.75	66	AH3	no
1630	H	13	0.071	no	-	-3.2	-2.5	66	AH3	no
1631	E	112	0.577	yes	no	-3.5	-2.5	66	AH3	no
1632	A	1	-	no	-	-	-	66	AH3	no
1633	L	1	-	no	-	-	-	66	AH3	no
1634	G	0	-	no	-	-	-	66	AH3	no
1635	V	2	-	no	-	-	-	66	AH3	no
1636	L	1	-	no	-	-	-	66	AH3	no
1637	N	2	-	no	-	-	-	66	AH3	no
1638	T	12	0.085	no	-	-0.7	-2.1	66	AH3	no
1639	L	1	-	no	-	-	-	66	AH3	no
1640	F	3	-	no	-	-	-	66	AH3	no
1641	E	45	0.232	yes	no	-3.5	0.1	66	AH3	no

#	Residue	ACC	RSA	Surface	Interface	KD	KD mean	Helix number	Helix label	Calcium ion binding site
1642	I	45	0.266	yes	no	4.5	0.5	66	AH3	no
1643	L	4	-	no	-	-	-	66	AH3	no
1644	A	1	-	no	-	-	-	67	AH4	no
1645	P	59	0.434	yes	no	1.6	0.02	67	AH4	no
1646	S	64	0.492	yes	no	-0.8	0.02	67	AH4	no
1647	S	5	-	no	-	-	-	67	AH4	no
1648	L	11	0.067	no	-	3.8	0.34	67	AH4	no
1649	R	142	0.573	yes	no	-4.5	0.34	67	AH4	no
1650	P	77	0.566	yes	no	1.6	-0.65	-	-	no
1651	V	5	-	no	-	-	-	68	AH5	no
1652	D	30	0.184	no	-	-3.5	0	68	AH5	no
1653	M	71	0.378	yes	no	1.9	-2.03	68	AH5	no
1654	L	3	-	no	-	-	-	68	AH5	no
1655	L	0	-	no	-	-	-	68	AH5	no
1656	R	126	0.508	yes	no	-4.5	-1.3	68	AH5	no
1657	S	5	-	no	-	-	-	68	AH5	no
1658	M	0	-	no	-	-	-	68	AH5	no
1659	F	9	-	no	-	-	-	68	AH5	no
1660	V	26	0.183	no	-	4.2	1.75	-	-	no
1661	T	48	0.338	yes	no	-0.7	1.75	-	-	no
1662	P	1	-	no	-	-	-	-	-	no
1663	N	115	0.732	yes	no	-3.5	-2.1	-	-	no
1664	T	39	0.275	yes	no	-0.7	-0.8	-	-	yes
1665	M	4	-	no	-	-	-	-	-	no
1666	A	60	0.566	yes	no	1.8	-1.54	-	-	no
1667	S	48	0.369	yes	no	-0.8	-0.12	69	AH6	no
1668	V	7	-	no	-	-	-	69	AH6	no
1669	S	49	0.377	yes	no	-0.8	-0.77	69	AH6	no
1670	T	30	0.211	yes	no	-0.7	0.37	69	AH6	no
1671	V	1	-	no	-	-	-	69	AH6	no
1672	Q	0	-	no	-	-	-	69	AH6	no
1673	L	30	0.183	no	-	3.8	-0.98	69	AH6	no
1674	W	1	-	no	-	-	-	69	AH6	no

#	Residue	ACC	RSA	Surface	Interface	KD	KD mean	Helix number	Helix label	Calcium ion binding site
1675	I	2	-	no	-	-	-	69	AH6	no
1676	S	0	-	no	-	-	-	69	AH6	no
1677	G	3	-	no	-	-	-	69	AH6	no
1678	I	4	-	no	-	-	-	69	AH6	no
1679	L	6	-	no	-	-	-	69	AH6	no
1680	A	0	-	no	-	-	-	69	AH6	no
1681	I	1	-	no	-	-	-	69	AH6	no
1682	L	1	-	no	-	-	-	69	AH6	no
1683	R	13	0.052	no	-	-4.5	-0.27	69	AH6	no
1684	V	5	-	no	-	-	-	69	AH6	no
1685	L	3	-	no	-	-	-	69	AH6	no
1686	I	9	0.053	no	-	4.5	-0.4	69	AH6	no
1687	S	16	0.123	no	-	-0.8	-1.02	69	AH6	no
1688	Q	37	0.187	no	-	-3.5	-1.7	69	AH6	no
1689	S	19	0.146	no	-	-0.8	-0.26	69	AH6	no
1690	T	70	0.493	yes	no	-0.7	-0.8	70	AH7	no
1691	E	25	0.129	no	-	-3.5	-0.08	70	AH7	no
1692	D	96	0.589	yes	no	-3.5	-0.8	70	AH7	no
1693	I	69	0.408	yes	yes	4.5	-0.8	70	AH7	no
1694	V	5	-	no	-	-	-	70	AH7	no
1695	L	0	-	no	-	-	-	70	AH7	no
1696	S	44	0.338	yes	no	-0.8	-1.56	70	AH7	no
1697	R	57	0.23	yes	no	-4.5	-3.08	70	AH7	no
1698	I	3	-	no	-	-	-	70	AH7	no
1699	Q	110	0.556	yes	no	-3.5	-1.55	70	AH7	no
1700	E	124	0.639	yes	no	-3.5	-1.7	70	AH7	no
1701	L	33	0.201	yes	yes	3.8	-1	70	AH7	no
1702	S	101	0.777	yes	no	-0.8	-0.24	-	-	no
1703	F	36	0.183	no	-	2.8	0.4	-	-	no
1704	S	58	0.446	yes	no	-0.8	1.12	71	AH8	no
1705	P	4	-	no	-	-	-	71	AH8	no
1706	Y	121	0.545	yes	no	-1.3	0.57	71	AH8	no
1707	L	41	0.25	yes	no	3.8	0.57	71	AH8	no

#	Residue	ACC	RSA	Surface	Interface	KD	KD mean	Helix number	Helix label	Calcium ion binding site
1708	I	1	-	no	-	-	-	71	AH8	no
1709	S	4	-	no	-	-	-	72	AH9	no
1710	C	16	0.119	no	-	2.5	1.3	72	AH9	no
1711	T	87	0.613	yes	no	-0.7	0.62	72	AH9	no
1712	V	24	0.169	no	-	4.2	-0.4	72	AH9	no
1713	I	1	-	no	-	-	-	72	AH9	no
1714	N	90	0.573	yes	no	-3.5	-1.8	72	AH9	no
1715	R	80	0.323	yes	no	-4.5	-2.24	72	AH9	no
1716	L	2	-	no	-	-	-	72	AH9	yes
1717	R	46	0.185	no	-	-4.5	-2.84	72	AH9	no
1718	D	128	0.785	yes	no	-3.5	-2.22	72	AH9	no
1719	G	49	0.583	yes	no	-0.4	-2.98	-	-	yes
1720	D	98	0.601	yes	no	-3.5	-2.3	-	-	no
1721	S	137	1	yes	no	-0.8	-2.15	-	-	no
1735	K	263	1	yes	no	-3.9	-3.7	-	-	no
1736	N	76	0.484	yes	no	-3.5	-1.2	-	-	no
1737	L	76	0.463	yes	yes	3.8	-1.68	73	AI1	no
1738	P	3	-	no	-	-	-	73	AI1	no
1739	E	10	0.052	no	-	-3.5	-1.07	73	AI1	no
1740	E	42	0.216	yes	no	-3.5	-1.07	73	AI1	no
1741	T	2	-	no	-	-	-	73	AI1	no
1742	F	3	-	no	-	-	-	73	AI1	no
1743	S	5	-	no	-	-	-	73	AI1	no
1744	R	7	-	no	-	-	-	73	AI1	no
1745	F	4	-	no	-	-	-	73	AI1	no
1746	L	2	-	no	-	-	-	73	AI1	no
1747	L	8	-	no	-	-	-	73	AI1	no
1748	Q	0	-	no	-	-	-	73	AI1	no
1749	L	2	-	no	-	-	-	73	AI1	no
1750	V	0	-	no	-	-	-	73	AI1	no
1751	G	3	-	no	-	-	-	73	AI1	no
1752	I	0	-	no	-	-	-	73	AI1	no
1753	L	0	-	no	-	-	-	73	AI1	no

#	Residue	ACC	RSA	Surface	Interface	KD	KD mean	Helix number	Helix label	Calcium ion binding site
1754	L	4	-	no	-	-	-	73	AI1	no
1755	E	42	0.216	yes	no	-3.5	0	73	AI1	no
1756	D	4	-	no	-	-	-	73	AI1	no
1757	I	0	-	no	-	-	-	73	AI1	no
1758	V	22	0.155	no	-	4.2	-0.97	73	AI1	no
1759	T	67	0.472	yes	no	-0.7	-0.97	73	AI1	no
1760	K	28	0.137	no	-	-3.9	-0.97	-	-	no
1761	Q	34	0.172	no	-	-3.5	-0.66	-	-	no
1762	L	41	0.25	yes	no	3.8	-1.2	-	-	no
1763	K	128	0.624	yes	no	-3.9	-0.62	-	-	no
1764	V	22	0.155	no	-	4.2	-1.67	-	-	no
1765	E	76	0.392	yes	no	-3.5	-1.07	-	-	no
1766	M	3	-	no	-	-	-	-	-	no
1767	S	36	0.277	yes	no	-0.8	-3.04	74	AI2	no
1768	E	145	0.747	yes	no	-3.5	-2.9	74	AI2	no
1769	Q	100	0.505	yes	no	-3.5	-2.53	74	AI2	no
1770	Q	24	0.121	no	-	-3.5	-2.53	74	AI2	no
1771	H	19	0.103	no	-	-3.2	-2.88	74	AI2	no
1772	T	43	0.303	yes	no	-0.7	-2.73	74	AI2	no
1773	F	1	-	no	-	-	-	74	AI2	no
1774	Y	0	-	no	-	-	-	74	AI2	no
1775	C	0	-	no	-	-	-	74	AI2	no
1776	Q	12	0.061	no	-	-3.5	-2.4	74	AI2	no
1777	E	1	-	no	-	-	-	74	AI2	no
1778	L	0	-	no	-	-	-	74	AI2	no
1779	G	1	-	no	-	-	-	74	AI2	no
1780	T	15	0.106	no	-	-0.7	-0.7	74	AI2	no
1781	L	1	-	no	-	-	-	74	AI2	no
1782	L	2	-	no	-	-	-	74	AI2	no
1783	M	7	-	no	-	-	-	74	AI2	no
1784	C	5	-	no	-	-	-	74	AI2	no
1785	L	4	-	no	-	-	-	74	AI2	no
1786	I	0	-	no	-	-	-	74	AI2	no

#	Residue	ACC	RSA	Surface	Interface	KD	KD mean	Helix number	Helix label	Calcium ion binding site
1787	H	13	0.071	no	-	-3.2	-1.27	74	AI2	no
1788	I	0	-	no	-	-	-	74	AI2	no
1789	F	19	0.096	no	-	2.8	-1.25	74	AI2	no
1790	K	69	0.337	yes	no	-3.9	-1.1	74	AI2	no
1791	S	41	0.315	yes	no	-0.8	-1.28	74	AI2	no
1792	G	16	0.19	no	-	-0.4	-0.82	-	-	no
1793	M	108	0.574	yes	no	1.9	-0.2	-	-	no
1794	F	10	0.051	no	-	2.8	-1.27	75	AI3	no
1795	R	177	0.714	yes	no	-4.5	-0.9	75	AI3	no
1796	R	93	0.375	yes	no	-4.5	-1.02	75	AI3	no
1797	I	4	-	no	-	-	-	75	AI3	no
1798	T	22	0.155	no	-	-0.7	-0.38	75	AI3	no
1799	A	42	0.396	yes	no	1.8	-1.02	75	AI3	no
1800	A	0	-	no	-	-	-	75	AI3	no
1801	A	2	-	no	-	-	-	75	AI3	no
1802	T	22	0.155	no	-	-0.7	0.64	75	AI3	no
1803	R	106	0.427	yes	no	-4.5	-0.62	75	AI3	no
1804	L	13	0.079	no	-	3.8	-0.65	75	AI3	no
1805	F	10	0.051	no	-	2.8	-0.01	75	AI3	no
1806	R	176	0.71	yes	no	-4.5	-0.64	75	AI3	no
1807	S	46	0.354	yes	no	-0.8	-0.44	-	-	no
1808	D	104	0.638	yes	no	-3.5	-1.35	-	-	no
1809	G	21	0.25	yes	no	-0.4	-0.53	-	-	no
1810	C	68	0.504	yes	no	2.5	0.1	-	-	no
1811	G	86	1	yes	no	-0.4	0.1	-	-	no
1812	G	42	0.5	yes	no	-0.4	0.1	-	-	no
1813	S	14	0.108	no	-	-0.8	0.5	-	-	no
1814	F	6	-	no	-	-	-	-	-	no
1815	Y	24	0.108	no	-	-1.3	-1	-	-	no
1816	T	44	0.31	yes	no	-0.7	-0.91	76	AI4	no
1817	L	18	0.11	no	-	3.8	-0.32	76	AI4	no
1818	D	64	0.393	yes	no	-3.5	-0.15	76	AI4	no
1819	S	42	0.323	yes	no	-0.8	-0.15	76	AI4	no

#	Residue	ACC	RSA	Surface	Interface	KD	KD mean	Helix number	Helix label	Calcium ion binding site
1820	L	4	-	no	-	-	-	76	AI4	no
1821	N	13	0.083	no	-	-3.5	-1.31	76	AI4	no
1822	L	74	0.451	yes	no	3.8	-2.17	76	AI4	no
1823	R	75	0.302	yes	no	-4.5	-1.9	76	AI4	no
1824	A	0	-	no	-	-	-	76	AI4	no
1825	R	86	0.347	yes	no	-4.5	-1.9	76	AI4	no
1826	S	11	0.085	no	-	-0.8	-3.27	76	AI4	no
1827	M	2	-	no	-	-	-	-	-	no
1828	I	4	-	no	-	-	-	-	-	no
1829	T	0	-	no	-	-	-	-	-	no
1830	T	23	0.162	no	-	-0.7	-0.7	-	-	no
1831	H	3	-	no	-	-	-	77	AI5	no
1832	P	0	-	no	-	-	-	77	AI5	no
1833	A	4	-	no	-	-	-	77	AI5	no
1834	L	0	-	no	-	-	-	77	AI5	no
1835	V	0	-	no	-	-	-	77	AI5	no
1836	L	7	-	no	-	-	-	77	AI5	no
1837	L	2	-	no	-	-	-	77	AI5	no
1838	W	3	-	no	-	-	-	77	AI5	no
1839	C	0	-	no	-	-	-	77	AI5	no
1840	Q	8	-	no	-	-	-	77	AI5	no
1841	I	0	-	no	-	-	-	77	AI5	no
1842	L	0	-	no	-	-	-	77	AI5	no
1843	L	4	-	no	-	-	-	77	AI5	no
1844	L	31	0.189	no	-	3.8	0.15	77	AI5	no
1845	V	5	-	no	-	-	-	77	AI5	no
1846	N	82	0.522	yes	no	-3.5	-0.9	-	-	no
1847	H	41	0.223	yes	no	-3.2	-2.73	-	-	no
1848	T	30	0.211	yes	no	-0.7	-2.73	-	-	no
1849	D	84	0.515	yes	no	-3.5	-2.64	-	-	no
1850	Y	73	0.329	yes	no	-1.3	-1.88	-	-	no
1851	R	163	0.657	yes	no	-4.5	-1.88	-	-	no
1852	W	8	-	no	-	-	-	78	AI6	no

#	Residue	ACC	RSA	Surface	Interface	KD	KD mean	Helix number	Helix label	Calcium ion binding site
1853	W	0	-	no	-	-	-	78	AI6	no
1854	A	28	0.264	yes	no	1.8	-1.95	78	AI6	no
1855	E	66	0.34	yes	no	-3.5	-1.73	78	AI6	no
1856	V	3	-	no	-	-	-	78	AI6	no
1857	Q	1	-	no	-	-	-	78	AI6	no
1858	Q	96	0.485	yes	no	-3.5	-1.48	-	-	no
1859	T	27	0.19	no	-	-0.7	-0.2	-	-	no
1860	P	73	0.537	yes	no	1.6	-1	-	-	no
1861	K	251	1	yes	no	-3.9	-1.15	-	-	no
1887	K	173	0.844	yes	no	-3.9	-0.17	-	-	no
1888	L	11	0.067	no	-	3.8	-1	-	-	no
1889	G	11	0.131	no	-	-0.4	0.12	-	-	no
1890	M	11	0.059	no	-	1.9	-1.35	79	AI7	no
1891	C	3	-	no	-	-	-	79	AI7	no
1892	N	17	0.108	no	-	-3.5	-2.4	79	AI7	no
1893	R	63	0.254	yes	no	-4.5	-2.4	79	AI7	no
1894	E	18	0.093	no	-	-3.5	-4	79	AI7	no
1895	I	1	-	no	-	-	-	79	AI7	no
1896	V	1	-	no	-	-	-	79	AI7	no
1897	R	28	0.113	no	-	-4.5	-4.17	79	AI7	no
1898	R	53	0.214	yes	no	-4.5	-4.5	79	AI7	no
1899	G	3	-	no	-	-	-	79	AI7	no
1900	A	0	-	no	-	-	-	79	AI7	no
1901	L	0	-	no	-	-	-	79	AI7	no
1902	I	1	-	no	-	-	-	79	AI7	no
1903	L	5	-	no	-	-	-	79	AI7	no
1904	F	0	-	no	-	-	-	79	AI7	no
1905	C	0	-	no	-	-	-	79	AI7	no
1906	D	24	0.147	no	-	-3.5	-0.77	79	AI7	no
1907	Y	23	0.104	no	-	-1.3	-1.45	79	AI7	no
1908	V	2	-	no	-	-	-	79	AI7	no
1909	C	23	0.17	no	-	2.5	-0.92	79	AI7	no
1910	Q	139	0.702	yes	no	-3.5	-0.4	79	AI7	no

#	Residue	ACC	RSA	Surface	Interface	KD	KD mean	Helix number	Helix label	Calcium ion binding site
1911	N	45	0.287	yes	no	-3.5	-0.78	79	AI7	no
1912	L	57	0.348	yes	no	3.8	-1.23	-	-	no
1913	H	140	0.761	yes	no	-3.2	-1.6	-	-	no
1914	D	80	0.491	yes	no	-3.5	-0.93	-	-	yes
1915	S	52	0.4	yes	no	-0.8	-2.34	80	AI8	no
1916	E	92	0.474	yes	no	-3.5	-1.82	80	AI8	yes
1917	H	24	0.13	no	-	-3.2	-1.82	80	AI8	no
1918	L	2	-	no	-	-	-	80	AI8	no
1919	T	64	0.451	yes	no	-0.7	-1.82	80	AI8	no
1920	W	32	0.141	no	-	-0.9	-1.27	80	AI8	no
1921	L	0	-	no	-	-	-	80	AI8	no
1922	I	1	-	no	-	-	-	80	AI8	no
1923	V	80	0.563	yes	no	4.2	0.4	80	AI8	no
1924	N	54	0.344	yes	no	-3.5	-0.07	80	AI8	no
1925	H	3	-	no	-	-	-	80	AI8	no
1926	I	4	-	no	-	-	-	80	AI8	no
1927	Q	51	0.258	yes	no	-3.5	-3.5	80	AI8	no
1928	D	30	0.184	no	-	-3.5	-2.6	80	AI8	no
1929	L	0	-	no	-	-	-	80	AI8	no
1930	I	0	-	no	-	-	-	80	AI8	no
1931	S	29	0.223	yes	no	-0.8	-1.7	80	AI8	no
1932	L	5	-	no	-	-	-	80	AI8	no
1933	S	17	0.131	no	-	-0.8	-2.08	-	-	no
1934	H	85	0.462	yes	no	-3.2	-2	-	-	no
1935	E	6	-	no	-	-	-	81	AI9	no
1936	P	69	0.507	yes	no	1.6	-1.8	81	AI9	no
1937	P	2	-	no	-	-	-	81	AI9	no
1938	V	0	-	no	-	-	-	81	AI9	no
1939	Q	102	0.515	yes	no	-3.5	-0.34	81	AI9	no
1940	D	85	0.521	yes	no	-3.5	-0.34	81	AI9	no
1941	F	0	-	no	-	-	-	81	AI9	no
1942	I	11	0.065	no	-	4.5	-0.82	81	AI9	no
1943	S	47	0.362	yes	no	-0.8	-0.75	81	AI9	no

#	Residue	ACC	RSA	Surface	Interface	KD	KD mean	Helix number	Helix label	Calcium ion binding site
1944	A	5	-	no	-	-	-	81	AI9	no
1945	V	0	-	no	-	-	-	81	AI9	no
1946	H	31	0.168	no	-	-3.2	-3.04	81	AI9	no
1947	R	169	0.681	yes	no	-4.5	-3.73	81	AI9	no
1948	N	53	0.338	yes	no	-3.5	-1.4	82	AJ1	no
1949	S	37	0.285	yes	no	-0.8	-0.78	82	AJ1	no
1950	A	64	0.604	yes	no	1.8	-0.18	82	AJ1	no
1951	A	7	0.066	no	-	1.8	0.62	82	AJ1	no
1952	S	0	-	no	-	-	-	82	AJ1	no
1953	G	2	-	no	-	-	-	82	AJ1	no
1954	L	51	0.311	yes	no	3.8	0.7	82	AJ1	no
1955	F	0	-	no	-	-	-	82	AJ1	no
1956	I	1	-	no	-	-	-	82	AJ1	no
1957	Q	108	0.545	yes	no	-3.5	-0.35	82	AJ1	no
1958	A	13	0.123	no	-	1.8	-0.36	82	AJ1	no
1959	I	0	-	no	-	-	-	82	AJ1	no
1960	Q	47	0.237	yes	no	-3.5	-1.33	82	AJ1	no
1961	S	95	0.731	yes	no	-0.8	-1.75	82	AJ1	no
1962	R	84	0.339	yes	no	-4.5	-1.96	-	-	no
1963	C	27	0.2	yes	no	2.5	-2.5	-	-	no
1964	E	152	0.784	yes	no	-3.5	-1.96	-	-	no
1965	N	79	0.503	yes	no	-3.5	-1.32	-	-	no
1966	L	1	-	no	-	-	-	-	-	no
1967	S	65	0.5	yes	no	-0.8	-2.12	-	-	no
1968	T	68	0.528	yes	no	-0.7	0.26	83	AJ2	no
1969	P	4	0.397	yes	no	1.6	0.53	83	AJ2	no
1970	T	29	0.592	yes	no	-0.7	-0.36	83	AJ2	no
1971	M	25	0.133	no	-	1.9	-0.95	83	AJ2	no
1972	L	2	-	no	-	-	-	83	AJ2	no
1973	K	18	0.434	yes	yes	-3.9	-1.65	83	AJ2	no
1974	K	42	0.205	yes	no	-3.9	-2.35	83	AJ2	no
1975	T	1	-	no	-	-	-	83	AJ2	no
1976	L	0	-	no	-	-	-	83	AJ2	no

#	Residue	ACC	RSA	Surface	Interface	KD	KD mean	Helix number	Helix label	Calcium ion binding site
1977	Q	72	0.525	yes	no	-3.5	-3.63	83	AJ2	no
1978	C	4	-	no	-	-	-	83	AJ2	no
1979	L	0	-	no	-	-	-	83	AJ2	no
1980	E	54	0.412	yes	yes	-3.5	-2.47	83	AJ2	no
1981	G	39	0.464	yes	no	-0.4	-1.95	83	AJ2	no
1982	I	3	-	no	-	-	-	-	-	no
1983	H	50	0.272	yes	no	-3.2	-1.28	84	AJ3	no
1984	L	106	0.646	yes	no	3.8	-0.93	84	AJ3	no
1985	S	60	0.462	yes	no	-0.8	-0.9	84	AJ3	no
1986	Q	43	0.217	yes	no	-3.5	-0.38	84	AJ3	no
1987	S	2	-	no	-	-	-	84	AJ3	no
1988	G	2	-	no	-	-	-	85	AJ4	no
1989	A	30	0.283	yes	no	1.8	-0.8	85	AJ4	no
1990	V	0	-	no	-	-	-	85	AJ4	no
1991	L	0	-	no	-	-	-	85	AJ4	no
1992	T	21	0.148	no	-	-0.7	-0.8	85	AJ4	no
1993	L	0	-	no	-	-	-	85	AJ4	no
1994	Y	0	-	no	-	-	-	85	AJ4	no
1995	V	0	-	no	-	-	-	85	AJ4	no
1996	D	34	0.209	yes	no	-3.5	-2.9	85	AJ4	no
1997	R	110	0.444	yes	no	-4.5	-1.4	85	AJ4	no
1998	L	9	0.055	no	-	3.8	0.6	-	-	no
1999	L	0	-	no	-	-	-	-	-	no
2000	C	81	0.6	yes	no	2.5	1.87	-	-	no
2001	T	19	0.134	no	-	-0.7	1.55	-	-	no
2002	P	64	0.831	yes	no	1.6	-0.2	-	-	no
2003	F	21	0.457	yes	no	2.8	0.68	86	AJ5	no
2004	R	11	0.415	yes	no	-4.5	1.02	86	AJ5	no
2005	V	0	0.655	yes	yes	4.2	-0.5	86	AJ5	no
2006	L	0	-	no	-	-	-	86	AJ5	no
2007	A	0	-	no	-	-	-	86	AJ5	no
2008	R	91	0.605	yes	yes	-4.5	-0.47	86	AJ5	no
2009	M	0	0.282	yes	no	1.9	-0.4	86	AJ5	no

#	Residue	ACC	RSA	Surface	Interface	KD	KD mean	Helix number	Helix label	Calcium ion binding site
2010	V	0	-	no	-	-	-	86	AJ5	no
2011	D	21	0.129	no	-	-3.5	0.44	86	AJ5	no
2012	I	36	0.45	yes	yes	4.5	1.67	86	AJ5	no
2013	L	1	0.067	no	-	3.8	0.08	86	AJ5	no
2014	A	0	-	no	-	-	-	86	AJ5	no
2015	C	5	-	no	-	-	-	86	AJ5	no
2016	R	94	0.452	yes	yes	-4.5	-2.17	86	AJ5	no
2017	R	13	0.052	no	-	-4.5	-2.65	86	AJ5	no
2018	V	0	-	no	-	-	-	86	AJ5	no
2019	E	76	0.392	yes	yes	-3.5	-1.36	86	AJ5	no
2020	M	54	0.287	yes	yes	1.9	0.22	86	AJ5	no
2021	L	2	-	no	-	-	-	86	AJ5	no
2022	L	48	0.293	yes	yes	3.8	1.16	86	AJ5	no
2023	A	81	0.764	yes	yes	1.8	2.33	86	AJ5	no
2024	A	38	0.358	yes	yes	1.8	1.16	86	AJ5	no
2025	N	132	0.841	yes	yes	-3.5	0.7	87	AJ6	no
2026	L	76	0.463	yes	yes	3.8	-0.42	87	AJ6	no
2027	Q	187	0.944	yes	no	-3.5	0.35	87	AJ6	no
2028	S	48	0.369	yes	no	-0.8	0.64	87	AJ6	no
2029	S	0	-	no	-	-	-	87	AJ6	no
2030	M	81	0.431	yes	no	1.9	0.19	87	AJ6	no
2031	A	74	0.698	yes	no	1.8	-0.15	87	AJ6	no
2032	Q	47	0.237	yes	no	-3.5	0.07	87	AJ6	no
2033	L	4	-	no	-	-	-	87	AJ6	no
2034	P	58	0.426	yes	no	1.6	-0.32	88	AJ7	no
2035	M	151	0.803	yes	yes	1.9	-0.88	88	AJ7	no
2036	E	113	0.582	yes	no	-3.5	-1.4	88	AJ7	no
2037	E	38	0.196	no	-	-3.5	-1.92	88	AJ7	no
2038	L	8	-	no	-	-	-	88	AJ7	no
2039	N	69	0.439	yes	no	-3.5	-3.7	88	AJ7	no
2040	R	83	0.335	yes	no	-4.5	-3.75	88	AJ7	no
2041	I	0	-	no	-	-	-	88	AJ7	no
2042	Q	4	-	no	-	-	-	88	AJ7	no

#	Residue	ACC	RSA	Surface	Interface	KD	KD mean	Helix number	Helix label	Calcium ion binding site
2043	E	123	0.634	yes	no	-3.5	-2.85	88	AJ7	no
2044	Y	61	0.275	yes	no	-1.3	-1.98	88	AJ7	no
2045	L	0	-	no	-	-	-	88	AJ7	no
2046	Q	108	0.545	yes	no	-3.5	-1.35	88	AJ7	no
2047	S	100	0.769	yes	no	-0.8	-1.98	88	AJ7	no
2048	S	43	0.331	yes	no	-0.8	-1.36	88	AJ7	no
2049	G	36	0.429	yes	no	-0.4	-0.73	89	AJ8	no
2050	L	5	-	no	-	-	-	89	AJ8	no
2051	A	7	0.066	no	-	1.8	-2.13	89	AJ8	no
2052	Q	96	0.485	yes	no	-3.5	-2.02	89	AJ8	no
2053	R	101	0.589	yes	yes	-4.5	-2.07	89	AJ8	no
2054	H	7	-	no	-	-	-	90	AJ9	no
2055	Q	133	0.672	yes	yes	-3.5	-2.2	90	AJ9	no
2056	R	127	0.512	yes	yes	-4.5	-2.53	90	AJ9	no
2057	L	0	-	no	-	-	-	90	AJ9	no
2058	Y	43	0.194	no	-	-1.3	-1.66	90	AJ9	no
2059	S	49	0.377	yes	no	-0.8	-2.52	90	AJ9	no
2060	L	1	-	no	-	-	-	90	AJ9	no
2061	L	0	-	no	-	-	-	90	AJ9	no
2062	D	57	0.35	yes	no	-3.5	-3.33	90	AJ9	no
2063	R	130	0.524	yes	yes	-4.5	-2.17	90	AJ9	no
2064	F	4	-	no	-	-	-	90	AJ9	no
2065	R	74	0.298	yes	yes	-4.5	-1.19	90	AJ9	no
2066	L	140	0.854	yes	yes	3.8	-0.8	90	AJ9	no
2067	S	59	0.454	yes	yes	-0.8	-0.06	90	AJ9	no
2068	T	24	0.169	no	-	-0.7	-0.06	90	AJ9	no
2069	M	176	0.936	yes	yes	1.9	-0.06	90	AJ9	no
2095	V	139	0.979	yes	no	4.2	1.7	-	-	no
2096	S	67	0.515	yes	no	-0.8	0.38	-	-	no
2097	P	12	0.088	no	-	1.6	-0.9	-	-	no
2098	D	67	0.411	yes	no	-3.5	-1.7	91	AK1	no
2099	K	64	0.312	yes	no	-3.9	-2.95	91	AK1	no
2100	D	96	0.589	yes	no	-3.5	-1.52	91	AK1	no

#	Residue	ACC	RSA	Surface	Interface	KD	KD mean	Helix number	Helix label	Calcium ion binding site
2101	W	31	0.137	no	-	-0.9	-1.31	91	AK1	no
2102	Y	0	-	no	-	-	-	91	AK1	no
2103	V	17	0.12	no	-	4.2	0.08	91	AK1	no
2104	H	126	0.685	yes	no	-3.2	0	91	AK1	no
2105	L	21	0.128	no	-	3.8	0.02	91	AK1	no
2106	V	0	-	no	-	-	-	91	AK1	no
2107	K	55	0.268	yes	no	-3.9	-1.42	91	AK1	no
2108	S	80	0.615	yes	no	-0.8	-1.1	91	AK1	no
2109	Q	31	0.157	no	-	-3.5	-2.27	91	AK1	no
2110	C	1	-	no	-	-	-	91	AK1	no
2111	W	45	0.198	no	-	-0.9	-2.5	91	AK1	yes
2112	T	57	0.401	yes	no	-0.7	-2.03	-	-	yes
2113	R	206	0.835	yes	no	-4.5	-2	-	-	no
2114	S	40	0.338	yes	no	-0.8	-2.4	-	-	no
2115	D	10	0.601	yes	no	-3.5	-0.32	-	-	yes
2116	S	82	0.769	yes	no	-0.8	-0.82	-	-	no
2117	A	11	0.104	no	-	1.8	1.02	92	AK2	no
2118	L	11	0.262	yes	yes	3.8	0.48	92	AK2	no
2119	L	23	0.628	yes	yes	3.8	1.5	92	AK2	no
2120	E	52	0.278	yes	no	-3.5	0.67	92	AK2	no
2121	G	1	-	no	-	-	-	92	AK2	no
2122	A	1	-	no	-	-	-	92	AK2	no
2123	E	79	0.505	yes	no	-3.5	-3.5	92	AK2	no
2124	L	0	-	no	-	-	-	92	AK2	no
2125	V	0	-	no	-	-	-	92	AK2	no
2126	N	41	0.261	yes	no	-3.5	-3.83	92	AK2	no
2127	R	126	0.508	yes	no	-4.5	-4	-	-	no
2128	I	3	-	no	-	-	-	-	-	no
2129	P	16	0.118	no	-	1.6	-0.9	93	AK3	no
2130	A	61	0.575	yes	no	1.8	-0.36	93	AK3	no
2131	E	161	0.83	yes	no	-3.5	-0.9	93	AK3	no
2132	D	44	0.27	yes	no	-3.5	-0.88	93	AK3	no
2133	M	5	-	no	-	-	-	93	AK3	no

#	Residue	ACC	RSA	Surface	Interface	KD	KD mean	Helix number	Helix label	Calcium ion binding site
2134	N	63	0.401	yes	no	-3.5	-0.12	-	-	no
2135	A	79	0.745	yes	no	1.8	-0.1	-	-	no
2136	F	10	0.051	no	-	2.8	0.75	-	-	no
2137	M	0	-	no	-	-	-	-	-	no
2138	M	67	0.356	yes	no	1.9	0.75	-	-	no
2139	N	61	0.389	yes	no	-3.5	-1.48	-	-	no
2140	S	104	0.8	yes	no	-0.8	-2.6	-	-	no
2141	E	101	0.521	yes	no	-3.5	-2.17	-	-	yes
2142	F	6	-	no	-	-	-	-	-	no
2143	N	7	-	no	-	-	-	94	AK4	no
2144	L	27	0.165	no	-	3.8	1.5	94	AK4	no
2145	S	13	0.1	no	-	-0.8	0.77	94	AK4	no
2146	L	4	-	no	-	-	-	95	AK5	no
2147	L	0	-	no	-	-	-	95	AK5	no
2148	A	15	0.142	no	-	1.8	-0.03	95	AK5	no
2149	P	14	0.125	no	-	1.6	2.04	95	AK5	no
2150	C	0	-	no	-	-	-	95	AK5	no
2151	L	0	-	no	-	-	-	95	AK5	no
2152	S	20	0.331	yes	no	-0.8	1.62	95	AK5	no
2153	L	7	0.159	no	-	3.8	1.14	95	AK5	no
2154	G	0	-	no	-	-	-	95	AK5	no
2155	M	15	0.08	no	-	1.9	0.61	95	AK5	no
2156	S	45	0.515	yes	no	-0.8	0.85	95	AK5	no
2157	E	35	0.18	no	-	-3.5	-0.37	95	AK5	no
2158	I	21	0.124	no	-	4.5	-0.77	95	AK5	no
2159	S	65	0.5	yes	no	-0.8	0.07	95	AK5	no
2160	G	64	0.762	yes	no	-0.4	-0.12	-	-	no
2161	G	66	0.786	yes	no	-0.4	-0.12	-	-	no
2162	Q	144	0.727	yes	no	-3.5	-1.36	-	-	no
2163	K	140	0.683	yes	no	-3.9	-0.97	-	-	no
2164	S	3	-	no	-	-	-	96	AK6	no
2165	A	25	0.236	yes	no	1.8	0.48	96	AK6	no
2166	L	0	-	no	-	-	-	96	AK6	no

#	Residue	ACC	RSA	Surface	Interface	KD	KD mean	Helix number	Helix label	Calcium ion binding site
2167	F	1	-	no	-	-	-	96	AK6	no
2168	E	41	0.211	yes	no	-3.5	-1.1	96	AK6	no
2169	A	6	0.057	no	-	1.8	-1.9	96	AK6	no
2170	A	0	-	no	-	-	-	96	AK6	no
2171	R	23	0.093	no	-	-4.5	-2.42	96	AK6	no
2172	E	66	0.34	yes	no	-3.5	-1.1	96	AK6	no
2173	V	0	-	no	-	-	-	96	AK6	no
2174	T	0	-	no	-	-	-	96	AK6	no
2175	L	1	-	no	-	-	-	96	AK6	no
2176	A	38	0.358	yes	no	1.8	-1.75	96	AK6	no
2177	R	53	0.214	yes	no	-4.5	-0.98	96	AK6	no
2178	V	1	-	no	-	-	-	96	AK6	no
2179	S	16	0.123	no	-	-0.8	-0.56	96	AK6	no
2180	G	25	0.298	yes	no	-0.4	-0.95	96	AK6	no
2181	T	11	0.077	no	-	-0.7	-0.78	96	AK6	no
2182	V	13	0.092	no	-	4.2	-0.13	96	AK6	no
2183	Q	158	0.798	yes	no	-3.5	-0.02	96	AK6	no
2184	Q	115	0.581	yes	no	-3.5	0.06	96	AK6	no
2185	L	40	0.244	yes	no	3.8	0.52	-	-	no
2186	P	33	0.243	yes	no	1.6	2.85	-	-	no
2187	A	108	1	yes	no	1.8	2.53	-	-	no
2188	V	101	0.711	yes	no	4.2	1.1	-	-	no
2189	H	28	0.152	no	-	-3.2	-0.73	-	-	no
2190	H	74	0.402	yes	no	-3.2	-3.2	-	-	no
2191	V	6	-	no	-	-	-	-	-	no
2192	F	2	-	no	-	-	-	-	-	no
2193	Q	47	0.237	yes	no	-3.5	-1.8	-	-	no
2194	P	35	0.257	yes	no	1.6	-0.4	-	-	no
2195	E	142	0.732	yes	no	-3.5	-0.4	-	-	no
2196	L	113	0.689	yes	no	3.8	1.06	-	-	no
2197	P	31	0.228	yes	no	1.6	0.93	-	-	no
2198	A	67	0.632	yes	no	1.8	1.06	-	-	no
2199	E	154	0.794	yes	no	-3.5	0.66	-	-	no

#	Residue	ACC	RSA	Surface	Interface	KD	KD mean	Helix number	Helix label	Calcium ion binding site
2200	P	82	0.603	yes	no	1.6	0.43	-	-	no
2201	A	41	0.387	yes	no	1.8	0.43	-	-	no
2202	A	63	0.594	yes	no	1.8	0.93	97	AK7	no
2203	Y	11	-	no	-	-	-	97	AK7	no
2204	W	10	-	no	-	-	-	97	AK7	no
2205	S	65	0.5	yes	no	-0.8	-1.6	97	AK7	no
2206	K	109	0.532	yes	no	-3.9	-1.58	97	AK7	no
2207	L	2	-	no	-	-	-	97	AK7	no
2208	N	68	0.433	yes	no	-3.5	-1.38	97	AK7	no
2209	D	112	0.687	yes	no	-3.5	-1.5	97	AK7	yes
2210	L	21	0.128	no	-	3.8	-1.5	97	AK7	yes
2211	F	1	-	no	-	-	-	97	AK7	no
2212	G	15	0.179	no	-	-0.4	-1.42	-	-	no
2213	D	78	0.479	yes	no	-3.5	-0.07	-	-	yes
2214	A	57	0.538	yes	no	1.8	0.08	-	-	no
2215	A	97	0.915	yes	no	1.8	0.08	-	-	no
2216	L	16	0.098	no	-	3.8	0.62	98	AK8	yes
2217	Y	4	-	no	-	-	-	98	AK8	no
2218	Q	98	0.495	yes	no	-3.5	0.62	98	AK8	no
2219	S	26	0.2	yes	no	-0.8	-0.3	99	AK9	no
2220	L	7	-	no	-	-	-	99	AK9	no
2221	P	1	-	no	-	-	-	99	AK9	no
2222	T	21	0.148	no	-	-0.7	-1.7	99	AK9	no
2223	L	3	-	no	-	-	-	99	AK9	no
2224	A	0	-	no	-	-	-	99	AK9	no
2225	R	37	0.202	yes	no	-4.5	-2.6	99	AK9	no
2226	A	0	-	no	-	-	-	99	AK9	no
2227	L	0	-	no	-	-	-	99	AK9	no
2228	A	0	-	no	-	-	-	99	AK9	no
2229	Q	30	0.152	no	-	-3.5	0.83	99	AK9	no
2230	Y	0	-	no	-	-	-	99	AK9	no
2231	L	2	-	no	-	-	-	99	AK9	no
2232	V	10	0.07	no	-	4.2	2.28	99	AK9	no

#	Residue	ACC	RSA	Surface	Interface	KD	KD mean	Helix number	Helix label	Calcium ion binding site
2233	V	12	0.085	no	-	4.2	1.58	100	AL1	no
2234	V	14	0.099	no	-	4.2	1.21	100	AL1	no
2235	S	99	0.762	yes	no	-0.8	0.93	100	AL1	no
2236	K	93	0.454	yes	no	-3.9	1.5	100	AL1	no
2237	L	15	0.091	no	-	3.8	1.43	100	AL1	no
2238	P	56	0.412	yes	no	1.6	0.35	101	AL2	no
2239	S	99	0.762	yes	no	-0.8	-1.4	101	AL2	no
2240	H	125	0.679	yes	no	-3.2	-1.4	101	AL2	no
2241	L	4	-	no	-	-	-	101	AL2	no
2242	H	76	0.413	yes	no	-3.2	0.16	101	AL2	no
2243	L	17	0.104	no	-	3.8	0.73	-	-	no
2244	P	42	0.309	yes	no	1.6	-0.08	-	-	no
2245	P	107	0.787	yes	no	1.6	-1.54	-	-	no
2246	E	146	0.753	yes	no	-3.5	-1.54	102	AL3	yes
2247	K	49	0.239	yes	no	-3.9	-2.16	102	AL3	no
2248	E	38	0.196	no	-	-3.5	-2.78	102	AL3	no
2249	K	83	0.405	yes	no	-3.9	-3.7	102	AL3	no
2250	D	31	0.19	no	-	-3.5	-3.25	102	AL3	yes
2251	I	1	-	no	-	-	-	102	AL3	no
2252	V	5	-	no	-	-	-	102	AL3	no
2253	K	71	0.346	yes	no	-3.9	-3.7	102	AL3	no
2254	F	1	-	no	-	-	-	102	AL3	no
2255	V	5	-	no	-	-	-	102	AL3	no
2256	V	1	-	no	-	-	-	102	AL3	no
2257	A	5	-	no	-	-	-	102	AL3	no
2258	T	1	-	no	-	-	-	102	AL3	no
2259	L	0	-	no	-	-	-	102	AL3	no
2260	E	4	-	no	-	-	-	102	AL3	no
2261	A	1	-	no	-	-	-	102	AL3	no
2262	L	1	-	no	-	-	-	102	AL3	no
2263	S	0	-	no	-	-	-	102	AL3	no
2264	W	21	0.093	no	-	-0.9	0.13	102	AL3	no
2265	H	25	0.136	no	-	-3.2	-0.78	102	AL3	no

#	Residue	ACC	RSA	Surface	Interface	KD	KD mean	Helix number	Helix label	Calcium ion binding site
2266	L	0	-	no	-	-	-	102	AL3	no
2267	I	26	0.154	no	-	4.5	-0.7	102	AL3	no
2268	H	97	0.527	yes	yes	-3.2	-1.35	102	AL3	no
2269	E	75	0.387	yes	no	-3.5	-3.35	102	AL3	no
2270	Q	94	0.737	yes	yes	-3.5	-0.83	-	-	no
2271	I	3	0.408	yes	yes	4.5	0.48	-	-	no
2272	P	6	0.11	no	-	1.6	1.68	-	-	no
2273	L	12	0.457	yes	yes	3.8	2.1	103	AL4	no
2274	S	0	0.485	yes	yes	-0.8	2.27	103	AL4	no
2275	L	4	0.482	yes	yes	3.8	0.82	103	AL4	no
2276	D	6	-	no	-	-	-	103	AL4	no
2277	L	0	-	no	-	-	-	103	AL4	no
2278	Q	4	0.126	no	-	-3.5	0.15	103	AL4	no
2279	A	1	-	no	-	-	-	103	AL4	no
2280	G	0	-	no	-	-	-	103	AL4	no
2281	L	0	-	no	-	-	-	103	AL4	no
2282	D	2	-	no	-	-	-	103	AL4	no
2283	C	0	-	no	-	-	-	103	AL4	no
2284	C	0	-	no	-	-	-	103	AL4	no
2285	C	0	-	no	-	-	-	103	AL4	no
2286	L	4	-	no	-	-	-	103	AL4	no
2287	A	5	-	no	-	-	-	103	AL4	no
2288	L	4	-	no	-	-	-	103	AL4	no
2289	Q	34	0.172	no	-	-3.5	0.15	-	-	no
2290	L	27	0.165	no	-	3.8	0.12	104	AL5	no
2291	P	93	0.684	yes	no	1.6	0.66	104	AL5	no
2292	G	18	0.214	yes	no	-0.4	0.66	104	AL5	no
2293	L	1	-	no	-	-	-	104	AL5	no
2294	W	47	0.207	yes	no	-0.9	0.96	104	AL5	no
2295	S	75	0.577	yes	no	-0.8	0.48	104	AL5	no
2296	V	16	0.113	no	-	4.2	0.18	104	AL5	no
2297	V	4	-	no	-	-	-	104	AL5	no
2298	S	22	0.169	no	-	-0.8	0.18	104	AL5	no

#	Residue	ACC	RSA	Surface	Interface	KD	KD mean	Helix number	Helix label	Calcium ion binding site
2299	S	33	0.254	yes	no	-0.8	-0.32	-	-	no
2300	T	56	0.394	yes	no	-0.7	-0.2	-	-	no
2301	E	120	0.619	yes	no	-3.5	-0.2	-	-	no
2302	F	5	-	no	-	-	-	105	AL6	no
2303	V	27	0.19	no	-	4.2	-0.78	105	AL6	no
2304	T	26	0.183	no	-	-0.7	0.1	105	AL6	no
2305	H	16	0.087	no	-	-3.2	0.1	105	AL6	no
2306	A	4	-	no	-	-	-	105	AL6	no
2307	C	0	-	no	-	-	-	105	AL6	no
2308	S	0	-	no	-	-	-	105	AL6	no
2309	L	4	-	no	-	-	-	105	AL6	no
2310	I	0	-	no	-	-	-	105	AL6	no
2311	H	10	0.054	no	-	-3.2	-3.2	105	AL6	no
2312	C	0	-	no	-	-	-	105	AL6	no
2313	V	0	-	no	-	-	-	105	AL6	no
2314	H	16	0.087	no	-	-3.2	-3.2	105	AL6	no
2315	F	6	-	no	-	-	-	105	AL6	no
2316	I	1	-	no	-	-	-	105	AL6	no
2317	L	1	-	no	-	-	-	105	AL6	no
2318	E	35	0.18	no	-	-3.5	0.83	105	AL6	no
2319	A	0	-	no	-	-	-	105	AL6	no
2320	V	7	0.113	no	-	4.2	0.45	105	AL6	no
2321	A	22	0.208	yes	no	1.8	0.52	105	AL6	no
2322	V	7	-	no	-	-	-	105	AL6	no
2323	Q	39	0.197	no	-	-3.5	-3.5	-	-	no
2324	P	6	-	no	-	-	-	-	-	no
2325	G	32	0.381	yes	no	-0.4	-2.47	-	-	no
2326	E	43	0.222	yes	no	-3.5	-2.73	-	-	no
2327	Q	146	0.737	yes	no	-3.5	-1.07	-	-	no
2328	L	3	-	no	-	-	-	-	-	no
2329	L	36	0.22	yes	no	3.8	-0.17	-	-	no
2330	S	44	0.338	yes	no	-0.8	1.53	-	-	no
2331	P	148	1	yes	no	1.6	0.4	-	-	no

#	Residue	ACC	RSA	Surface	Interface	KD	KD mean	Helix number	Helix label	Calcium ion binding site
2353	Q	203	1	yes	no	-3.5	-1.8	-	-	no
2354	N	49	0.312	yes	no	-3.5	-1.8	-	-	no
2355	P	51	0.375	yes	no	1.6	-2.12	106	AL7	no
2356	K	166	0.81	yes	no	-3.9	-1.07	106	AL7	no
2357	Y	58	0.261	yes	no	-1.3	-0.5	106	AL7	no
2358	I	7	-	no	-	-	-	106	AL7	no
2359	T	43	0.303	yes	no	-0.7	-1.02	106	AL7	no
2360	A	32	0.302	yes	no	1.8	-0.36	106	AL7	no
2361	A	0	-	no	-	-	-	106	AL7	no
2362	C	3	-	no	-	-	-	106	AL7	no
2363	E	101	0.521	yes	no	-3.5	0.5	106	AL7	no
2364	M	28	0.149	no	-	1.9	-0.3	106	AL7	no
2365	V	1	-	no	-	-	-	106	AL7	no
2366	A	14	0.132	no	-	1.8	-0.83	106	AL7	no
2367	E	53	0.273	yes	no	-3.5	-0.82	106	AL7	no
2368	M	0	-	no	-	-	-	106	AL7	no
2369	V	0	-	no	-	-	-	106	AL7	no
2370	E	62	0.32	yes	no	-3.5	-3.5	106	AL7	no
2371	S	0	-	no	-	-	-	106	AL7	no
2372	L	3	-	no	-	-	-	-	-	no
2373	Q	43	0.47	yes	no	-3.5	-0.83	-	-	no
2374	S	15	0.115	no	-	-0.8	-0.56	-	-	no
2375	V	10	0.07	no	-	4.2	-0.56	-	-	no
2376	L	0	-	no	-	-	-	-	-	no
2377	A	7	0.179	no	-	1.8	0.38	-	-	no
2378	L	67	0.701	yes	yes	3.8	0.5	-	-	no
2379	G	7	0.976	yes	yes	-0.4	0.07	-	-	no
2380	H	36	0.402	yes	yes	-3.2	-1.41	107	AL8	no
2381	K	139	0.766	yes	yes	-3.9	-3.77	107	AL8	no
2382	R	65	0.262	yes	yes	-4.5	-1.79	107	AL8	no
2383	N	9	0.07	no	-	-3.5	-1.73	107	AL8	no
2384	S	95	0.854	yes	yes	-0.8	-2.3	107	AL8	no
2385	G	39	0.571	yes	no	-0.4	-0.73	-	-	no

#	Residue	ACC	RSA	Surface	Interface	KD	KD mean	Helix number	Helix label	Calcium ion binding site
2386	V	5	-	no	-	-	-	-	-	no
2387	P	1	0.478	yes	yes	1.6	2.07	-	-	no
2388	A	24	0.481	yes	no	1.8	2.07	-	-	no
2389	F	14	0.376	yes	yes	2.8	2.07	-	-	no
2390	L	1	-	no	-	-	-	108	AL9	no
2391	T	1	-	no	-	-	-	108	AL9	no
2392	P	49	0.36	yes	no	1.6	-0.37	108	AL9	no
2393	L	3	-	no	-	-	-	108	AL9	no
2394	L	3	-	no	-	-	-	108	AL9	no
2395	R	69	0.306	yes	yes	-4.5	-2.13	108	AL9	no
2396	N	19	0.121	no	-	-3.5	-0.42	108	AL9	no
2397	I	0	-	no	-	-	-	108	AL9	no
2398	I	2	-	no	-	-	-	108	AL9	no
2399	I	18	0.107	no	-	4.5	0.5	108	AL9	no
2400	S	0	-	no	-	-	-	108	AL9	no
2401	L	0	-	no	-	-	-	108	AL9	no
2402	A	0	-	no	-	-	-	108	AL9	no
2403	R	10	-	no	-	-	-	108	AL9	no
2404	L	1	-	no	-	-	-	109	AM1	no
2405	P	61	0.449	yes	no	1.6	-0.95	109	AM1	no
2406	L	4	-	no	-	-	-	109	AM1	no
2407	V	0	-	no	-	-	-	109	AM1	no
2408	N	9	0.057	no	-	-3.5	-1	109	AM1	no
2409	S	39	0.3	yes	no	-0.8	-0.2	109	AM1	no
2410	Y	30	0.135	no	-	-1.3	-1.87	109	AM1	no
2411	T	0	-	no	-	-	-	109	AM1	no
2412	R	8	-	no	-	-	-	109	AM1	yes
2413	V	4	-	no	-	-	-	-	-	no
2414	P	0	-	no	-	-	-	110	AM2	no
2415	P	27	0.199	no	-	1.6	1.5	110	AM2	no
2416	L	48	0.299	yes	yes	3.8	0.15	110	AM2	no
2417	V	0	-	no	-	-	-	110	AM2	no
2418	W	72	0.317	yes	no	-0.9	0.58	110	AM2	no

#	Residue	ACC	RSA	Surface	Interface	KD	KD mean	Helix number	Helix label	Calcium ion binding site
2419	K	137	0.668	yes	yes	-3.9	0.7	110	AM2	no
2420	L	65	0.396	yes	yes	3.8	-0.46	110	AM2	no
2421	G	82	0.976	yes	no	-0.4	0.83	-	-	no
2422	W	38	0.167	no	-	-0.9	0.16	-	-	no
2423	S	105	0.808	yes	no	-0.8	-0.03	-	-	no
2424	P	43	0.316	yes	no	1.6	-1.03	-	-	no
2425	K	196	0.956	yes	no	-3.9	-0.23	-	-	no
2426	P	40	0.294	yes	no	1.6	0.25	-	-	no
2427	G	57	0.679	yes	no	-0.4	0.44	-	-	no
2428	G	40	0.476	yes	no	-0.4	-0.02	-	-	no
2429	D	122	0.748	yes	no	-3.5	-0.37	-	-	no
2430	F	23	0.117	no	-	2.8	-0.38	-	-	no
2431	G	14	0.167	no	-	-0.4	0.64	-	-	no
2432	T	7	-	no	-	-	-	-	-	no
2433	A	36	0.34	yes	no	1.8	0.77	-	-	no
2434	F	36	0.183	no	-	2.8	1.4	-	-	no
2435	P	68	0.5	yes	no	1.6	0.3	-	-	no
2436	E	50	0.258	yes	no	-3.5	-0.95	-	-	yes
2437	I	7	-	no	-	-	-	-	-	no
2438	P	33	0.243	yes	no	1.6	1.28	111	AM3	no
2439	V	51	0.359	yes	no	4.2	1.28	111	AM3	no
2440	E	138	0.711	yes	no	-3.5	0.32	111	AM3	no
2441	F	45	0.228	yes	no	2.8	1.27	111	AM3	no
2442	L	0	-	no	-	-	-	111	AM3	no
2443	Q	74	0.374	yes	no	-3.5	-3.5	-	-	no
2444	E	107	0.552	yes	no	-3.5	-3.6	112	AM4	no
2445	K	80	0.39	yes	no	-3.9	-3.63	112	AM4	no
2446	E	100	0.546	yes	no	-3.5	-3.7	112	AM4	no
2447	V	1	-	no	-	-	-	112	AM4	no
2448	F	0	-	no	-	-	-	112	AM4	no
2449	K	34	0.483	yes	no	-3.9	-1.6	112	AM4	no
2450	E	24	0.227	yes	no	-3.5	-1.05	112	AM4	no
2451	F	1	-	no	-	-	-	112	AM4	no

#	Residue	ACC	RSA	Surface	Interface	KD	KD mean	Helix number	Helix label	Calcium ion binding site
2452	I	2	0.13	no	-	4.5	-1.54	112	AM4	no
2453	Y	3	0.216	yes	no	-1.3	-0.9	112	AM4	no
2454	R	3	-	no	-	-	-	112	AM4	no
2455	I	8	-	no	-	-	-	112	AM4	no
2456	N	9	0.363	yes	no	-3.5	-0.25	112	AM4	no
2457	T	8	0.148	no	-	-0.7	-1.83	112	AM4	no
2458	L	1	-	no	-	-	-	112	AM4	no
2459	G	3	-	no	-	-	-	112	AM4	no
2460	W	11	-	no	-	-	-	-	-	no
2461	T	54	0.38	yes	no	-0.7	-0.75	-	-	no
2462	S	51	0.392	yes	no	-0.8	-2.04	113	AM5	no
2463	R	100	0.403	yes	no	-4.5	-2.38	113	AM5	no
2464	T	84	0.592	yes	no	-0.7	-2.6	113	AM5	no
2465	Q	13	0.066	no	-	-3.5	-2.75	113	AM5	no
2466	F	3	-	no	-	-	-	113	AM5	no
2467	E	78	0.402	yes	no	-3.5	-2.42	113	AM5	no
2468	E	92	0.474	yes	no	-3.5	-1.92	113	AM5	yes
2469	T	1	-	no	-	-	-	113	AM5	no
2470	W	35	0.154	no	-	-0.9	-1.36	113	AM5	no
2471	A	60	0.566	yes	no	1.8	0.02	113	AM5	no
2472	T	16	0.113	no	-	-0.7	0.72	113	AM5	no
2473	L	0	-	no	-	-	-	113	AM5	no
2474	L	33	0.201	yes	yes	3.8	1.12	113	AM5	no
2475	G	33	0.393	yes	no	-0.4	1.74	113	AM5	no
2476	V	5	-	no	-	-	-	113	AM5	no
2477	L	8	-	no	-	-	-	113	AM5	no
2478	V	154	1	yes	no	4.2	1.9	113	AM5	no
2497	R	207	0.835	yes	yes	-4.5	-2.9	-	-	no
2498	T	117	0.824	yes	yes	-0.7	-2.9	-	-	no
2499	Q	90	0.455	yes	no	-3.5	-1.6	114	AM6	no
2500	I	5	-	no	-	-	-	114	AM6	no
2501	N	41	0.261	yes	no	-3.5	0.25	114	AM6	no
2502	V	25	0.176	no	-	4.2	0.25	114	AM6	no

#	Residue	ACC	RSA	Surface	Interface	KD	KD mean	Helix number	Helix label	Calcium ion binding site
2503	L	20	0.195	no	-	3.8	0.25	114	AM6	no
2504	A	0	-	no	-	-	-	114	AM6	no
2505	V	0	-	no	-	-	-	114	AM6	no
2506	Q	29	0.278	yes	no	-3.5	-0.13	114	AM6	no
2507	A	1	-	no	-	-	-	114	AM6	no
2508	I	0	-	no	-	-	-	114	AM6	no
2509	T	4	0.085	no	-	-0.7	-0.3	114	AM6	no
2510	S	1	0.308	yes	no	-0.8	0.77	114	AM6	no
2511	L	5	-	no	-	-	-	114	AM6	no
2512	V	2	-	no	-	-	-	114	AM6	no
2513	L	2	0.159	no	-	3.8	0.38	114	AM6	no
2514	S	17	0.2	yes	no	-0.8	0.48	114	AM6	no
2515	A	3	-	no	-	-	-	114	AM6	no
2516	M	1	-	no	-	-	-	-	-	no
2517	T	17	0.12	no	-	-0.7	0.79	-	-	no
2518	V	41	0.289	yes	no	4.2	0.4	-	-	no
2519	P	29	0.213	yes	no	1.6	1.72	-	-	no
2520	V	66	0.465	yes	no	4.2	0.74	-	-	no
2521	A	25	0.236	yes	no	1.8	1.8	-	-	no
2522	G	6	0.071	no	-	-0.4	0.26	-	-	no
2523	N	10	0.064	no	-	-3.5	1.01	-	-	no
2524	P	53	0.39	yes	no	1.6	0.66	-	-	no
2525	A	10	0.094	no	-	1.8	0.12	-	-	no
2526	V	30	0.211	yes	no	4.2	0.66	-	-	no
2527	S	13	0.1	no	-	-0.8	0.7	-	-	no
2528	C	12	0.089	no	-	2.5	1.2	-	-	no
2529	L	14	0.11	no	-	3.8	0.52	-	-	no
2530	E	14	0.072	no	-	-3.5	0.95	-	-	no
2531	Q	7	-	no	-	-	-	-	-	no
2532	Q	18	0.091	no	-	-3.5	0.38	-	-	no
2533	P	13	0.096	no	-	1.6	-0.87	-	-	no
2534	R	10	-	no	-	-	-	-	-	no
2535	N	10	0.064	no	-	-3.5	-2.33	-	-	no

#	Residue	ACC	RSA	Surface	Interface	KD	KD mean	Helix number	Helix label	Calcium ion binding site
2536	K	84	0.41	yes	no	-3.9	-1.93	-	-	no
2537	P	76	0.559	yes	no	1.6	0.5	-	-	no
2538	L	15	0.091	no	-	3.8	0.83	-	-	no
2539	K	171	0.834	yes	no	-3.9	1.38	-	-	no
2540	A	12	0.113	no	-	1.8	1.02	-	-	no
2541	L	24	0.146	no	-	3.8	-1.06	-	-	no
2542	D	121	0.742	yes	no	-3.5	-1.06	-	-	no
2543	T	67	0.472	yes	no	-0.7	-1	-	-	no
2544	R	231	0.931	yes	yes	-4.5	-1.46	115	AM7	no
2545	F	54	0.274	yes	no	2.8	-1.87	115	AM7	no
2546	G	6	0.071	no	-	-0.4	-1.01	115	AM7	no
2547	R	131	0.528	yes	yes	-4.5	-1.3	115	AM7	no
2548	K	65	0.317	yes	no	-3.9	-0.38	115	AM7	no
2549	L	2	-	no	-	-	-	115	AM7	no
2550	S	42	0.323	yes	no	-0.8	-1.02	115	AM7	no
2551	I	98	0.58	yes	yes	4.5	-0.15	115	AM7	no
2552	I	0	-	no	-	-	-	115	AM7	no
2553	R	1	-	no	-	-	-	115	AM7	no
2554	G	28	0.333	yes	no	-0.4	1.95	115	AM7	no
2555	I	30	0.178	no	-	4.5	0.2	115	AM7	no
2556	V	0	-	no	-	-	-	115	AM7	no
2557	E	8	-	no	-	-	-	115	AM7	no
2558	Q	73	0.419	yes	yes	-3.5	-1.5	115	AM7	no
2559	E	7	0.082	no	-	-3.5	-2.17	115	AM7	no
2560	I	2	-	no	-	-	-	115	AM7	no
2561	Q	14	0.071	no	-	-3.5	-0.49	115	AM7	no
2562	A	10	0.245	yes	no	1.8	0.1	115	AM7	no
2563	M	40	0.356	yes	no	1.9	1.1	115	AM7	no
2564	V	33	0.254	yes	no	4.2	0.45	115	AM7	no
2565	S	2	0.108	no	-	-0.8	-0.73	115	AM7	no
2566	K	81	0.58	yes	no	-3.9	-2.32	-	-	yes
2567	R	55	0.298	yes	no	-4.5	-1.73	-	-	no
2568	E	76	0.392	yes	no	-3.5	-2.18	-	-	no

#	Residue	ACC	RSA	Surface	Interface	KD	KD mean	Helix number	Helix label	Calcium ion binding site
2569	N	28	0.178	no	-	-3.5	-1.75	-	-	yes
2570	I	11	0.065	no	-	4.5	-1.04	-	-	no
2571	A	13	0.123	no	-	1.8	2.63	-	-	no
2572	T	2	-	no	-	-	-	-	-	no
2573	H	49	0.391	yes	no	-3.2	-3.2	-	-	no
2574	H	15	0.082	no	-	-3.2	-0.87	-	-	no
2575	L	62	0.555	yes	no	3.8	-0.48	-	-	no
2576	Y	55	0.518	yes	no	-1.3	0.2	-	-	no
2577	Q	20	0.101	no	-	-3.5	0.2	-	-	no
2578	A	24	0.226	yes	no	1.8	-0.98	-	-	no
2579	W	51	0.225	yes	yes	-0.9	-0.77	-	-	no
2580	D	8	-	no	-	-	-	-	-	no
2581	P	0	-	no	-	-	-	-	-	no
2582	V	2	-	no	-	-	-	-	-	no
2583	P	5	-	no	-	-	-	-	-	no
2584	S	41	0.315	yes	yes	-0.8	1.7	-	-	no
2585	L	53	0.323	yes	yes	3.8	0.45	-	-	no
2586	S	141	1	yes	yes	-0.8	0.73	-	-	no
2591	G	88	1	yes	no	-0.4	1.1	-	-	no
2592	A	74	0.698	yes	yes	1.8	1.1	-	-	no
2593	L	65	0.396	yes	yes	3.8	2.32	-	-	no
2594	I	44	0.26	yes	yes	4.5	1.68	-	-	no
2595	S	58	0.446	yes	no	-0.8	-1.38	116	AM8	no
2596	H	31	0.168	no	-	-3.2	-2.46	116	AM8	no
2597	E	126	0.649	yes	no	-3.5	-1.52	116	AM8	no
2598	K	142	0.693	yes	no	-3.9	-1.52	116	AM8	no
2599	L	5	-	no	-	-	-	116	AM8	no
2600	L	23	0.14	no	-	3.8	-0.66	-	-	no
2601	L	11	0.067	no	-	3.8	1.37	-	-	no
2602	Q	24	0.121	no	-	-3.5	1.02	-	-	no
2603	I	30	0.178	no	-	4.5	-0.22	-	-	no
2604	N	9	0.057	no	-	-3.5	1.05	-	-	no
2605	P	18	0.132	no	-	1.6	-0.97	-	-	no

#	Residue	ACC	RSA	Surface	Interface	KD	KD mean	Helix number	Helix label	Calcium ion binding site
2606	E	0	-	no	-	-	-	-	-	yes
2607	R	17	0.069	no	-	-4.5	-2.13	-	-	no
2608	E	34	0.206	yes	no	-3.5	-0.1	-	-	no
2609	L	33	0.201	yes	no	3.8	-0.34	-	-	no
2610	G	8	0.095	no	-	-0.4	-1.1	-	-	yes
2611	S	8	0.062	no	-	-0.8	-0.48	-	-	no
2612	M	11	0.059	no	-	1.9	0.1	-	-	yes
2613	S	39	0.485	yes	no	-0.8	-0.07	-	-	no
2614	Y	6	0.252	yes	no	-1.3	-2	-	-	no
2615	K	18	0.112	no	-	-3.9	-0.45	-	-	yes
2616	L	9	0.244	yes	no	3.8	-0.17	-	-	no
2617	G	6	0.071	no	-	-0.4	0.92	-	-	no
2618	Q	2	-	no	-	-	-	-	-	no
2619	V	13	0.092	no	-	4.2	-0.12	-	-	no
2620	S	8	0.062	no	-	-0.8	-0.42	117	AM9	no
2621	I	2	-	no	-	-	-	117	AM9	no
2622	H	52	0.283	yes	no	-3.2	0.38	117	AM9	no
2623	S	12	0.092	no	-	-0.8	0.27	117	AM9	no
2624	V	13	0.092	no	-	4.2	-0.11	117	AM9	no
2625	W	27	0.119	no	-	-0.9	0.97	117	AM9	no
2626	L	113	0.689	yes	no	3.8	-0.2	117	AM9	no
2627	G	51	0.607	yes	no	-0.4	0.42	117	AM9	no
2628	N	35	0.223	yes	no	-3.5	0.43	117	AM9	no
2629	S	103	0.792	yes	no	-0.8	0.45	-	-	no
2630	I	9	0.053	no	-	4.5	0.74	-	-	no
2631	T	121	0.852	yes	no	-0.7	1.8	-	-	no
2632	P	82	0.603	yes	no	1.6	1.76	-	-	no
2663	K	199	0.971	yes	no	-3.9	-3.55	-	-	no
2664	H	68	0.37	yes	no	-3.2	-3.66	-	-	no
2665	R	70	0.282	yes	no	-4.5	-1.58	-	-	no
2666	A	52	0.491	yes	no	1.8	-0.33	-	-	no
2667	G	47	0.56	yes	no	-0.4	-1.03	-	-	no
2668	V	3	-	no	-	-	-	-	-	no

#	Residue	ACC	RSA	Surface	Interface	KD	KD mean	Helix number	Helix label	Calcium ion binding site
2669	D	50	0.307	yes	no	-3.5	-2.68	118	AN1	no
2670	I	1	-	no	-	-	-	118	AN1	no
2671	H	66	0.359	yes	no	-3.2	-2.67	118	AN1	no
2672	S	56	0.431	yes	no	-0.8	-1.64	118	AN1	no
2673	C	4	-	no	-	-	-	118	AN1	no
2674	S	2	-	no	-	-	-	118	AN1	no
2675	Q	50	0.253	yes	no	-3.5	-0.5	118	AN1	no
2676	F	87	0.442	yes	yes	2.8	-1.25	118	AN1	no
2677	L	2	-	no	-	-	-	118	AN1	no
2678	L	1	-	no	-	-	-	118	AN1	no
2679	E	105	0.541	yes	yes	-3.5	0.57	118	AN1	no
2680	L	18	0.11	no	-	3.8	-1.25	118	AN1	no
2681	Y	1	-	no	-	-	-	118	AN1	no
2682	S	21	0.162	no	-	-0.8	-1.18	118	AN1	no
2683	R	167	0.673	yes	yes	-4.5	-0.6	118	AN1	no
2684	W	51	0.225	yes	yes	-0.9	-0.16	-	-	no
2685	I	4	-	no	-	-	-	-	-	no
2686	L	62	0.378	yes	yes	3.8	1.5	-	-	no
2687	P	89	0.654	yes	no	1.6	1.5	-	-	no
2693	R	279	1	yes	yes	-4.5	-2.6	-	-	no
2694	T	27	0.19	no	-	-0.7	-1.2	-	-	no
2695	P	71	0.522	yes	no	1.6	2.2	119	AN2	no
2696	A	48	0.453	yes	yes	1.8	3.24	119	AN2	no
2697	I	108	0.639	yes	yes	4.5	2.57	119	AN2	no
2698	L	11	0.067	no	-	3.8	1.7	119	AN2	no
2699	I	12	0.071	no	-	4.5	1.72	119	AN2	no
2700	S	9	0.069	no	-	-0.8	1.7	119	AN2	no
2701	E	35	0.18	no	-	-3.5	-0.1	119	AN2	no
2702	V	0	-	no	-	-	-	119	AN2	no
2703	V	0	-	no	-	-	-	119	AN2	no
2704	R	19	0.403	yes	yes	-4.5	-3.1	119	AN2	no
2705	S	2	-	no	-	-	-	119	AN2	no
2706	L	2	-	no	-	-	-	119	AN2	no

#	Residue	ACC	RSA	Surface	Interface	KD	KD mean	Helix number	Helix label	Calcium ion binding site
2707	L	2	-	no	-	-	-	119	AN2	no
2708	V	8	0.134	no	-	4.2	4.2	119	AN2	no
2709	V	4	-	no	-	-	-	119	AN2	no
2710	S	2	-	no	-	-	-	119	AN2	no
2711	D	13	0.08	no	-	-3.5	-3.5	-	-	no
2712	L	2	-	no	-	-	-	-	-	no
2713	F	5	-	no	-	-	-	-	-	no
2714	T	14	0.099	no	-	-0.7	-2.57	-	-	no
2715	E	85	0.438	yes	no	-3.5	-3.05	120	AN3	no
2716	R	79	0.319	yes	no	-4.5	-3.83	120	AN3	no
2717	N	86	0.548	yes	no	-3.5	-3.75	120	AN3	no
2718	Q	1	-	no	-	-	-	120	AN3	no
2719	F	1	-	no	-	-	-	120	AN3	no
2720	E	87	0.448	yes	no	-3.5	-1.07	120	AN3	no
2721	L	67	0.409	yes	no	3.8	1.5	120	AN3	no
2722	M	1	-	no	-	-	-	120	AN3	no
2723	Y	6	-	no	-	-	-	120	AN3	no
2724	V	84	0.592	yes	yes	4.2	2.43	120	AN3	no
2725	T	5	-	no	-	-	-	120	AN3	no
2726	L	0	-	no	-	-	-	120	AN3	no
2727	T	30	0.211	yes	no	-0.7	-1.12	120	AN3	no
2728	E	79	0.407	yes	no	-3.5	-3.3	120	AN3	no
2729	L	1	-	no	-	-	-	120	AN3	no
2730	R	56	0.226	yes	no	-4.5	-2.03	120	AN3	no
2731	R	194	0.782	yes	yes	-4.5	-2.3	120	AN3	no
2732	V	77	0.542	yes	yes	4.2	-2	120	AN3	no
2733	H	35	0.19	no	-	-3.2	-1.28	120	AN3	no
2734	P	79	0.581	yes	no	1.6	-1.48	-	-	no
2735	S	115	0.885	yes	no	-0.8	-0.9	-	-	no
2736	E	143	0.737	yes	yes	-3.5	-1.55	-	-	no
2737	D	52	0.319	yes	yes	-3.5	-1.5	-	-	yes
2738	E	147	0.758	yes	yes	-3.5	-0.83	-	-	yes
2739	I	69	0.408	yes	yes	4.5	-0.84	-	-	no

#	Residue	ACC	RSA	Surface	Interface	KD	KD mean	Helix number	Helix label	Calcium ion binding site
2740	L	5	-	no	-	-	-	121	AN4	no
2741	A	15	0.142	no	-	1.8	1.06	121	AN4	no
2742	Q	9	0.177	no	-	-3.5	1.15	121	AN4	no
2743	Y	8	0.189	no	-	-1.3	-0.74	121	AN4	no
2744	L	14	0.085	no	-	3.8	0.2	121	AN4	no
2745	V	6	-	no	-	-	-	121	AN4	no
2746	P	0	-	no	-	-	-	121	AN4	no
2747	A	0	-	no	-	-	-	121	AN4	no
2748	T	0	-	no	-	-	-	121	AN4	no
2749	C	0	-	no	-	-	-	121	AN4	no
2750	K	4	-	no	-	-	-	121	AN4	no
2751	A	0	-	no	-	-	-	121	AN4	no
2752	A	0	-	no	-	-	-	121	AN4	no
2753	A	0	-	no	-	-	-	121	AN4	no
2754	V	1	-	no	-	-	-	121	AN4	no
2755	L	18	0.11	no	-	3.8	1.67	121	AN4	no
2756	G	7	0.083	no	-	-0.4	1.67	-	-	no
2757	M	8	-	no	-	-	-	-	-	yes
2758	D	69	0.423	yes	yes	-3.5	-0.95	-	-	yes
2759	K	94	0.459	yes	yes	-3.9	-0.94	-	-	yes
2760	A	85	0.802	yes	no	1.8	0.08	-	-	no
2761	V	99	0.697	yes	no	4.2	0.08	-	-	no
2762	A	9	0.085	no	-	1.8	-0.21	-	-	no
2763	E	73	0.376	yes	no	-3.5	-0.55	-	-	yes
2764	P	39	0.287	yes	no	1.6	-1.08	122	AN5	no
2765	V	1	-	no	-	-	-	122	AN5	no
2766	S	15	0.115	no	-	-0.8	0.03	122	AN5	no
2767	R	171	0.69	yes	no	-4.5	-0.68	122	AN5	no
2768	L	27	0.165	no	-	3.8	-1.08	122	AN5	no
2769	L	0	-	no	-	-	-	122	AN5	no
2770	E	53	0.273	yes	no	-3.5	-0.32	122	AN5	no
2771	S	80	0.615	yes	no	-0.8	-0.32	122	AN5	no
2772	T	8	0.056	no	-	-0.7	-0.9	122	AN5	no

#	Residue	ACC	RSA	Surface	Interface	KD	KD mean	Helix number	Helix label	Calcium ion binding site
2773	L	13	0.079	no	-	3.8	-1.57	122	AN5	no
2774	R	177	0.714	yes	no	-4.5	-0.6	122	AN5	no
2775	S	47	0.362	yes	no	-0.8	-1.03	-	-	no
2776	S	133	1	yes	no	-0.8	-1.6	-	-	no
2777	H	60	0.326	yes	no	-3.2	0.12	123	AN6	no
2778	L	31	0.201	yes	no	3.8	-0.58	123	AN6	no
2779	P	24	0.449	yes	no	1.6	-0.27	123	AN6	no
2780	S	3	-	no	-	-	-	123	AN6	no
2781	R	52	0.21	yes	no	-4.5	1.37	123	AN6	no
2782	V	9	0.12	no	-	4.2	-0.47	123	AN6	no
2783	G	3	-	no	-	-	-	123	AN6	no
2784	A	0	-	no	-	-	-	123	AN6	no
2785	L	1	-	no	-	-	-	123	AN6	no
2786	H	10	0.054	no	-	-3.2	-3.2	123	AN6	no
2787	G	0	-	no	-	-	-	123	AN6	no
2788	I	2	-	no	-	-	-	123	AN6	no
2789	L	1	-	no	-	-	-	123	AN6	no
2790	Y	7	-	no	-	-	-	123	AN6	no
2791	V	1	-	no	-	-	-	123	AN6	no
2792	L	1	-	no	-	-	-	123	AN6	no
2793	E	10	0.052	no	-	-3.5	-0.5	123	AN6	no
2794	C	55	0.407	yes	no	2.5	-0.5	-	-	no
2795	D	4	-	no	-	-	-	-	-	no
2796	L	105	0.64	yes	no	3.8	1.37	-	-	yes
2797	L	11	0.067	no	-	3.8	0.15	-	-	no
2798	D	20	0.123	no	-	-3.5	-1.36	-	-	yes
2799	D	83	0.509	yes	no	-3.5	-0.02	-	-	yes
2800	T	91	0.641	yes	no	-0.7	-1.48	-	-	yes
2801	A	34	0.321	yes	no	1.8	-1.96	-	-	no
2802	K	131	0.639	yes	no	-3.9	-1.87	-	-	no
2803	Q	148	0.747	yes	no	-3.5	-1	124	AN7	no
2804	L	6	-	no	-	-	-	124	AN7	no
2805	I	4	-	no	-	-	-	124	AN7	no

#	Residue	ACC	RSA	Surface	Interface	KD	KD mean	Helix number	Helix label	Calcium ion binding site
2806	P	55	0.404	yes	no	1.6	-0.4	124	AN7	no
2807	V	32	0.225	yes	no	4.2	0.38	124	AN7	no
2808	I	1	-	no	-	-	-	124	AN7	no
2809	S	13	0.1	no	-	-0.8	0.04	124	AN7	no
2810	D	79	0.485	yes	no	-3.5	0.46	124	AN7	no
2811	Y	21	0.095	no	-	-1.3	-1.02	124	AN7	no
2812	L	0	-	no	-	-	-	124	AN7	no
2813	L	14	0.085	no	-	3.8	-1.06	124	AN7	no
2814	S	73	0.562	yes	no	-0.8	-1.53	124	AN7	no
2815	N	45	0.287	yes	no	-3.5	-1.02	124	AN7	no
2816	L	3	-	no	-	-	-	124	AN7	no
2817	K	135	0.659	yes	no	-3.9	-0.82	124	AN7	no
2818	G	47	0.56	yes	yes	-0.4	-0.3	124	AN7	no
2819	I	14	0.083	no	-	4.5	0.22	124	AN7	no
2820	A	13	0.123	no	-	1.8	1.57	-	-	no
2821	H	161	0.875	yes	no	-3.2	1.4	-	-	no
2822	C	62	0.459	yes	yes	2.5	0.42	-	-	no
2823	V	2	-	no	-	-	-	-	-	no
2824	N	33	0.465	yes	no	-3.5	0.07	-	-	no
2825	I	94	0.882	yes	yes	4.5	-0.73	-	-	no
2826	H	79	0.429	yes	no	-3.2	-0.75	-	-	no
2827	S	42	0.369	yes	no	-0.8	-2.84	125	AN8	no
2828	Q	44	0.399	yes	no	-3.5	-2.75	125	AN8	no
2829	Q	43	0.328	yes	no	-3.5	-2.75	125	AN8	no
2830	H	18	0.098	no	-	-3.2	-1.36	125	AN8	no
2831	V	1	-	no	-	-	-	125	AN8	no
2832	L	3	-	no	-	-	-	125	AN8	no
2833	V	0	-	no	-	-	-	125	AN8	no
2834	M	0	-	no	-	-	-	125	AN8	no
2835	C	5	-	no	-	-	-	125	AN8	no
2836	A	2	-	no	-	-	-	125	AN8	no
2837	T	0	-	no	-	-	-	125	AN8	no
2838	A	0	-	no	-	-	-	125	AN8	no

#	Residue	ACC	RSA	Surface	Interface	KD	KD mean	Helix number	Helix label	Calcium ion binding site
2839	F	7	-	no	-	-	-	125	AN8	no
2840	Y	2	-	no	-	-	-	125	AN8	no
2841	L	0	-	no	-	-	-	125	AN8	no
2842	I	2	-	no	-	-	-	125	AN8	no
2843	E	22	0.113	no	-	-3.5	-2.95	125	AN8	no
2844	N	65	0.414	yes	no	-3.5	-2.77	125	AN8	no
2845	Y	38	0.171	no	-	-1.3	-0.58	125	AN8	no
2846	P	54	0.397	yes	no	1.6	0.96	-	-	no
2847	L	154	0.939	yes	no	3.8	0.96	-	-	no
2848	D	74	0.454	yes	no	-3.5	1.52	-	-	no
2849	V	8	0.056	no	-	4.2	1.14	-	-	no
2850	G	35	0.417	yes	no	-0.4	1.15	-	-	no
2851	P	131	0.963	yes	no	1.6	-0.77	-	-	no
2852	E	119	0.613	yes	no	-3.5	-0.95	-	-	no
2853	F	6	-	no	-	-	-	-	-	no
2854	S	12	0.092	no	-	-0.8	-0.05	-	-	no
2855	A	37	0.349	yes	no	1.8	0.07	126	AN9	no
2856	S	34	0.262	yes	no	-0.8	-0.82	126	AN9	no
2857	I	2	-	no	-	-	-	126	AN9	no
2858	I	1	-	no	-	-	-	126	AN9	no
2859	Q	91	0.46	yes	no	-3.5	-0.7	126	AN9	no
2860	M	15	0.08	no	-	1.9	0.55	126	AN9	no
2861	C	2	-	no	-	-	-	126	AN9	no
2862	G	8	0.095	no	-	-0.4	0.55	126	AN9	no
2863	V	62	0.437	yes	no	4.2	1.34	126	AN9	no
2864	M	2	-	no	-	-	-	126	AN9	no
2865	L	2	-	no	-	-	-	126	AN9	no
2866	S	54	0.415	yes	no	-0.8	1	126	AN9	no
2867	G	19	0.226	yes	no	-0.4	-0.7	-	-	no
2868	S	53	0.408	yes	no	-0.8	-1.8	-	-	no
2869	E	146	0.768	yes	no	-3.5	-2.15	-	-	no
2870	E	120	0.675	yes	no	-3.5	-2.15	-	-	no
2871	S	25	0.192	no	-	-0.8	-1.8	-	-	no

#	Residue	ACC	RSA	Surface	Interface	KD	KD mean	Helix number	Helix label	Calcium ion binding site
2872	T	0	-	no	-	-	-	-	-	no
2873	P	21	0.154	no	-	1.6	1.77	127	AO1	no
2874	S	29	0.262	yes	no	-0.8	1.77	127	AO1	no
2875	I	39	0.284	yes	no	4.5	-0.28	127	AO1	no
2876	I	2	-	no	-	-	-	127	AO1	no
2877	Y	2	-	no	-	-	-	127	AO1	no
2878	H	14	0.076	no	-	-3.2	-0.18	127	AO1	no
2879	C	12	0.089	no	-	2.5	-0.9	127	AO1	no
2880	A	1	-	no	-	-	-	127	AO1	no
2881	L	4	-	no	-	-	-	127	AO1	no
2882	R	32	0.129	no	-	-4.5	-1.73	127	AO1	no
2883	G	4	-	no	-	-	-	127	AO1	no
2884	L	1	-	no	-	-	-	127	AO1	no
2885	E	0	-	no	-	-	-	127	AO1	no
2886	R	12	-	no	-	-	-	127	AO1	no
2887	L	5	-	no	-	-	-	127	AO1	no
2888	L	10	0.061	no	-	3.8	1.5	127	AO1	no
2889	L	2	-	no	-	-	-	127	AO1	no
2890	S	7	0.054	no	-	-0.8	-1	127	AO1	no
2891	E	117	0.603	yes	no	-3.5	-1	-	-	no
2892	Q	88	0.444	yes	no	-3.5	-1.5	-	-	no
2893	L	34	0.207	yes	no	3.8	-0.44	-	-	no
2894	S	84	0.646	yes	yes	-0.8	0.57	-	-	no
2895	R	183	0.738	yes	yes	-4.5	-0.64	-	-	no
2896	L	141	0.86	yes	yes	3.8	-0.67	-	-	no
2897	D	7	-	no	-	-	-	-	-	no
2898	A	18	0.17	no	-	1.8	0.1	128	AO2	no
2899	E	91	0.469	yes	yes	-3.5	0.17	128	AO2	no
2900	S	52	0.4	yes	yes	-0.8	0.27	128	AO2	no
2901	L	0	-	no	-	-	-	128	AO2	no
2902	V	34	0.239	yes	yes	4.2	-0.04	128	AO2	no
2903	K	141	0.688	yes	yes	-3.9	0.82	128	AO2	no
2904	L	34	0.207	yes	no	3.8	2.08	-	-	no

#	Residue	ACC	RSA	Surface	Interface	KD	KD mean	Helix number	Helix label	Calcium ion binding site
2905	S	0	-	no	-	-	-	-	-	no
2906	V	59	0.415	yes	yes	4.2	0	-	-	no
2907	D	99	0.607	yes	yes	-3.5	-1.82	-	-	no
2908	R	42	0.169	no	-	-4.5	-1.82	-	-	no
2909	V	5	-	no	-	-	-	-	-	no
2910	N	126	0.803	yes	no	-3.5	-1.82	-	-	no
2911	V	31	0.218	yes	no	4.2	-0.82	-	-	no
2912	H	93	0.821	yes	no	-3.2	0.07	-	-	no
2913	S	16	0.215	yes	no	-0.8	-0.98	129	AO3	no
2914	P	15	0.257	yes	no	1.6	-1.73	129	AO3	no
2915	H	19	0.212	yes	yes	-3.2	-1.72	129	AO3	no
2916	R	14	0.056	no	-	-4.5	-1.72	129	AO3	no
2917	A	3	-	no	-	-	-	129	AO3	no
2918	M	1	-	no	-	-	-	129	AO3	no
2919	A	1	-	no	-	-	-	129	AO3	no
2920	A	0	-	no	-	-	-	129	AO3	no
2921	L	6	-	no	-	-	-	129	AO3	no
2922	G	0	-	no	-	-	-	129	AO3	no
2923	L	0	-	no	-	-	-	129	AO3	no
2924	M	3	-	no	-	-	-	129	AO3	no
2925	L	0	-	no	-	-	-	129	AO3	no
2926	T	0	-	no	-	-	-	129	AO3	no
2927	C	6	-	no	-	-	-	129	AO3	no
2928	M	17	0.09	no	-	1.9	0.27	129	AO3	no
2929	Y	8	-	no	-	-	-	129	AO3	no
2930	T	49	0.345	yes	no	-0.7	-0.78	129	AO3	no
2931	G	63	0.75	yes	no	-0.4	-0.78	129	AO3	no
2932	K	145	0.707	yes	no	-3.9	-1.67	-	-	no
2953	V	145	1	yes	yes	4.2	3.67	-	-	no
2954	I	140	0.828	yes	yes	4.5	3.32	130	AO4	no
2955	V	86	0.606	yes	no	4.2	2.18	130	AO4	no
2956	A	10	0.094	no	-	1.8	1.23	130	AO4	no
2957	M	53	0.282	yes	yes	1.9	0.73	130	AO4	no

#	Residue	ACC	RSA	Surface	Interface	KD	KD mean	Helix number	Helix label	Calcium ion binding site
2958	E	97	0.5	yes	yes	-3.5	-0.15	130	AO4	no
2959	R	21	0.085	no	-	-4.5	-0.15	130	AO4	no
2960	V	0	-	no	-	-	-	130	AO4	no
2961	S	25	0.192	no	-	-0.8	-1.15	130	AO4	no
2962	V	29	0.204	yes	yes	4.2	-1.15	130	AO4	no
2963	L	2	-	no	-	-	-	130	AO4	no
2964	F	0	-	no	-	-	-	130	AO4	no
2965	D	50	0.307	yes	no	-3.5	-2.08	130	AO4	no
2966	R	17	0.085	no	-	-4.5	-3.36	130	AO4	no
2967	I	0	-	no	-	-	-	130	AO4	no
2968	R	50	0.238	yes	no	-4.5	-3.38	130	AO4	no
2969	K	86	0.668	yes	no	-3.9	-3.33	130	AO4	no
2970	G	0	0.083	no	-	-0.4	-2.1	130	AO4	no
2971	F	4	0.462	yes	no	2.8	1.62	131	AO5	no
2972	P	14	0.471	yes	no	1.6	0.6	131	AO5	no
2973	C	22	0.467	yes	no	2.5	0.6	131	AO5	no
2974	E	4	-	no	-	-	-	131	AO5	no
2975	A	0	-	no	-	-	-	131	AO5	no
2976	R	32	0.435	yes	no	-4.5	-0.13	131	AO5	no
2977	V	1	-	no	-	-	-	131	AO5	no
2978	V	1	-	no	-	-	-	131	AO5	no
2979	A	2	-	no	-	-	-	131	AO5	no
2980	R	48	0.379	yes	yes	-4.5	-4.5	131	AO5	no
2981	I	1	-	no	-	-	-	131	AO5	no
2982	L	0	-	no	-	-	-	131	AO5	no
2983	P	8	0.059	no	-	1.6	0.77	131	AO5	no
2984	Q	30	0.152	no	-	-3.5	-2.23	131	AO5	no
2985	F	0	-	no	-	-	-	131	AO5	no
2986	L	1	-	no	-	-	-	131	AO5	no
2987	D	75	0.46	yes	no	-3.5	-1.93	131	AO5	no
2988	D	34	0.209	yes	no	-3.5	-1.93	131	AO5	no
2989	F	13	0.066	no	-	2.8	-0.35	131	AO5	no
2990	F	25	0.127	no	-	2.8	2.4	131	AO5	no

#	Residue	ACC	RSA	Surface	Interface	KD	KD mean	Helix number	Helix label	Calcium ion binding site
2991	P	57	0.419	yes	no	1.6	-0.2	132	AO6	no
2992	P	27	0.199	no	-	1.6	-0.95	132	AO6	no
2993	Q	89	0.449	yes	no	-3.5	-0.95	132	AO6	no
2994	D	74	0.454	yes	no	-3.5	-0.95	132	AO6	no
2995	I	3	-	no	-	-	-	133	AO7	no
2996	M	0	-	no	-	-	-	133	AO7	no
2997	N	97	0.618	yes	no	-3.5	-2.6	133	AO7	no
2998	K	64	0.312	yes	no	-3.9	-2.6	133	AO7	no
2999	V	0	-	no	-	-	-	133	AO7	no
3000	I	6	-	no	-	-	-	133	AO7	no
3001	G	35	0.417	yes	no	-0.4	-1.5	133	AO7	no
3002	E	18	0.093	no	-	-3.5	-0.23	133	AO7	no
3003	F	3	-	no	-	-	-	133	AO7	no
3004	L	22	0.134	no	-	3.8	-0.22	133	AO7	no
3005	S	33	0.254	yes	no	-0.8	-1.83	-	-	no
3006	N	108	0.688	yes	no	-3.5	-2.83	-	-	no
3007	Q	143	0.747	yes	no	-3.5	-2.83	-	-	no
3008	Q	14	0.071	no	-	-3.5	-2.21	-	-	no
3009	P	29	0.478	yes	no	1.6	-1.07	-	-	no
3010	Y	13	0.144	no	-	-1.3	-0.38	134	AO8	no
3011	P	13	0.096	no	-	1.6	-0.1	134	AO8	no
3012	Q	50	0.253	yes	no	-3.5	0.82	134	AO8	no
3013	F	13	0.071	no	-	2.8	-0.22	134	AO8	no
3014	M	0	-	no	-	-	-	134	AO8	no
3015	A	0	-	no	-	-	-	134	AO8	no
3016	T	40	0.282	yes	no	-0.7	0.27	134	AO8	no
3017	V	0	-	no	-	-	-	134	AO8	no
3018	V	1	-	no	-	-	-	134	AO8	no
3019	Y	35	0.158	no	-	-1.3	-0.43	134	AO8	no
3020	K	69	0.337	yes	no	-3.9	-1.04	134	AO8	no
3021	V	11	0.077	no	-	4.2	-0.6	134	AO8	no
3022	F	0	-	no	-	-	-	134	AO8	no
3023	Q	58	0.293	yes	no	-3.5	-1.42	134	AO8	no

#	Residue	ACC	RSA	Surface	Interface	KD	KD mean	Helix number	Helix label	Calcium ion binding site
3024	T	52	0.366	yes	no	-0.7	-1.32	134	AO8	no
3025	L	5	-	no	-	-	-	134	AO8	no
3026	H	55	0.299	yes	no	-3.2	-1.84	134	AO8	no
3027	S	83	0.638	yes	no	-0.8	-1.16	-	-	no
3028	T	117	0.824	yes	no	-0.7	-0.63	-	-	no
3029	G	59	0.702	yes	no	-0.4	-1.57	-	-	no
3030	Q	42	0.212	yes	no	-3.5	-1.13	-	-	no
3031	S	63	0.485	yes	no	-0.8	-1.13	-	-	no
3032	S	56	0.431	yes	no	-0.8	-1.54	135	AO9	no
3033	M	37	0.197	no	-	1.9	-1.87	135	AO9	no
3034	V	0	-	no	-	-	-	135	AO9	no
3035	R	30	0.121	no	-	-4.5	-1.56	135	AO9	yes
3036	D	70	0.429	yes	no	-3.5	-1.02	135	AO9	no
3037	W	54	0.238	yes	no	-0.9	-0.64	135	AO9	no
3038	V	1	-	no	-	-	-	135	AO9	no
3039	M	17	0.09	no	-	1.9	0.32	135	AO9	no
3040	L	94	0.573	yes	no	3.8	2.15	135	AO9	no
3041	S	5	-	no	-	-	-	-	-	no
3042	L	114	0.695	yes	yes	3.8	2.27	-	-	no
3043	S	84	0.646	yes	yes	-0.8	-0.17	-	-	no
3044	N	81	0.516	yes	yes	-3.5	-2.15	-	-	no
3045	F	1	-	no	-	-	-	-	-	no
3046	T	14	0.099	no	-	-0.7	-2.9	-	-	no
3047	Q	147	0.742	yes	yes	-3.5	-2.9	-	-	no
3048	R	29	0.117	no	-	-4.5	-1.06	-	-	no
3049	A	104	0.981	yes	yes	1.8	0.78	-	-	no
3050	P	53	0.39	yes	no	1.6	1.13	136	AP1	no
3051	V	66	0.465	yes	yes	4.2	2.26	136	AP1	no
3052	A	24	0.226	yes	no	1.8	2.15	136	AP1	no
3053	M	30	0.16	no	-	1.9	2.38	136	AP1	no
3054	A	2	-	no	-	-	-	136	AP1	no
3055	T	6	-	no	-	-	-	136	AP1	no
3056	W	11	-	no	-	-	-	136	AP1	no

#	Residue	ACC	RSA	Surface	Interface	KD	KD mean	Helix number	Helix label	Calcium ion binding site
3057	S	0	-	no	-	-	-	136	AP1	no
3058	L	6	-	no	-	-	-	136	AP1	no
3059	S	8	0.062	no	-	-0.8	-0.8	136	AP1	no
3060	C	0	-	no	-	-	-	136	AP1	no
3061	F	2	-	no	-	-	-	136	AP1	no
3062	F	4	-	no	-	-	-	136	AP1	no
3063	V	0	-	no	-	-	-	136	AP1	no
3064	S	0	-	no	-	-	-	136	AP1	no
3065	A	0	-	no	-	-	-	136	AP1	no
3066	S	9	0.069	no	-	-0.8	-0.77	-	-	no
3067	T	73	0.514	yes	no	-0.7	-0.77	-	-	no
3068	S	22	0.169	no	-	-0.8	-0.17	-	-	no
3069	P	82	0.603	yes	no	1.6	0.43	-	-	no
3070	W	61	0.269	yes	no	-0.9	0.12	-	-	no
3071	V	1	-	no	-	-	-	137	AP2	no
3072	A	7	0.066	no	-	1.8	0.83	137	AP2	no
3073	A	5	-	no	-	-	-	137	AP2	no
3074	I	0	-	no	-	-	-	138	AP3	no
3075	L	6	-	no	-	-	-	138	AP3	no
3076	P	17	0.125	no	-	1.6	-0.28	138	AP3	no
3077	H	19	0.103	no	-	-3.2	0.52	138	AP3	no
3078	V	7	-	no	-	-	-	138	AP3	no
3079	I	18	0.107	no	-	4.5	-0.57	138	AP3	no
3080	S	39	0.3	yes	no	-0.8	-0.48	138	AP3	no
3081	R	37	0.149	no	-	-4.5	-0.53	-	-	yes
3082	M	57	0.303	yes	no	1.9	0.38	-	-	no
3083	G	34	0.405	yes	no	-0.4	-0.22	-	-	no
3084	K	111	0.541	yes	no	-3.9	-1.7	-	-	no
3085	L	68	0.415	yes	no	3.8	-0.95	-	-	no
3086	E	60	0.309	yes	no	-3.5	-1.07	139	AP4	yes
3087	Q	86	0.434	yes	no	-3.5	-1.57	139	AP4	no
3088	V	89	0.627	yes	no	4.2	-1.96	139	AP4	yes
3089	D	27	0.166	no	-	-3.5	-0.31	139	AP4	yes

#	Residue	ACC	RSA	Surface	Interface	KD	KD mean	Helix number	Helix label	Calcium ion binding site
3090	V	3	-	no	-	-	-	139	AP4	no
3091	N	72	0.459	yes	no	-3.5	0.7	139	AP4	no
3092	L	16	0.098	no	-	3.8	0.62	139	AP4	no
3093	F	0	-	no	-	-	-	139	AP4	no
3094	C	8	0.059	no	-	2.5	1.18	139	AP4	no
3095	L	23	0.14	no	-	3.8	2.35	139	AP4	no
3096	V	2	-	no	-	-	-	139	AP4	no
3097	A	0	-	no	-	-	-	139	AP4	no
3098	T	8	0.056	no	-	-0.7	0.16	139	AP4	no
3099	D	11	0.067	no	-	-3.5	-2.5	139	AP4	no
3100	F	4	-	no	-	-	-	139	AP4	no
3101	Y	14	0.063	no	-	-1.3	-2.7	139	AP4	no
3102	R	127	0.512	yes	yes	-4.5	-3.2	139	AP4	no
3103	H	104	0.565	yes	no	-3.2	-3.73	139	AP4	no
3104	Q	14	0.071	no	-	-3.5	-3.2	-	-	no
3105	I	2	-	no	-	-	-	-	-	no
3106	E	125	0.644	yes	yes	-3.5	-3.5	-	-	yes
3107	E	113	0.582	yes	yes	-3.5	-2.04	140	AP5	yes
3108	E	120	0.619	yes	yes	-3.5	-2.24	140	AP5	yes
3109	L	138	0.841	yes	yes	3.8	-2.62	140	AP5	no
3110	D	25	0.153	no	-	-3.5	-1.99	140	AP5	yes
3111	R	55	0.222	yes	no	-4.5	-1.73	140	AP5	yes
3112	R	173	0.698	yes	no	-4.5	-1.6	140	AP5	no
3113	A	47	0.443	yes	no	1.8	-2.5	140	AP5	no
3114	F	4	-	no	-	-	-	140	AP5	no
3115	Q	43	0.217	yes	no	-3.5	-1.75	140	AP5	no
3116	S	73	0.562	yes	no	-0.8	-2.1	140	AP5	no
3117	V	5	-	no	-	-	-	140	AP5	no
3118	L	4	-	no	-	-	-	140	AP5	no
3119	E	114	0.588	yes	no	-3.5	1.18	140	AP5	no
3120	V	118	0.831	yes	yes	4.2	1.68	141	AP6	no
3121	V	59	0.415	yes	yes	4.2	1.7	141	AP6	no
3122	A	49	0.462	yes	no	1.8	1.68	141	AP6	no

#	Residue	ACC	RSA	Surface	Interface	KD	KD mean	Helix number	Helix label	Calcium ion binding site
3123	A	29	0.274	yes	no	1.8	1.8	-	-	no
3124	P	97	0.713	yes	no	1.6	0.13	-	-	no
3125	G	67	0.798	yes	no	-0.4	0.55	-	-	no
3126	S	30	0.231	yes	no	-0.8	-0.95	-	-	no
3127	P	12	0.088	no	-	1.6	0.4	-	-	no
3128	Y	3	-	no	-	-	-	142	AP7	no
3129	H	73	0.397	yes	no	-3.2	-0.62	142	AP7	no
3130	R	98	0.395	yes	no	-4.5	-1.08	142	AP7	no
3131	L	1	-	no	-	-	-	142	AP7	no
3132	L	12	0.073	no	-	3.8	-0.42	142	AP7	no
3133	T	46	0.324	yes	no	-0.7	-0.68	142	AP7	no
3134	C	38	0.281	yes	no	2.5	-0.48	142	AP7	no
3135	L	1	-	no	-	-	-	142	AP7	no
3136	R	128	0.516	yes	no	-4.5	-0.4	142	AP7	no
3137	N	126	0.803	yes	no	-3.5	-0.32	142	AP7	no
3138	V	74	0.521	yes	no	4.2	-1.27	142	AP7	no

Legend. #: residue position according to 6X9O PDB file; ACC: Solvent Accessibility computed according to DSSP [28,29]; RSA: Relative Solvent Accessibility [30]; Surface: Exposed residue; Interface: Interacting residue computed with ISPRED4 [21]; KD: Kyte and Doolittle value of the exposed residue [31]; KD mean: exposed residue KD value averaged over a 6Å radius neighborhood; Helix number and Helix labels: numbering and labelling according to the 6X9O PDB file; Calcium ion binding sites: exposed residue participating in a Calcium ion binding site computed with FEATURE [41].
