# Supplementary material for: Huntingtin: A Protein with a Peculiar Solvent Accessible Surface
Source: Int J Mol Sci. 2021 Mar 12;22(6):2878. doi: 10.3390/ijms22062878 (PMC8001614; doi:10.3390/ijms22062878)
Supplement: Supplementary file 1 [file ijms-22-02878-s001.zip › Table_S2.pdf]

Table S2. Subcellular location of huntingtin interactors. High quality interacting genes derive from the huntingtin UniProt file P42858, where IntAct data are shown. Gene: Gene name. Subcellular location: as annotated in UniProt.

**Gene**	**Subcellular location**
ARHGAP24	In migrating cells, localizes to membrane lamellae and protusions; Cell junction, adherens junction; Note=Localizes to actin stress fibers; Cell junction, focal adhesion; Cell projection; Cytoplasm, cytoskeleton
BAZ1A	Nucleus; Note=May target the CHRAC complex to heterochromatin
BECN1	Endoplasmic reticulum membrane; Endosome; Expressed in dendrites and cell bodies of cerebellar Purkinje cells; Cytoplasm; Note=Interaction with ATG14 promotes translocation to autophagosomes; Endosome membrane; Golgi apparatus, trans-Golgi network membrane; Mitochondrion membrane; Cytoplasmic vesicle, autophagosome
CEP126	Midbody; Cytoplasm, cytoskeleton, cilium basal body; Cytoplasm, cytoskeleton, microtubule organizing center, centrosome
CHD3	Nucleus, PML body; Nucleus; Cytoplasm, cytoskeleton, microtubule organizing center, centrosome; Note=Associates with centrosomes in interphase and mitosis
COPB1	Endoplasmic reticulum-Golgi intermediate compartment; Cytoplasmic vesicle, COPI-coated vesicle membrane; Peripheral membrane protein; Proteolytic cleavage by CAPN8 triggers translocation from Golgi to cytoplasm; Cytoplasm; Note=The coatomer is cytoplasmic or polymerized on the cytoplasmic side of the Golgi, as well as on the vesicles/buds originating from it; Occasionally present at the trans-side of Golgi, but mainly present at the cis-Golgi side in transitional areas; Cell membrane; Golgi apparatus membrane; Some association with high-density and low-density microsomes and mitochondria/nuclei fraction; Very little found in plasma membrane fraction; Present in cytoplasm, not associated with visible coats or membranes, with a minor fraction present on small clusters of tubules and vesicles; Found in perinuclear vesicular-tubular clusters
CREBBP	Note=Recruited to nuclear bodies by SS18L1/CREST; Cytoplasm; Nucleus; In the presence of ALX1 relocalizes from the cytoplasm to the nucleus
CRMP1	Perikaryon; Cytoplasm; Following SEMA3A stimulation of DRG neurons, colocalizes with F-actin; Cytoplasm, cytoskeleton, microtubule organizing center, centrosome; Cell projection, growth cone; Colocalizes with FLNA and tubulin in the central region of DRG neuron growth cone; Cytoplasm, cytoskeleton, spindle; Note=Associated with centrosomes and the mitotic spindle during metaphase; Cytoplasm, cytoskeleton
CTNNB1	In mitosis, when NEK2 activity increases, it localizes to centrosomes at spindle poles independent of CROCC; In interphase, colocalizes with CROCC between CEP250 puncta at the proximal end of centrioles, and this localization is dependent on CROCC and CEP250; Interaction with FAM53B promotes translocation to the nucleus; Cytoplasm; Translocates to the nucleus when it is stabilized; Cell junction, adherens junction; Cytoplasm, cytoskeleton, microtubule organizing center, centrosome; Cell membrane; Cell junction; Interaction with GLIS2 and MUC1 promotes nuclear translocation; Colocalizes with CDK5 in the cell-cell contacts and plasma membrane of undifferentiated and differentiated neuroblastoma cells; Note=Colocalized with RAPGEF2 and TJP1 at cell-cell contacts; Nucleus; Cytoplasm, cytoskeleton, spindle pole; Cell junction, synapse; The majority of beta-catenin is localized to the cell membrane; Interaction with EMD inhibits nuclear localization; Cytoplasmic when it is unstabilized; Cytoplasm, cytoskeleton; Cytoplasm, cytoskeleton, cilium basal body
DCTN2	Cytoplasm, cytoskeleton, microtubule organizing center, centrosome; Membrane
DNAJC11	Mitochondrion; Note=Isoforms show differential submitochondrial localization; A 35 kDa form behaved either as an inner membrane space; A 57 kDa form;
DNAJC21	Cytoplasm; Nucleus; Nucleus, nucleolus; Note=Within the nucleus, localizes primarily to the nucleolus
DNAJC4	Membrane
DNALI1	Cell projection, cilium, flagellum; Cell projection, cilium
DNM1	Cytoplasm; Note=Microtubule-associated; Cytoplasm, cytoskeleton

Gene	Subcellular location
DYNC1H1	Cytoplasm, cytoskeleton
ECH1	Mitochondrion; Peroxisome
ELP1	Cytoplasm; Nucleus
ERCC6L	Evolution from inner centromeres to thin threads takes place in response to tension; Note=Localizes to kinetochores, inner centromeres and thin threads connecting separating chromosomes even during anaphase; Resolution of thin threads requires topoisomerase 2-alpha; Chromosome, centromere; In metaphase, it localizes to numerous thin threads that stretch between sister kinetochores of the aligned chromosomes and are composed of catenated centromeric DNA; Chromosome, centromere, kinetochore; In prometaphase cells, it mostly concentrates in between kinetochores; Chromosome
EVL	Cytoplasm, cytoskeleton, stress fiber; In activated T-cells, localizes to the F-actin collar and the distal tip of microspikes; Cell projection, lamellipodium; Cytoplasm, cytoskeleton; Note=Targeted to the leading edge of lamellipodia and the distal tip of stress fibers through interaction with a number of proteins
F8A1	Nucleus, nuclear body; Cytoplasm; Colocalized with HTT in endosomes; In absence of HTT, F8A1/F8A2/F8A3 is concentred in cytoplasm; In neuron found in intranuclear structures, the intranuclear rodlets; Nucleus; Note=Diffuse presence in the cytoplasm and accumulation in the nucleus; Early endosome
FEZ1	Note=Colocalizes with both, alpha- and gamma-tubulin; Cytoplasm, cytoskeleton, microtubule organizing center, centrosome; Cell membrane; Translocated from the plasma membrane to the cytoplasm by activation of the PKC zeta
FNBP4	
FTL	
GIT1	Cytoplasm; During cell migration, when cells detach, moves from the adhesions into the cytoplasmic complexes towards the leading edge, while, when cells adhere, it is found in vinculin-containing adhesions; Recruitment to adhesions may be mediated by active tyrosine-phosphorylated paxillin; Note=Cycles between at least 3 distinct intracellular compartments, including focal adhesions, cytoplasmic complexes and membrane protrusions
GOLPH3L	Note=Phosphatidylinositol 4-phosphate; Golgi apparatus, trans-Golgi network membrane; Golgi apparatus, Golgi stack membrane
GTF3C3	Nucleus
H3C1	Nucleus; Chromosome
HAP1	Localization to neuronal processes and neurite tips is decreased by YWHAZ; Cell junction, synapse, presynapse; Cytoplasm; Lysosome; Cell projection, dendritic spine; Endoplasmic reticulum; Cell projection, dendrite; Note=Localizes to large nonmembrane-bound cytoplasmic bodies found in various types of neurons, called stigmoid bodies; Cytoplasmic vesicle, secretory vesicle, synaptic vesicle; Nucleus; Early endosome; Mitochondrion; In the nucleus localizes to nuclear rods; Cell projection, axon; Cell projection, growth cone; Cell projection, neuron projection; Cytoplasm, cytoskeleton; Cytoplasmic vesicle, autophagosome
HEY2	Nucleus
HIP1	Note=Shuttles between cytoplasm and nucleus; Cytoplasm; Endomembrane system; Nuclear translocation can be induced by AR; Cytoplasmic vesicle, clathrin-coated vesicle membrane; Nucleus
HMG20A	Nucleus

Gene	Subcellular location
HMGB1	Endoplasmic reticulum-Golgi intermediate compartment; Endosome; Release from macrophages in the extracellular milieu requires the activation of NLRC4 or NLRP3 inflammasomes; Secreted; Shuttles between the cytoplasm and the nucleus; Cytoplasm; Found on the surface of activated platelets; Passively released to the extracellular milieu from necrotic cells by diffusion, involving the fully reduced HGMB1 which subsequently gets oxidized; Also released from apoptotic cells; Cell membrane; Note=In basal state predominantly nuclear; Nucleus; An increased chromatin association is observed when associated with the adenovirus protein pVII; Translocates from the nucleus to the cytoplasm upon autophagy stimulation; Active secretion from a variety of immune and non-immune cells such as macrophages, monocytes, neutrophils, dendritic cells and natural killer cells in response to various stimuli such as LPS and cytokines involves a nonconventional secretory process via secretory lysosomes; Secreted by plasma cells in response to LPS; Chromosome
JAKMIP1	Membrane; Peripheral membrane protein; Localizes to the cell body and neurites of hippocampal neurons where it accumulates in granules; Cytoplasm, cytoskeleton; Note=Colocalizes with the microtubule network; Localizes to the tail and to a lower extent to the head of sperm cells
LDOC1	Nucleus
MAGEB6	
MBD1	Nucleus speckle; Associated with euchromatic regions of the chromosomes, with pericentromeric regions on chromosome 1 and with telomeric regions from several chromosomes; Note=Nuclear, in a punctate pattern; Nucleus matrix; Nucleus; Chromosome
MED15	Cytoplasm; Nucleus
MED31	Nucleus
MKRN2	
MRE11	Chromosome, telomere; Nucleus; Note=Localizes to discrete nuclear foci after treatment with genotoxic agents; Chromosome
MRFAP1L1	
MTSS1	Cytoplasm, cytoskeleton
NBR1	Is targeted to lysosomes for degradation; Lysosome; Note=In cardiac muscles localizes to the sarcomeric M line; Cytoplasm; Cytoplasm, myofibril, sarcomere, M line; Cytoplasmic vesicle, autophagosome
NCOR1	Nucleus
NME4	Mitochondrion matrix; Colocalizes with OPA1 in mitochondria; Note=Predominantly localized in the mitochondrion intermembrane space; Mitochondrion intermembrane space
NUP58	Nucleus, nuclear pore complex; Central region of the nuclear pore complex, within the transporter; Note=Biased towards cytoplasmic side; Nucleus membrane
OPTN	Cytoplasm, perinuclear region; Golgi apparatus; Note=Found in the perinuclear region and associates with the Golgi apparatus; Cytoplasmic vesicle; Colocalizes with MYO6 and RAB8 at the Golgi complex and in vesicular structures close to the plasma membrane; Localizes to LC3-positive cytoplasmic vesicles upon induction of autophagy; Recycling endosome; Golgi apparatus, trans-Golgi network; Cytoplasmic vesicle, autophagosome
OSTF1	Cytoplasm
P4HA1	Endoplasmic reticulum lumen

Gene	Subcellular location
PACSIN1	Associates with membranes via its F-BAR domain; Colocalizes with DNM1 at vesicular structures in the cell body and neurites; Cytoplasm, cytosol; Cytoplasm; Cell membrane; Cell projection, ruffle membrane; Note=Colocalizes with MAPT in axons; Cell junction, synapse; Membrane; Peripheral membrane protein; Cell junction, synapse, synaptosome; Cell projection; Cytoplasmic vesicle membrane; In primary neuronal cultures, present at a high level in presynaptic nerve terminals and in the cell body
PAK2	;; Note=MYO18A mediates the cellular distribution of the PAK2-ARHGEF7-GIT1 complex to the inner surface of the cell membrane; [Serine/threonine-protein kinase PAK 2]: Cytoplasm
PFN2	Cytoplasm, cytoskeleton
PIAS4	Nucleus, PML body; Note=Colocalizes with SUMO1 and TCF7L2/TCF4 and LEF1 in a subset of PML
PIBF1	Secreted; Respective predominant forms are isoform 1 in the intracellular and the 57 kDa protein in the extracellular medium; Cytoplasm; Cytoplasm, cytoskeleton, microtubule organizing center, centrosome; Note=In progesterone-treated astrocytoma cells a 57 kDa protein and isoform 1
PIK3R1	
PIK3R2	
PIK3R3	
PKM	Nuclear translocation is sufficient to induce cell death that is caspase independent, isoform-specific and independent of its enzymatic activity; Cytoplasm; Nucleus; Note=Translocates to the nucleus in response to different apoptotic stimuli
PPARG	Cytoplasm; Note=Redistributed from the nucleus to the cytosol through a MAP2K1/MEK1-dependent manner; Nucleus; NOCT enhances its nuclear translocation
PQBP1	Nucleus speckle; Cytoplasmic granule; Localizes to cytoplasmic stress granules; Nucleus; Colocalized with ATXN1 in nuclear inclusion bodies; Colocalized with POU3F2; Note=Colocalizes with SRSF2 in nuclear speckles
PRMT2	[Isoform 1]: Cytoplasm; Nucleus; Note=Translocates from the cytoplasm to the nucleus, after hormone exposure; Excluded from nucleolus
PRPF40A	Nucleus speckle; Nucleus matrix; Note=Colocalizes with AKAP8L in the nuclear matrix
RAB5A	Endosome membrane; Cytoplasmic vesicle, phagosome membrane; Alternates between membrane-bound and cytosolic forms; Melanosome; Cell projection, ruffle; Early endosome membrane; Cell membrane; Cytoplasmic vesicle; Membrane; Note=Enriched in stage I melanosomes; Cytoplasm, cytosol
RNF20	Nucleus
RNF40	Nucleus
RPL4	
SETD2	Nucleus; Chromosome
SH3GL3	Cytoplasm; Early endosome membrane; Note=Associated with postsynaptic endosomes in hippocampal neurons; Associated with presynaptic endosomes in olfactory neurons
SH3GLB1	Following starvation colocalizes with ATG5 and LC3 autophagy-related protein; Cytoplasm; Mitochondrion outer membrane; Golgi apparatus membrane; Note=Association with the Golgi apparatus depends on the cell type; Cytoplasmic vesicle, autophagosome membrane; Midbody
SNCA	Membrane; Secreted; Cytoplasm; Note=Membrane-bound in dopaminergic neurons; Nucleus; Cell junction, synapse
SORBS1	Cell junction, adherens junction; Cell membrane; Also detected at the plasma membrane and in neuronal intranuclear inclusions; Colocalized with PXN at focal adhesions during myogenic differentiation; Nucleus matrix; Cell junction, focal adhesion; Nucleus; Colocalizes with actin stress fibers; Cytoplasm, cytoskeleton; Note=Colocalizes with the Ten-1 ICD form of TENM1 in the nucleus

Gene	Subcellular location
SQSTM1	In protein aggregate diseases of the liver, found in large amounts in Mallory bodies of alcoholic and nonalcoholic steatohepatitis, hyaline bodies in hepatocellular carcinoma, and in SERPINA1 aggregates; When nuclear export is blocked by treatment with leptomycin B, accumulates in PML bodies; In the cytoplasm, observed in both membrane-free ubiquitin-containing protein aggregates; Late endosome; Lysosome; In neurodegenerative diseases, detected in Lewy bodies in Parkinson disease, neurofibrillary tangles in Alzheimer disease, and HTT aggregates in Huntington disease; Cytoplasm, myofibril, sarcomere; Endoplasmic reticulum; Note=In cardiac muscle, localizes to the sarcomeric band; Enriched in Rosenthal fibers of pilocytic astrocytoma; Nucleus, PML body; Nucleus; Commonly found in inclusion bodies containing polyubiquitinated protein aggregates; Cytoplasm, cytosol; Co-localizes with TRIM5 in cytoplasmic bodies; Colocalizes with TRIM13 in the perinuclear endoplasmic reticulum; Cytoplasmic vesicle, autophagosome
SRGAP1	
SRGAP2	Cell junction, synapse, postsynaptic density; Cell projection, lamellipodium; Cytoplasm; Cell projection, dendritic spine; Cell junction, synapse, postsynaptic cell membrane; Cell membrane; Cytoplasmic vesicle, phagosome; Nucleus; Note=Recruited to actin-rich phagosomes during phagocytosis; Translocates from nucleus to cytoplasm during development
SRGAP3	
SRRT	Cytoplasm; Shuttles between the nucleus and the cytoplasm in a CRM1-dependent way; Nucleus, nucleoplasm; Note=Predominantly nuclear
SYVN1	Endoplasmic reticulum membrane
TACC1	Cytoplasm; Cytoplasm, cytoskeleton, microtubule organizing center, centrosome; Note=Nucleus during interphase; Nucleus; ;; Midbody; Weakly concentrated at centrosomes during mitosis and colocalizes with AURKC at the midbody during cytokinesis
TANK	Cytoplasm
TCERG1	Nucleus
TP53	Recruited into PML bodies together with CHEK2; Note=Interaction with BANP promotes nuclear localization; Translocates to mitochondria in response to mitomycin C treatment; Cytoplasm; Cytoplasm, cytoskeleton, microtubule organizing center, centrosome; Endoplasmic reticulum; Nucleus, PML body; Nucleus; Mitochondrion matrix; Translocates to mitochondria upon oxidative stress
TPR	Detected exclusively to the cytoplasmic margin of NPC; During mitosis, remains associated with the nuclear envelope until prometaphase; Anchored by NUP153 to the NPC; The assembly of the NPC is a stepwise process in which Trp-containing peripheral structures assemble after other components, including p62; Note=Detected as discrete intranuclear foci with IFI204; Detected at kinetochores during prometaphase; Nucleus membrane; Reorganized during mitosis in a viscous and dynamic nuclear-derived spindle matrix that embeds the microtubule spindle apparatus from pole to pole in a microtubule-independent manner; Associated with the mitotic spindle from late prometaphase until anaphase; Nucleus; Cytoplasm, cytoskeleton, spindle; Recruited to the reforming nuclear envelope during telophase and cytokinesis; Docking to the inner nucleoplasmic side of the NPC is mediated through binding to nucleoporins; Colocalizes with DYNLL1 at the mitotic spindle; Nucleus envelope; Nucleus, nuclear pore complex; Remains localized to the nuclear membrane after poliovirus; Chromosome, centromere, kinetochore; Detected in diffuse and discrete spheroidal intranuclear foci; Cytoplasm; In interphase, localizes to the nucleoplasmic side of the nuclear pore complex; Colocalizes with dynein, dynactin, tubulin at kinetochore during the metaphase-anaphase transition; Colocalizes with MAD2L1 in the spindle matrix but not at kinetochore; Detected as filaments that emanate from the nuclear basket of the NPC and extend to the nucleolus to delineate a chromatin-free network extending from the nuclear envelope to the perinucleolar region; Nucleocytoplasmic shuttling protein imported into the nucleus in a XPO1/CRM1- and Importin alpha/Importin beta receptor-dependent manner
TRAFD1	

Gene	Subcellular location
UBAC1	Cytoplasm
UBE2K	Cytoplasm
ULK1	Note=Under starvation conditions, is localized to puncate structures primarily representing the isolation membrane that sequesters a portion of the cytoplasm resulting in the formation of an autophagosome; Cytoplasm, cytosol; Preautophagosomal structure
USP9X	Cytoplasm; Cell projection, growth cone
WAC	Nucleus speckle; Note=In distinct nuclear speckles; Nucleus; Colocalizes with pre-mRNA processing complexes
WBP4	Nucleus speckle; Nucleus
WDFY3	Perikaryon; Nucleus membrane; In the cytosol of starved cells, colocalizes with autophagic structures; Localizes throughout neurons, including within axons; When nuclear export is blocked by treatment with leptomycin B, accumulates in nuclear bodies, that completely or partially colocalize with promyelocytic leukemia; Note=Relocalization from the nucleus to the cytosol is stimulated by cellular stress, such as starvation or proteasomal inhibition; Nucleus, PML body; In neurons, enriched in the light membrane fraction along with the synaptosomal membrane protein synaptophysin and the membrane-bound form of LC3/MAP1LC3A/MAP1LC3B, called LC3-II, a classic marker for autophagic vesicles; Membrane; Peripheral membrane protein; Cytoplasm, cytosol; Cell projection, axon; This redistribution is dependent on p62/SQSTM1
XAGE3	
XRCC6	Nucleus; Chromosome
ZDHHC13	Cytoplasmic vesicle membrane; Golgi apparatus membrane; Note=Low extracellular Mg
ZDHHC17	Cytoplasmic vesicle membrane; Golgi apparatus membrane; Cell junction, synapse, presynaptic cell membrane; Note=Low extracellular Mg
ZFC3H1	
ZFYVE19	Note=Localizes mainly on centrosomes in interphase and early mitosis; Localizes at the cleavage furrow and midbody ring in late mitosis and cytokinesis; Cytoplasm, cytoskeleton, microtubule organizing center, centrosome; Cleavage furrow; Midbody, Midbody ring
ZMAT2	Nucleus
